# *De novo* transcriptome assembly of textile hemp from datasets on hypocotyls and adult plants

**DOI:** 10.1016/j.dib.2019.104790

**Published:** 2019-11-09

**Authors:** Marc Behr, Stanley Lutts, Jean-Francois Hausman, Kjell Sergeant, Sylvain Legay, Gea Guerriero

**Affiliations:** a Environmental Research and Innovation Department, Luxembourg Institute of Science and Technology, 5, rue Bommel, Z.A.E. Robert Steichen, L-4940, Hautcharage, Luxembourg; b Groupe de Recherche en Physiologie Végétale (GRPV), Earth and Life Institute - Agronomy (ELI-A), Université catholique de Louvain (UCLouvain), 1348 Louvain-la-Neuve, Belgium

**Keywords:** Hemp, Cell wall, Transcriptome, Hypocotyl, Bast fiber

## Abstract

We here provide an updated *de novo* transcriptome of the hemp textile variety Santhica 27. The assembly was performed by merging the reads obtained previously on a time-series relative to the hypocotyl development and on bast fibers isolated from internodes of adult plants at different heights with those obtained from a newly performed transcriptome study on the hypocotyl in response to jasmonic acid treatment. More specifically, hypocotyls aged 15 days were treated with jasmonic acid and collected 3 and 5 days after the application of the plant growth regulator. RNA-Seq was then performed on the treated hypocotyls. The transcriptome reported here will be a useful resource for those scientists engaged in the study of bast fiber development, as well as cell wall biosynthesis in textile hemp. The transcriptome is also useful for molecular studies relative to the synthesis of secondary metabolites, such as phenolic compounds (e.g. flavonoids) and lignans/lignanamides.

**Table:** Specifications Table

Subject	Plant Science
Specific subject area	Investigation of the molecular factors involved in hemp bast fiber and gelatinous cell wall development
Type of data	Table Text
How data were acquired	Next-Generation sequencing, Illumina MiSeq
Data format	Raw, processed
Parameters for data collection	RNA-Seq data from three independent experiments performed on different hemp tissues under various conditions were assembled to create a *de novo* transcriptome assembly.
Description of data collection	The first experiment consists in a time-course study of hemp hypocotyl development at 6, 9, 15 and 20 days after sowing. The second experiment investigates isolated hemp bast fibers sampled at various developmental stages (from elongation to cell wall thickening). The third experiment consists in a time-course comparison of hemp hypocotyl treated with 0.1 mM jasmonic acid (3 and 5 days after treatment).
Data source location	Luxembourg Institute of Science and Technology Esch/Alzette Luxembourg
Data accessibility	The data are available as a BioProject hosted at NCBI Repository name: BioProject, NCBI Data identification number: PRJNA435671 Direct URL to data: https://www.ncbi.nlm.nih.gov/bioproject/?term=PRJNA435671

**Table:** 

**Value of the Data** • Hemp, especially its hypolignified bast fibers, is a valuable plant in the era of biosourced economy. The molecular factors regulating this hypolignification are still not fully understood. These data may contribute to decipher the regulation of this important phenotypic trait. • Researchers working on fiber crops or interested in the molecular regulation of lignification may benefit from these data. Breeders may also find genes important for stem yield and bast fiber quality. • This *de novo* transcriptome assembly may be used to map new transcriptomic data dealing with cell wall formation, regulation of secondary metabolism and primary and secondary growth in hemp.

## 1 Data

The transcriptomic data are deposited in the BioProject PRJNA435671 (https://www.ncbi.nlm.nih.gov/bioproject/?term=PRJNA435671). Table 1 summarizes the total number of reads and the corresponding RNA-Seq assembly statistics. Supplementary document 1 consists in the hemp contig sequences obtained from *de novo* transcriptome assembly using the datasets originated with the 3 experiments. Supplementary document 2 refers to the annotation. Supplementary document 3 reports the quality control metrics of the raw reads (the majority of the sequences have a PHRED score >35, indicative of high quality) obtained from the newly performed RNA-Seq on hypocotyls treated or not with jasmonic acid.

**Table 1:** Hemp *de novo* transcriptome assembly statistics.

	Length
N75	775 bp
N50	1530 bp
N25	2430 bp
Minimum	240 bp
Maximum	15,761 bp
Average	1033 bp
Count	37,083 contigs
Total	38,314,473 bp
Total reads	789,431,014

## 2 Experimental design, materials and methods

The experimental designs of the hypocotyl time course and bast fibers at various developmental stages were described in Refs. [1,2], respectively.

Hemp hypocotyls of the textile monoecious variety Santhica 27 were grown in a mixture of compost/sand (1:1 w/w) in controlled conditions. Hypocotyls were sampled 3 and 5 days after jasmonic acid-JA (0.1 mM) or mock application (PBS with 0.06% ethanol to compensate for JA solubilisation) at 15 days (H18 and H20, respectively). This experiment was performed in biological triplicate (20–25 hypocotyls for each biological replicate). Total RNA was extracted using the RNeasy Plant Mini Kit (Qiagen), and quality-checked using a NanoDrop 1000 Spectrophotometer (Thermo Scientific) and a 2100 Bioanalyzer (Agilent Life Sciences). All the RNAs displayed a RIN above 9.3. mRNAs were isolated from 5 μg of total RNA using the Magnosphere UltraPure mRNA purification kit (Takara), following the manufacturer's instruction. After separation, mRNAs were quality-checked using the Pico RNA assay and a Bioanalyzer. The residual rRNA contamination was below 5% for all the samples. The first-strand cDNA synthesis, reverse transcription and library amplification were performed with the SMARTer Stranded RNA-Seq kit (Clontech), using 8 μl of mRNA (for a quantity ranging between 24 and 42 ng of mRNA) and following the manufacturer's instruction. The enrichment step was carried out using 11 cycles of PCR. The profile of the libraries was evaluated using the High Sensitivity DNA Assay (concentration ranging from 19 to 39 nM). Indexing was performed using the Illumina indexes 1–12. Quantification was performed using the KAPA library quantification kit (KAPA) with the ViiA7 qPCR system. The pooled libraries (20 pM) were sequenced on an Illumina MiSeq in 6 consecutive runs (MiSeq reagent kit V3, 150 cycles) generating 75 base pairs (bp) paired-end reads.

Raw sequences reads were uploaded in CLC Genomics Workbench 9.0.1. Sequences were filtered and trimmed as follows: sequence length >35 bp, sequence quality score <0.01, no ambiguity in the sequence, trimming using Illumina adaptors, hard trim of 5 bp at the 5′ end and 2 bp at the 3’ end. The *de novo* assembly, with sequences from the 3 experiments, was performed with an auto wording size of 20, an auto bubble size of 50 and a minimum contig length of 300 bp. The reads were mapped back to the assembly with a mismatch, insertion and deletion cost of 3, a length fraction and similarity >0.95. The assembly was then annotated using Blast2GO PRO version 3.0 against the *Arabidopsis thaliana* non-redundant database.
