# Supplementary material for: De novo transcriptome assembly of textile hemp from datasets on hypocotyls and adult plants
Source: Data Brief. 2019 Nov 9;27:104790. doi: 10.1016/j.dib.2019.104790 (PMC6920436; doi:10.1016/j.dib.2019.104790)
Supplement: Multimedia component 1 [file mmc1.pdf]

Sequencing QC Report
Based upon: 21,460,084 sequences in 6 data sets
Generated by: Guerrier
Creation date: Mon Nov 13 14:42:45 CET 2017
Software: CLC Genomics Workbench 9.0.1

Table of contents

1. Summary 3
2. Per-sequence analysis 3
    2.1 Lengths distribution 3
    2.2 GC-content 4
    2.3 Ambiguous base-content 5
    2.4 Quality distribution 6
3. Per-base analysis 6
    3.1 Coverage 7
    3.2 Nucleotide contributions 7
    3.3 GC-content 8
    3.4 Ambiguous base-content 9
    3.5 Quality distribution 9

1. Summary

Creation date:	Mon Nov 13 14:42:45 CET 2017
Generated by:	Guerrier
Software:	CLC Genomics Workbench 9.0.1
Based upon:	6 data sets
H18CTRL1_S4_L001_R1_001 (paired):	3,297,668 sequences in pairs
H18CTRL1_S4_L001_R1_001 (paired)-1:	3,219,938 sequences in pairs
H18C1_S4_L001_R1_001 (paired):	3,454,980 sequences in pairs
H18CTRL1_S4_L001_R1_001 (paired)-4:	4,021,320 sequences in pairs
H18CTRL1_S4_L001_R1_001 (paired)-2:	3,749,652 sequences in pairs
H18CTRL1_S4_L001_R1_001 (paired)-3:	3,716,526 sequences in pairs
Total sequences in data sets	21,460,084 sequences
Total nucleotides in data sets	1,579,112,276 nucleotides

2. Per-sequence analysis

2.1 Lengths distribution

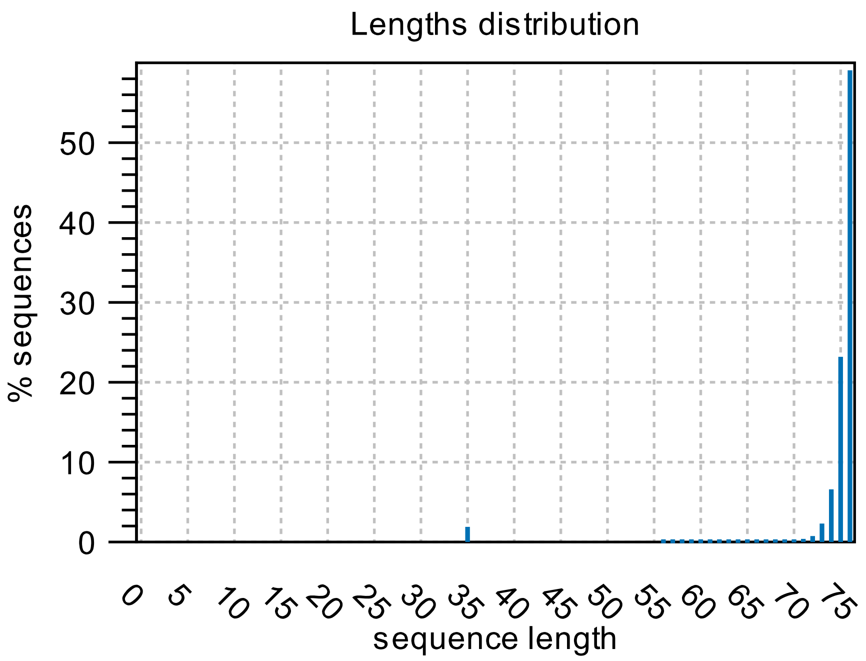

Distribution of sequence lengths. In cases of untrimmed Illumina or SOLiD reads it will just contain a single peak.
x: sequence length in base-pairs

y: number of sequences featuring a particular length normalized to the total number of sequences

2.2 GC-content

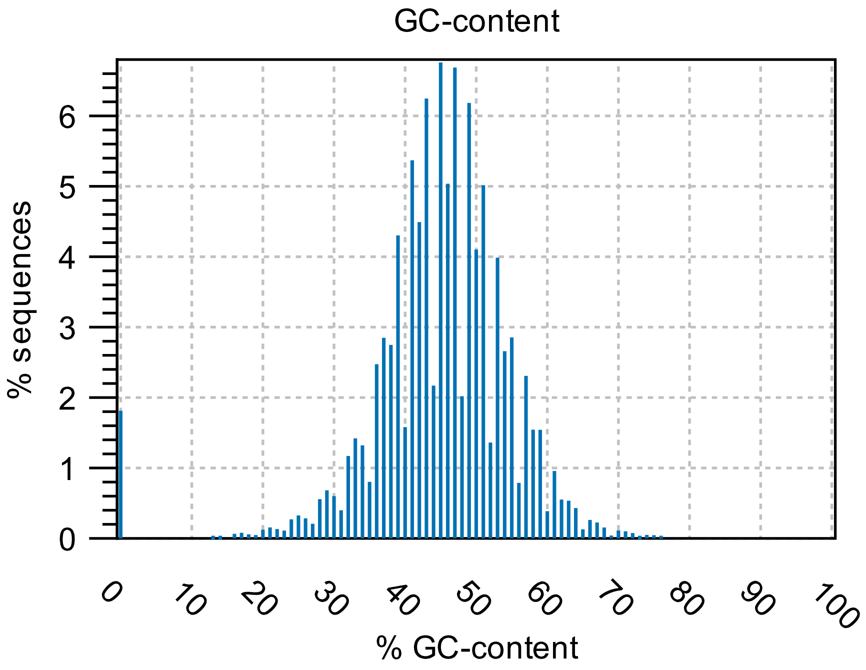

Distribution of GC-contents. The GC-content of a sequence is calculated as the number of GC-bases compared to all bases (including ambiguous bases).
x: relative GC-content of a sequence in percent
y: number of sequences featuring particular GC-percentages normalized to the total number of sequences

2.3 Ambiguous base-content

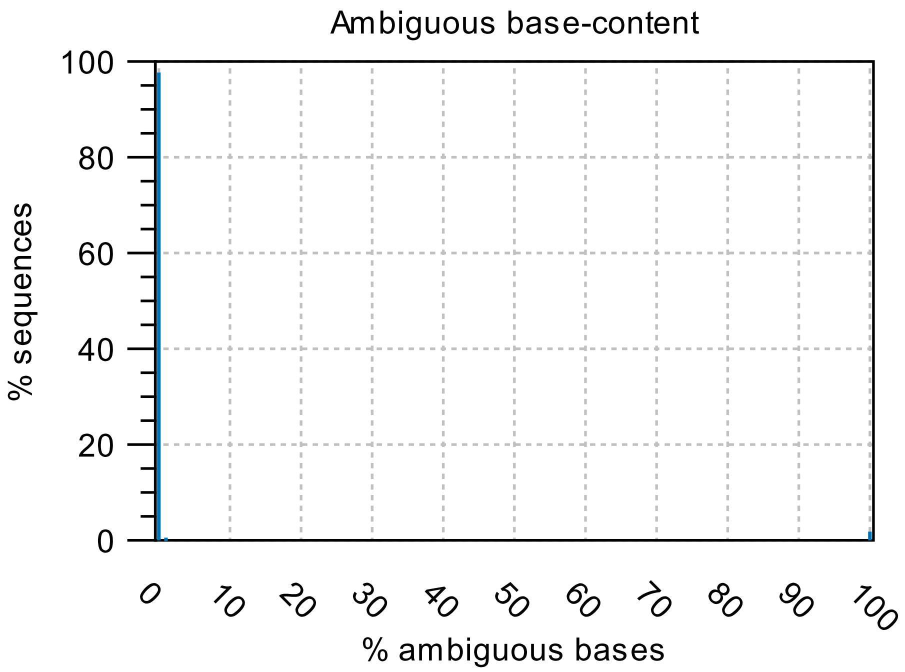

Distribution of N-contents. The N-content of a sequence is calculated as the number of ambiguous bases compared to all bases.
x: relative N-content of a sequence in percent
y: number of sequences featuring particular N-percentages normalized to the total number of sequences

2.4 Quality distribution

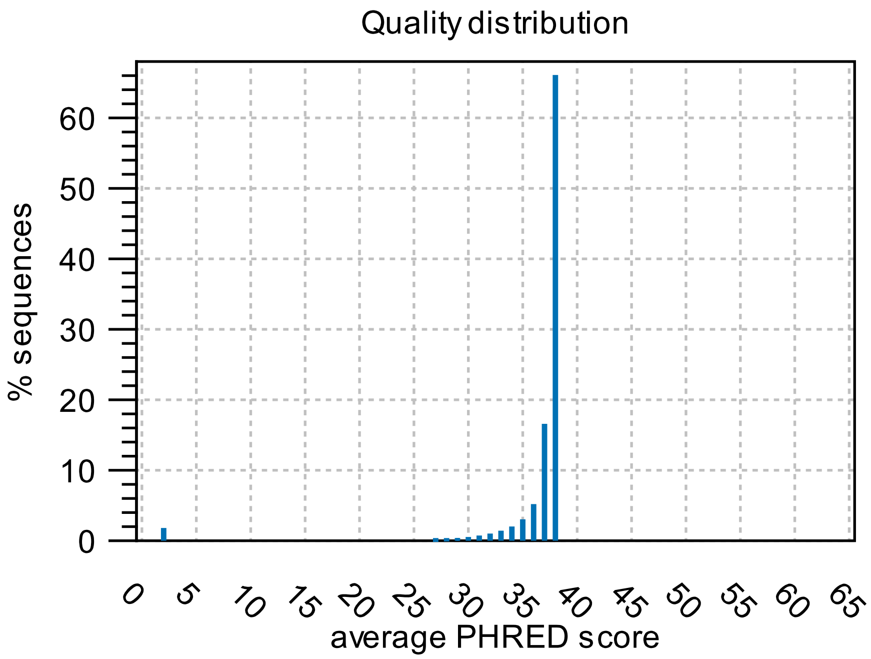

Distribution of average sequence qualitie scores. The quality of a sequence is calculated as the arithmetic mean of its base qualities.
x: PHRED-score
y: number of sequences observed at that qual. score normalized to the total number of sequences

3. Per-base analysis

3.1 Coverage

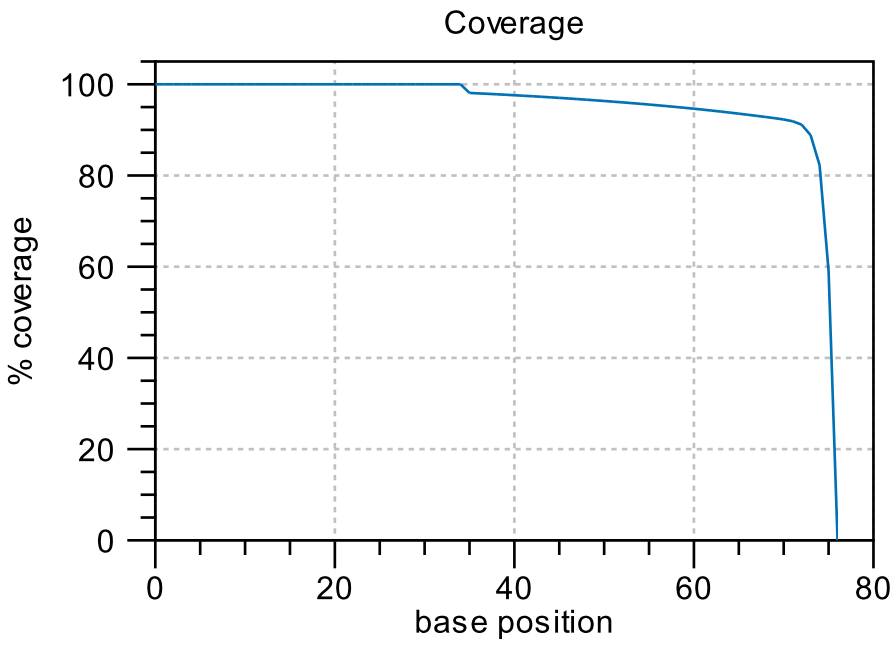

The number of sequences that support (cover) the individual base positions. In cases of untrimmed Illumina or SOLiD reads it will just contain a rectangle.

x: base position

y: number of sequences covering individual base positions normalized to the total number of sequences

3.2 Nucleotide contributions

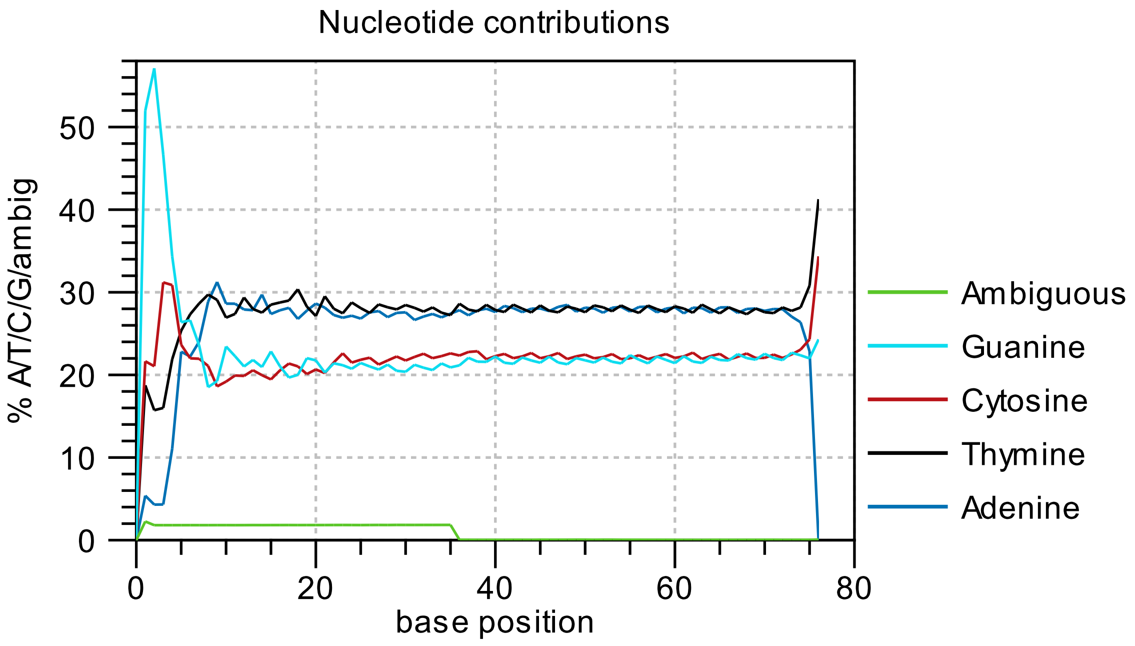

Coverages for the four DNA nucleotides and ambiguous bases.
x: base position
y: number of nucleotides observed per type normalized to the total number of nucleotides observed at that position

3.3 GC-content

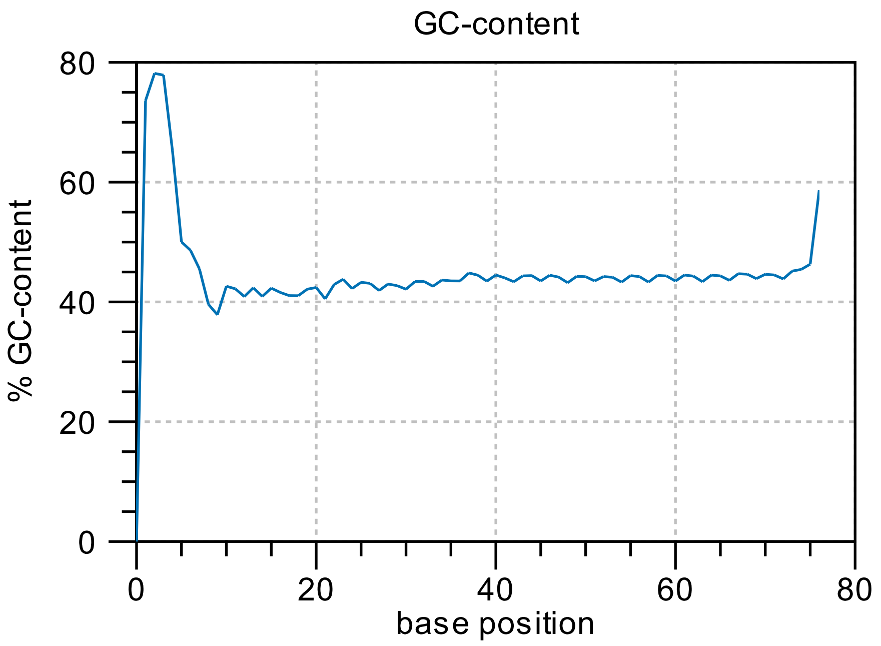

Combined coverage of G- and C-bases.
x: base position
y: number of G- and C-bases observed at current position normalized to the total number of bases observed at that position

3.4 Ambiguous base-content

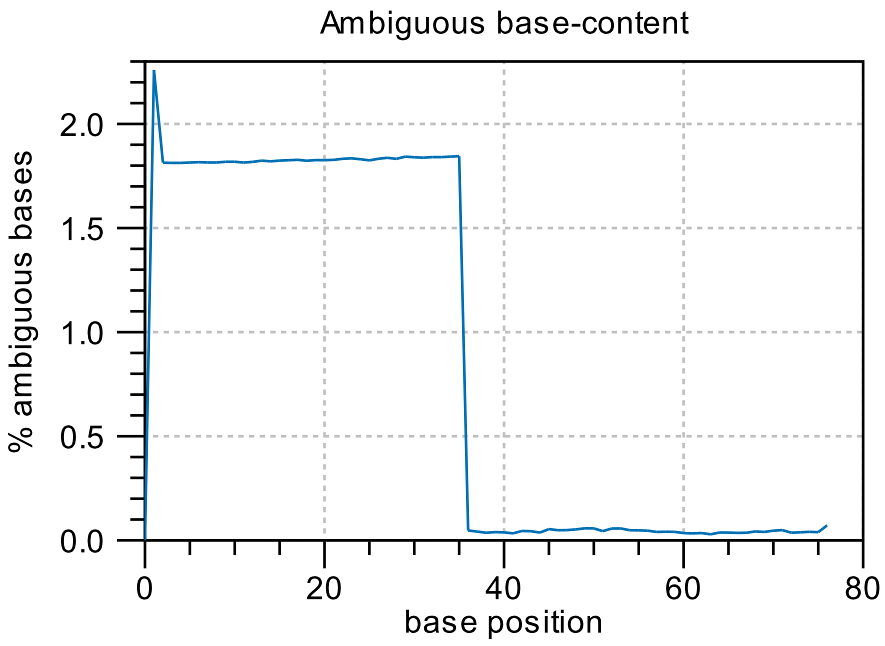

Combined coverage of ambiguous bases.
x: base position
y: number of ambiguous bases observed at current position normalized to the total number of bases observed at that position

3.5 Quality distribution

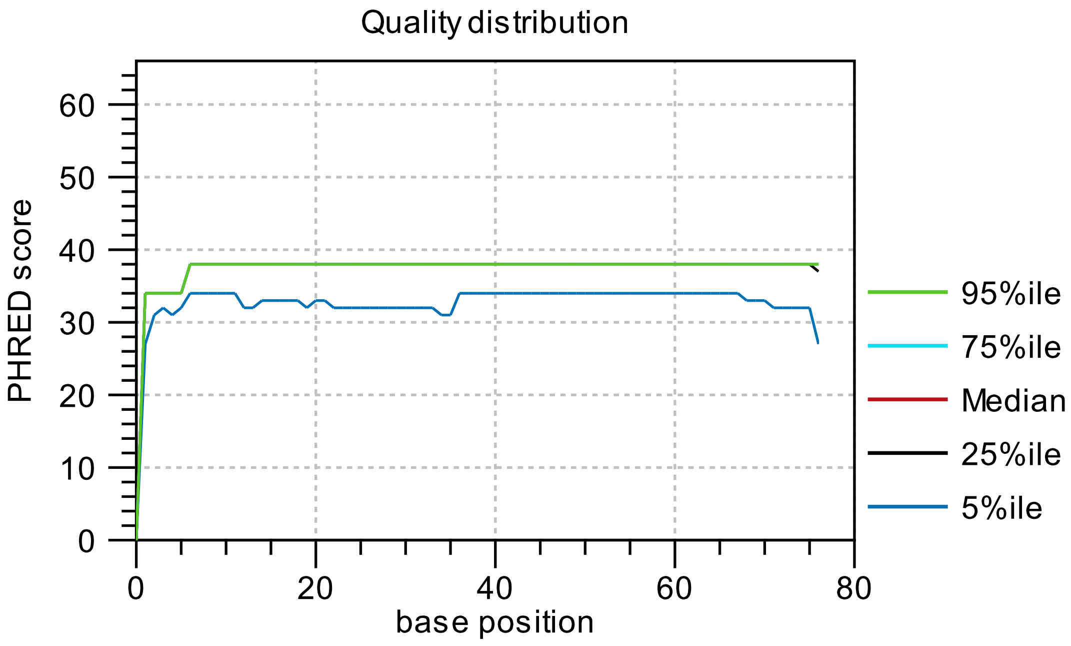

Base-quality distribution along the base positions.
x: base position
y: median & percentiles of quality scores observed at that base position

Sequencing QC Report
Based upon: 21,040,974 sequences in 6 data sets
Generated by: Guerrier
Creation date: Mon Nov 13 14:48:04 CET 2017
Software: CLC Genomics Workbench 9.0.1

Table of contents

1. Summary 3
2. Per-sequence analysis 3
    2.1 Lengths distribution 3
    2.2 GC-content 4
    2.3 Ambiguous base-content 5
    2.4 Quality distribution 6
3. Per-base analysis 6
    3.1 Coverage 7
    3.2 Nucleotide contributions 7
    3.3 GC-content 8
    3.4 Ambiguous base-content 9
    3.5 Quality distribution 9

1. Summary

Creation date:	Mon Nov 13 14:48:04 CET 2017
Generated by:	Guerrier
Software:	CLC Genomics Workbench 9.0.1
Based upon:	6 data sets
H18CTRL2_S5_L001_R1_001 (paired):	4,383,396 sequences in pairs
H18CTRL2_S5_L001_R1_001 (paired)-1:	2,918,988 sequences in pairs
H18C2_S5_L001_R1_001 (paired):	3,153,810 sequences in pairs
H18CTRL2_S5_L001_R1_001 (paired)-4:	3,475,632 sequences in pairs
H18CTRL2_S5_L001_R1_001 (paired)-2:	3,412,036 sequences in pairs
H18CTRL2_S5_L001_R1_001 (paired)-3:	3,697,112 sequences in pairs
Total sequences in data sets	21,040,974 sequences
Total nucleotides in data sets	1,516,743,768 nucleotides

2. Per-sequence analysis

2.1 Lengths distribution

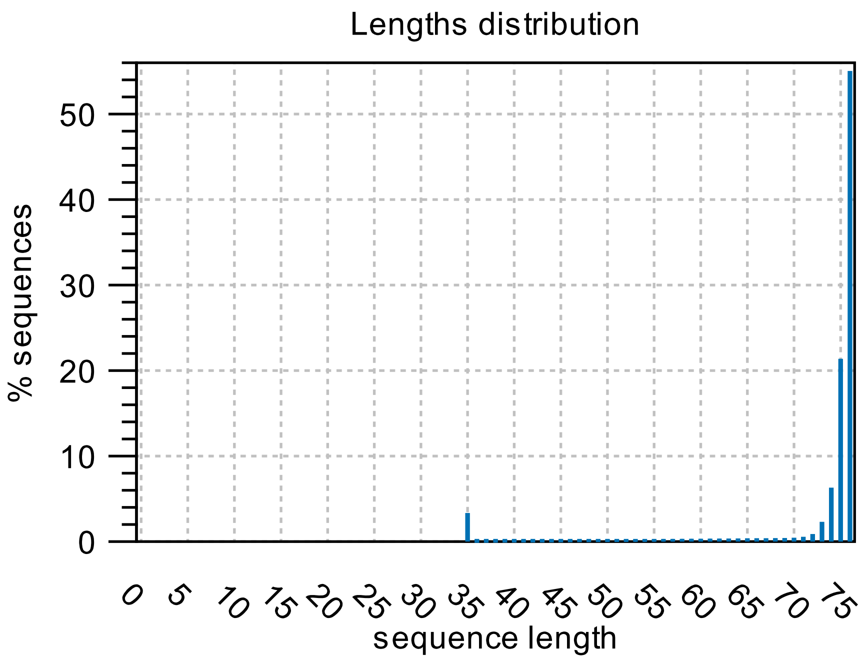

Distribution of sequence lengths. In cases of untrimmed Illumina or SOLiD reads it will just contain a single peak.
x: sequence length in base-pairs

y: number of sequences featuring a particular length normalized to the total number of sequences

2.2 GC-content

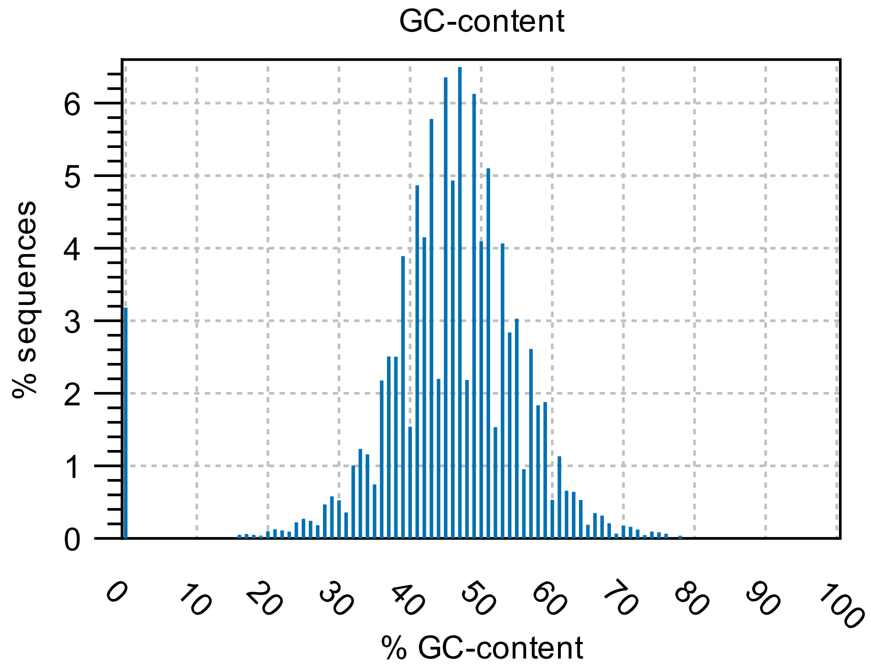

Distribution of GC-contents. The GC-content of a sequence is calculated as the number of GC-bases compared to all bases (including ambiguous bases).
x: relative GC-content of a sequence in percent
y: number of sequences featuring particular GC-percentages normalized to the total number of sequences

2.3 Ambiguous base-content

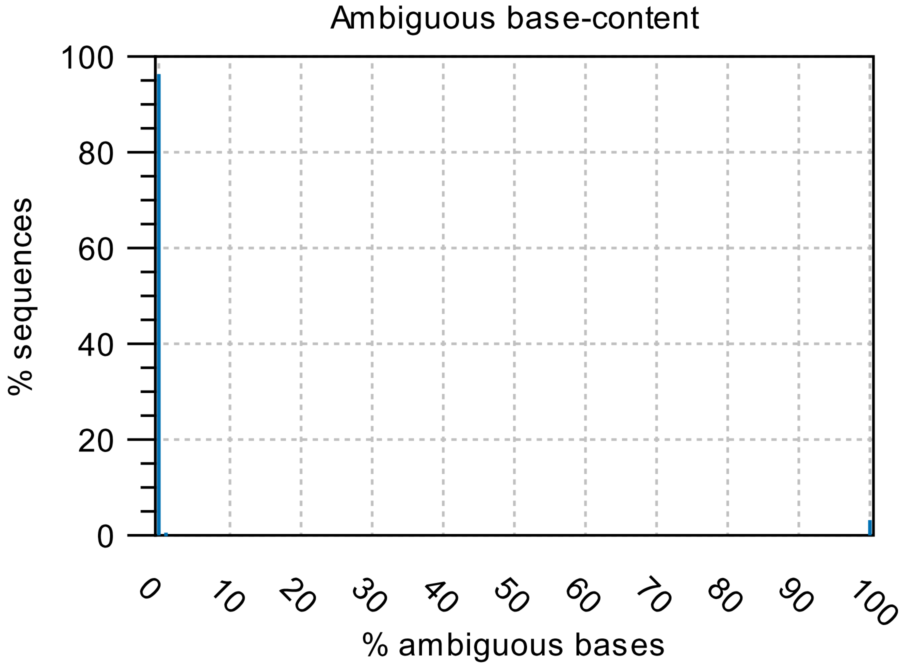

Distribution of N-contents. The N-content of a sequence is calculated as the number of ambiguous bases compared to all bases.
x: relative N-content of a sequence in percent
y: number of sequences featuring particular N-percentages normalized to the total number of sequences

2.4 Quality distribution

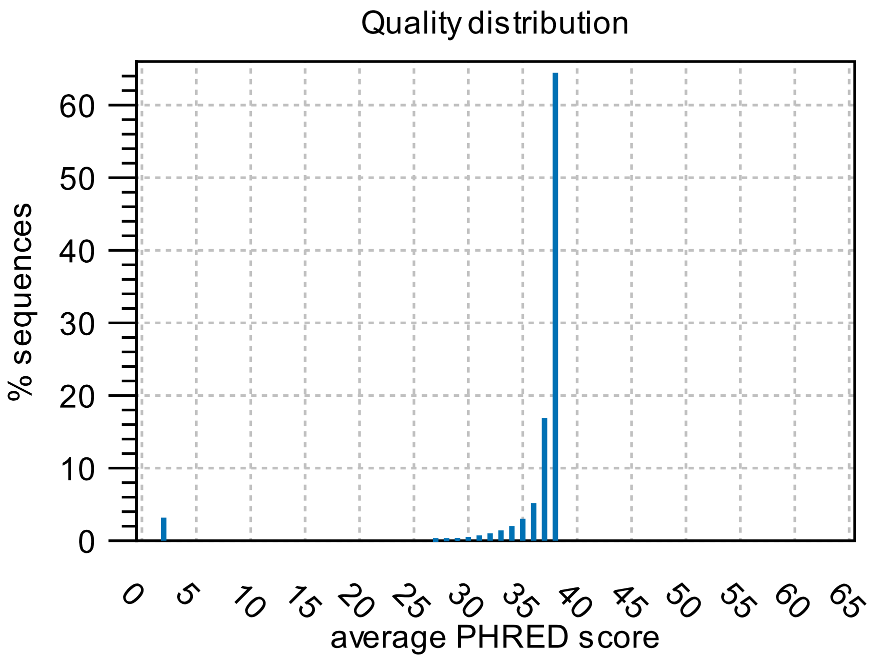

Distribution of average sequence qualitie scores. The quality of a sequence is calculated as the arithmetic mean of its base qualities.
x: PHRED-score
y: number of sequences observed at that qual. score normalized to the total number of sequences

3. Per-base analysis

3.1 Coverage

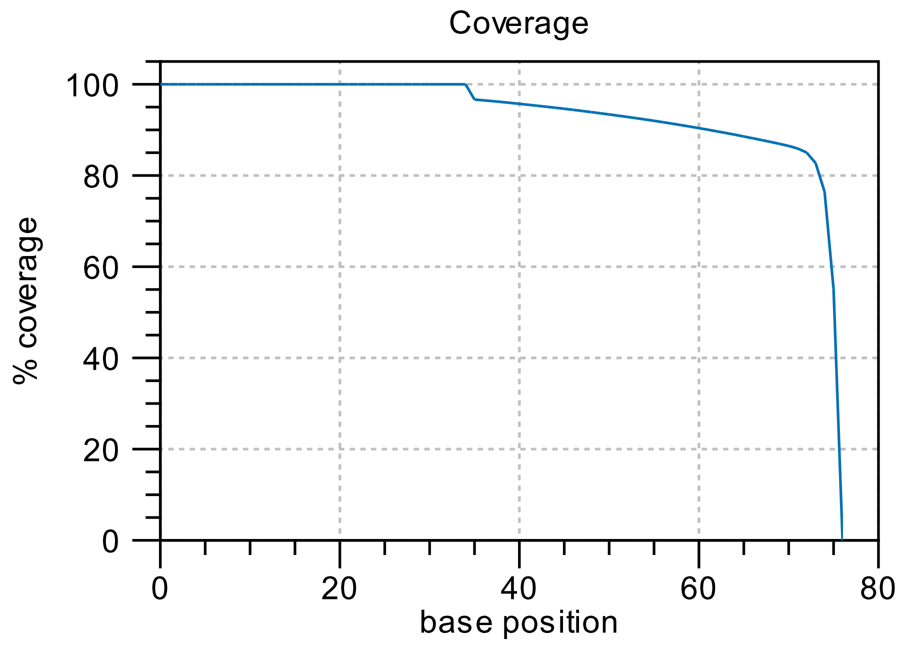

The number of sequences that support (cover) the individual base positions. In cases of untrimmed Illumina or SOLiD reads it will just contain a rectangle.
x: base position
y: number of sequences covering individual base positions normalized to the total number of sequences

3.2 Nucleotide contributions

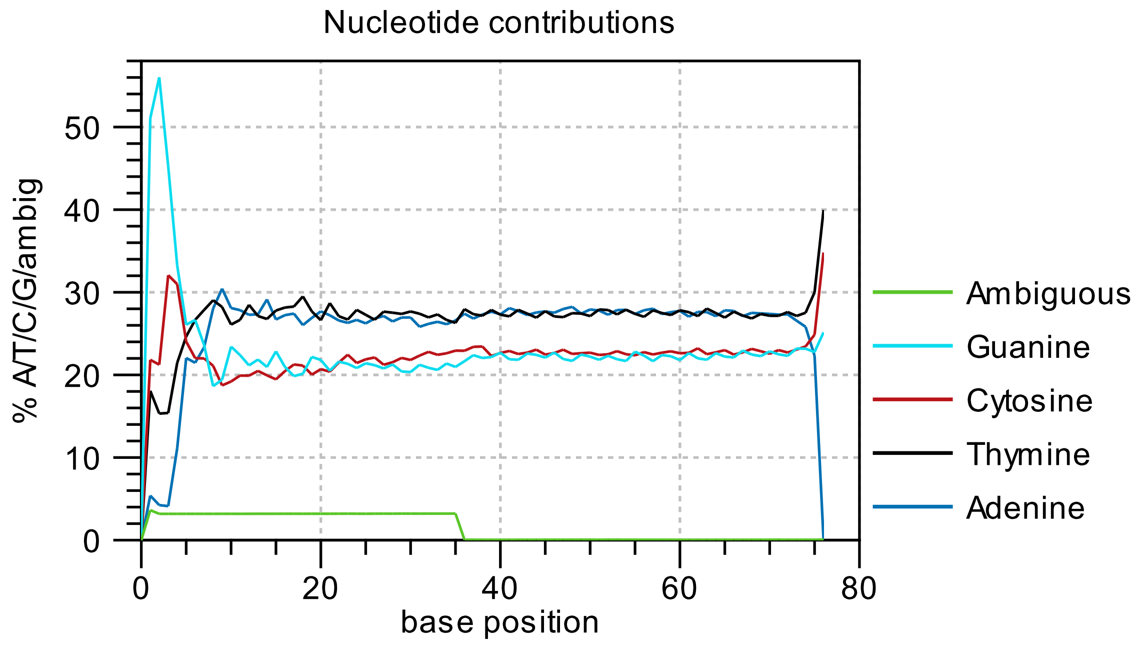

Coverages for the four DNA nucleotides and ambiguous bases.
x: base position
y: number of nucleotides observed per type normalized to the total number of nucleotides observed at that position

3.3 GC-content

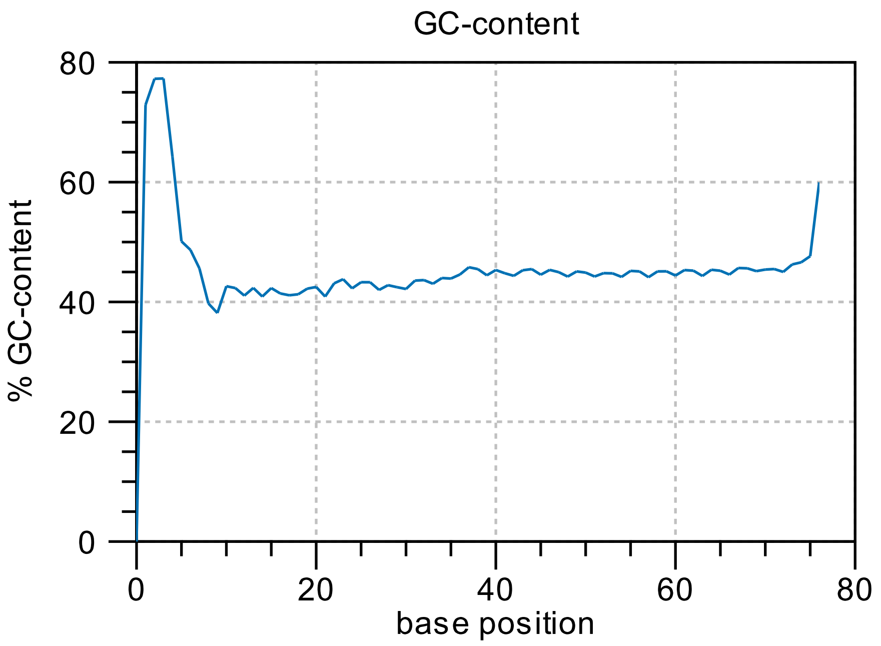

Combined coverage of G- and C-bases.
x: base position
y: number of G- and C-bases observed at current position normalized to the total number of bases observed at that position

3.4 Ambiguous base-content

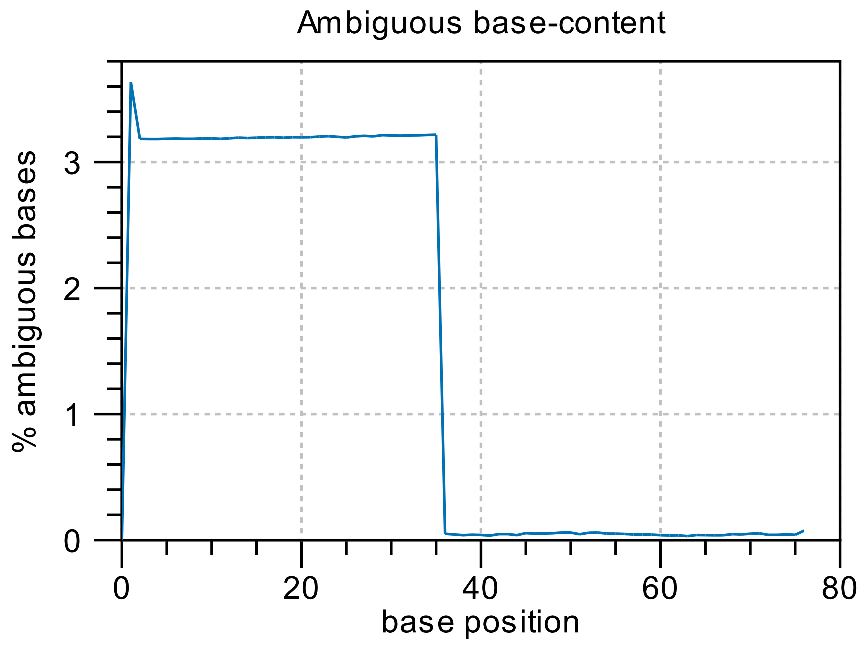

Combined coverage of ambiguous bases.
x: base position
y: number of ambiguous bases observed at current position normalized to the total number of bases observed at that position

3.5 Quality distribution

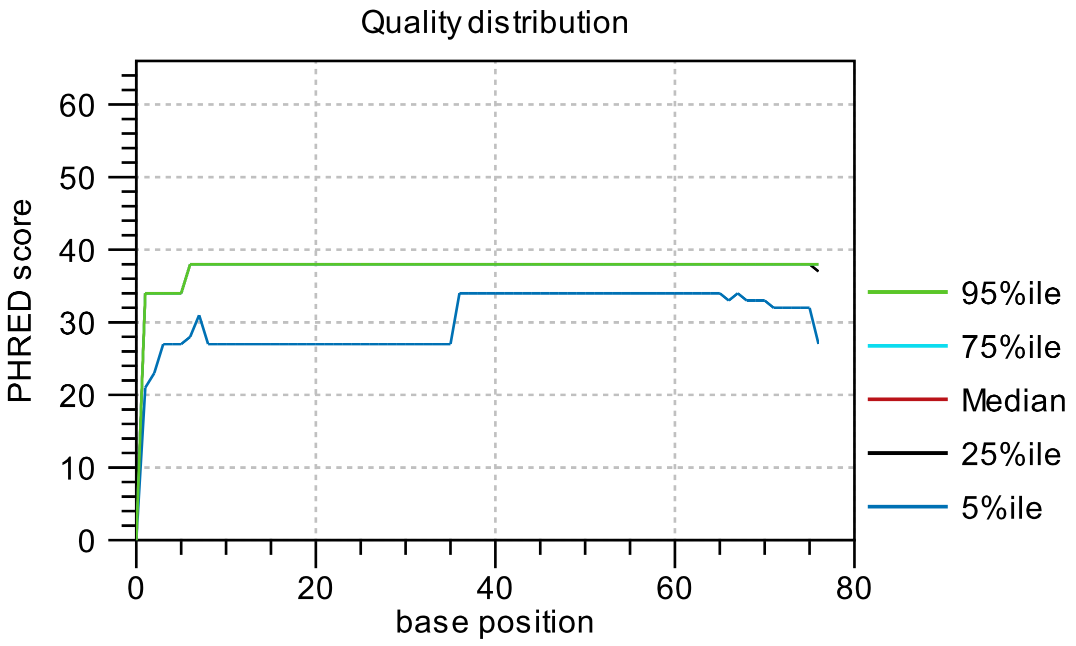

Base-quality distribution along the base positions.
x: base position
y: median & percentiles of quality scores observed at that base position

Sequencing QC Report
Based upon: 27,964,642 sequences in 6 data sets
Generated by: Guerrier
Creation date: Mon Nov 13 14:51:39 CET 2017
Software: CLC Genomics Workbench 9.0.1

Table of contents

1. Summary 3
2. Per-sequence analysis 3
    2.1 Lengths distribution 3
    2.2 GC-content 4
    2.3 Ambiguous base-content 5
    2.4 Quality distribution 6
3. Per-base analysis 6
    3.1 Coverage 7
    3.2 Nucleotide contributions 7
    3.3 GC-content 8
    3.4 Ambiguous base-content 9
    3.5 Quality distribution 9

1. Summary

Creation date:	Mon Nov 13 14:51:39 CET 2017
Generated by:	Guerrier
Software:	CLC Genomics Workbench 9.0.1
Based upon:	6 data sets
H18CTRL3_S6_L001_R1_001 (paired)-1:	5,816,750 sequences in pairs
H18CTRL3_S6_L001_R1_001 (paired)-3:	4,898,820 sequences in pairs
H18CTRL3_S6_L001_R1_001 (paired)-2:	4,540,318 sequences in pairs
H18CTRL3_S6_L001_R1_001 (paired)-4:	4,555,564 sequences in pairs
H18CTRL3_S6_L001_R1_001 (paired):	3,897,362 sequences in pairs
H18C3_S6_L001_R1_001 (paired):	4,255,828 sequences in pairs
Total sequences in data sets	27,964,642 sequences
Total nucleotides in data sets	2,045,004,884 nucleotides

2. Per-sequence analysis

2.1 Lengths distribution

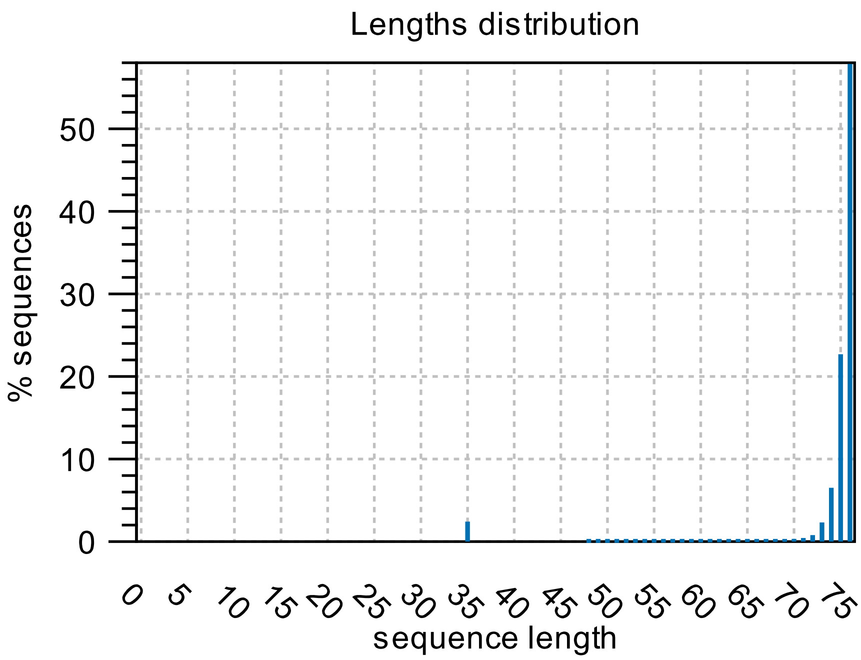

Distribution of sequence lengths. In cases of untrimmed Illumina or SOLiD reads it will just contain a single peak.
x: sequence length in base-pairs

y: number of sequences featuring a particular length normalized to the total number of sequences

2.2 GC-content

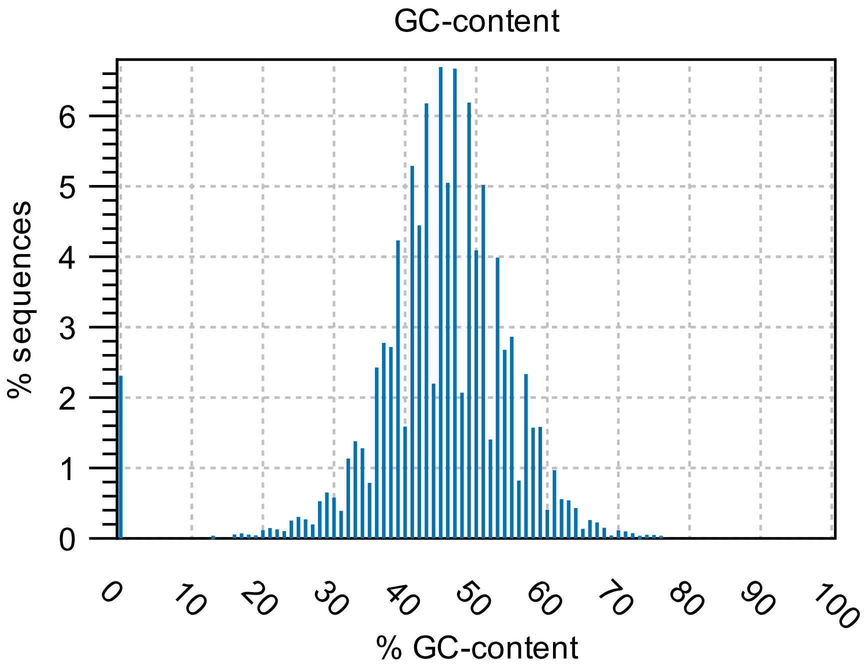

Distribution of GC-contents. The GC-content of a sequence is calculated as the number of GC-bases compared to all bases (including ambiguous bases).
x: relative GC-content of a sequence in percent
y: number of sequences featuring particular GC-percentages normalized to the total number of sequences

2.3 Ambiguous base-content

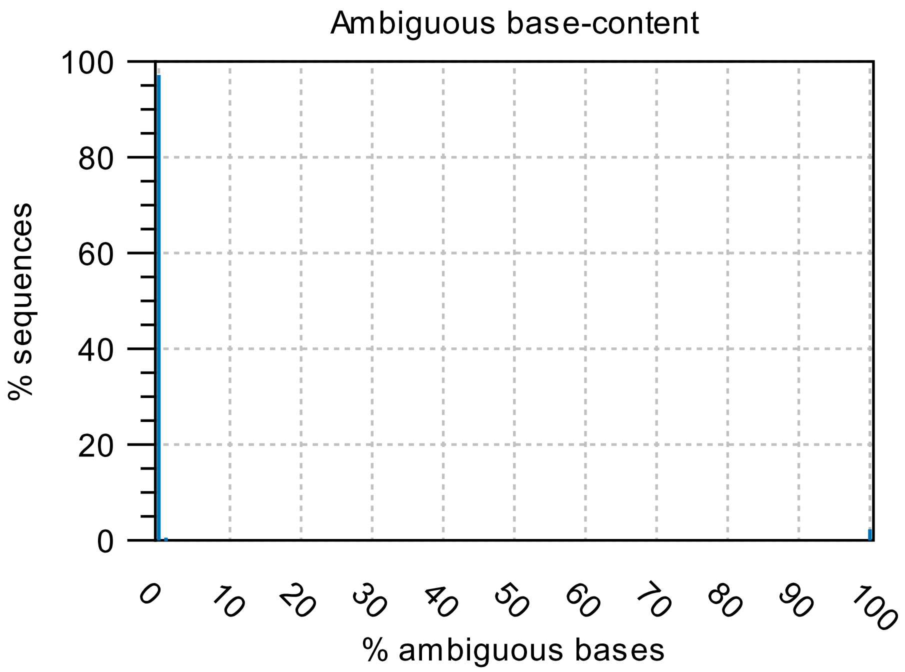

Distribution of N-contents. The N-content of a sequence is calculated as the number of ambiguous bases compared to all bases.
x: relative N-content of a sequence in percent
y: number of sequences featuring particular N-percentages normalized to the total number of sequences

2.4 Quality distribution

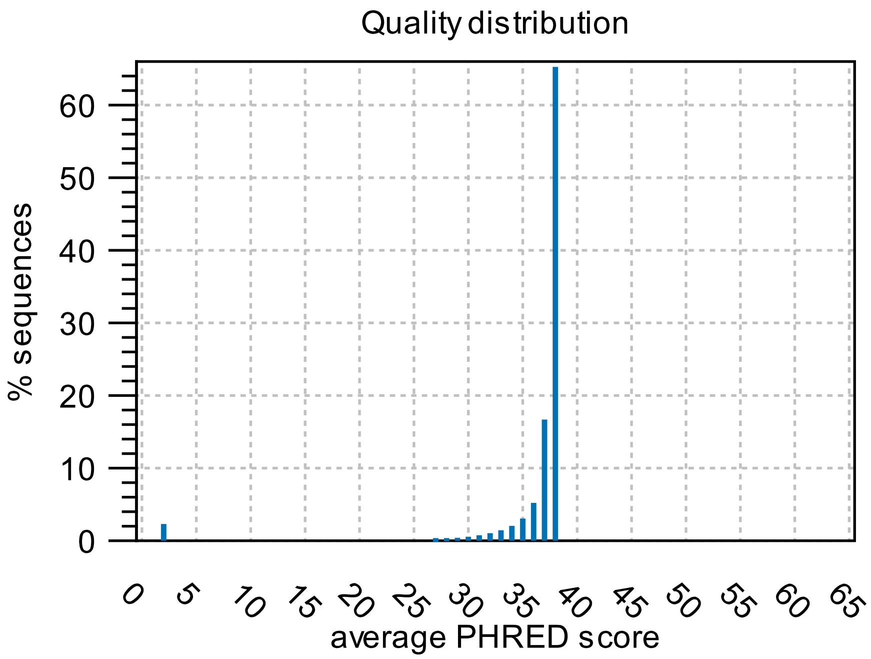

Distribution of average sequence qualitie scores. The quality of a sequence is calculated as the arithmetic mean of its base qualities.
x: PHRED-score
y: number of sequences observed at that qual. score normalized to the total number of sequences

3. Per-base analysis

3.1 Coverage

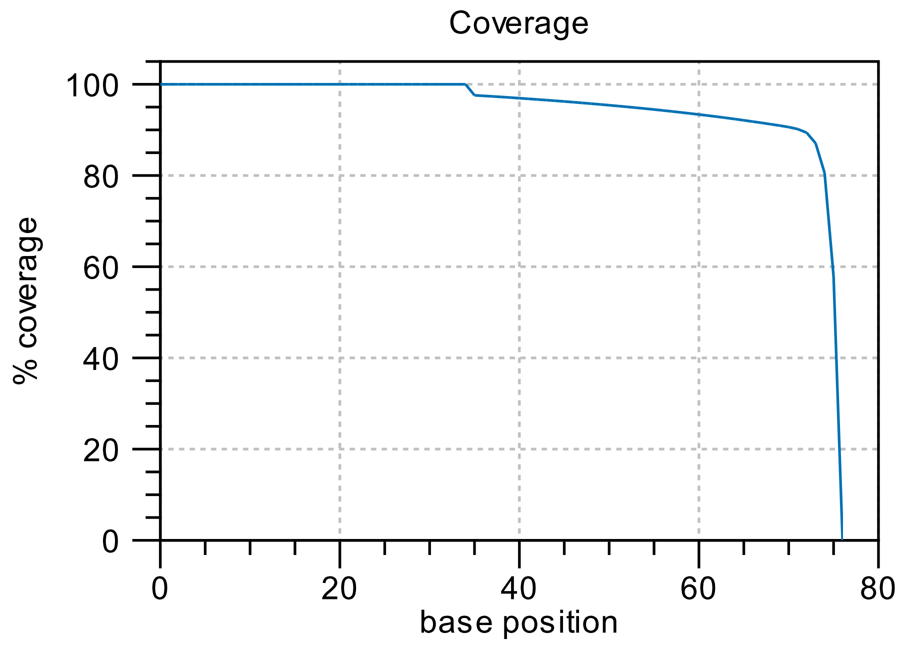

The number of sequences that support (cover) the individual base positions. In cases of untrimmed Illumina or SOLiD reads it will just contain a rectangle.
x: base position
y: number of sequences covering individual base positions normalized to the total number of sequences

3.2 Nucleotide contributions

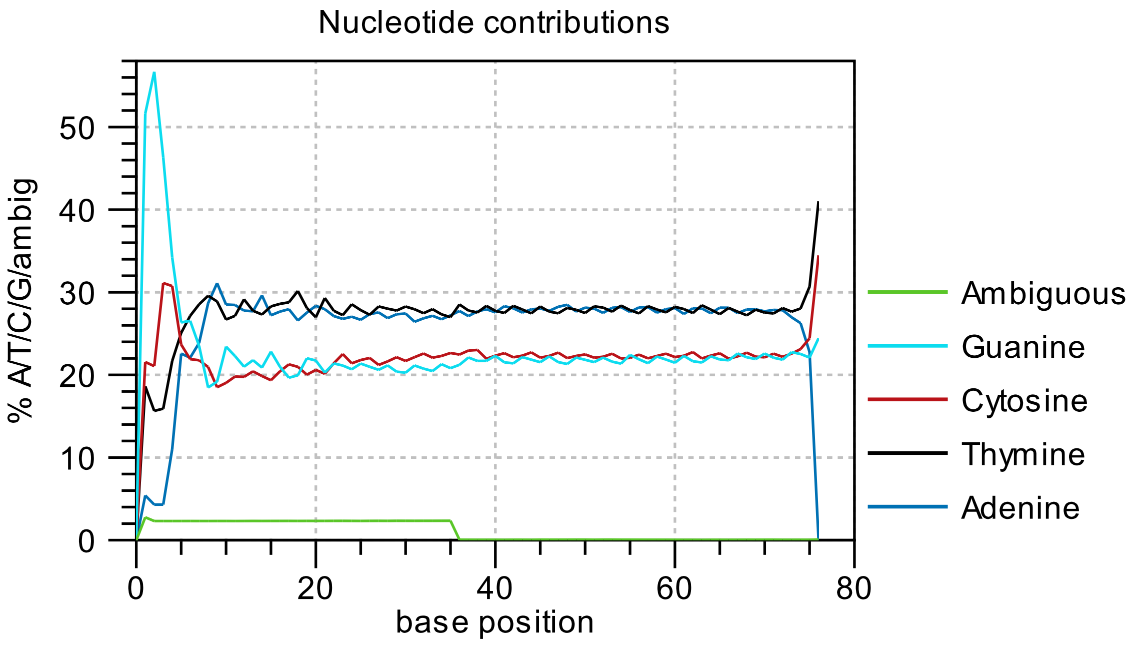

Coverages for the four DNA nucleotides and ambiguous bases.
x: base position
y: number of nucleotides observed per type normalized to the total number of nucleotides observed at that position

3.3 GC-content

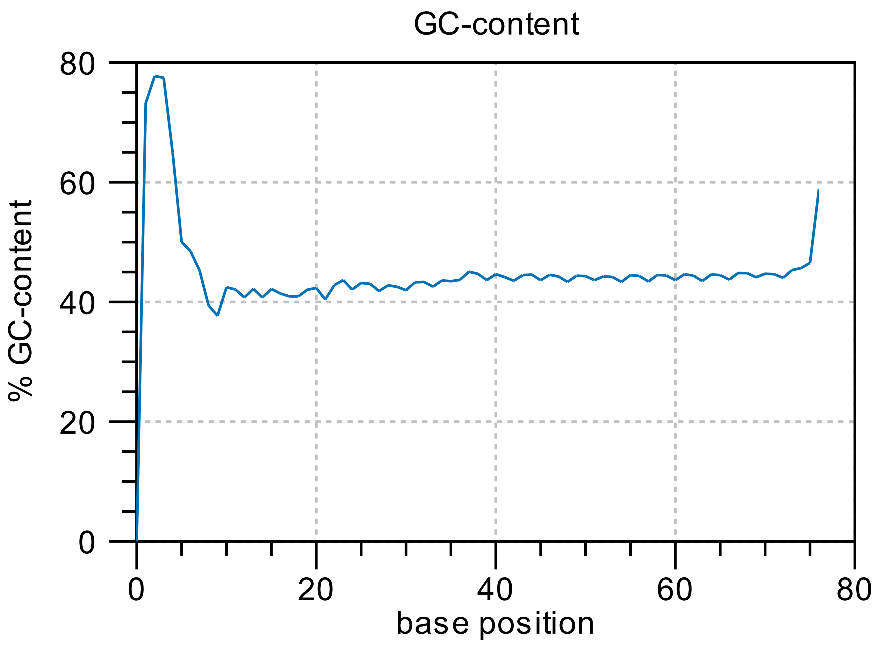

Combined coverage of G- and C-bases.
x: base position
y: number of G- and C-bases observed at current position normalized to the total number of bases observed at that position

3.4 Ambiguous base-content

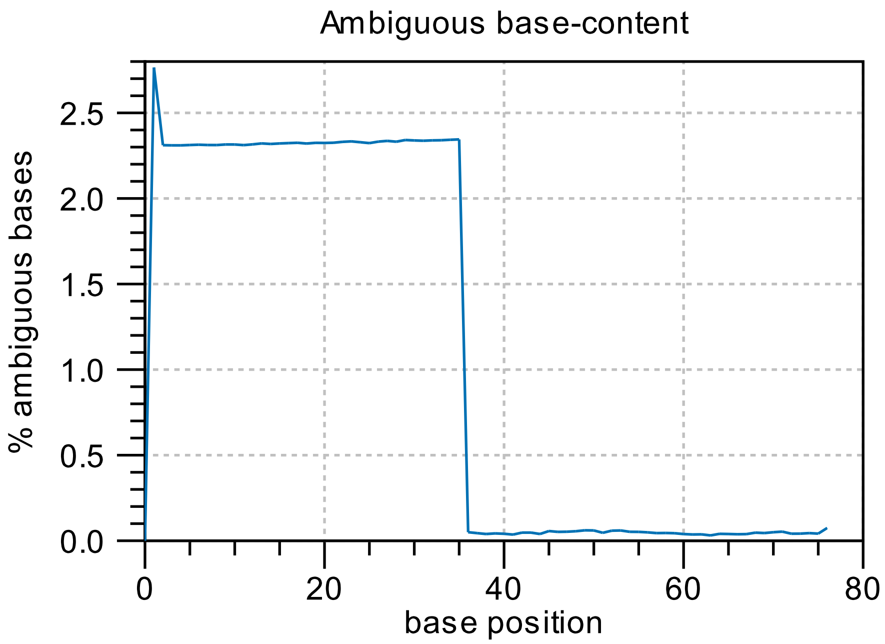

Combined coverage of ambiguous bases.
x: base position
y: number of ambiguous bases observed at current position normalized to the total number of bases observed at that position

3.5 Quality distribution

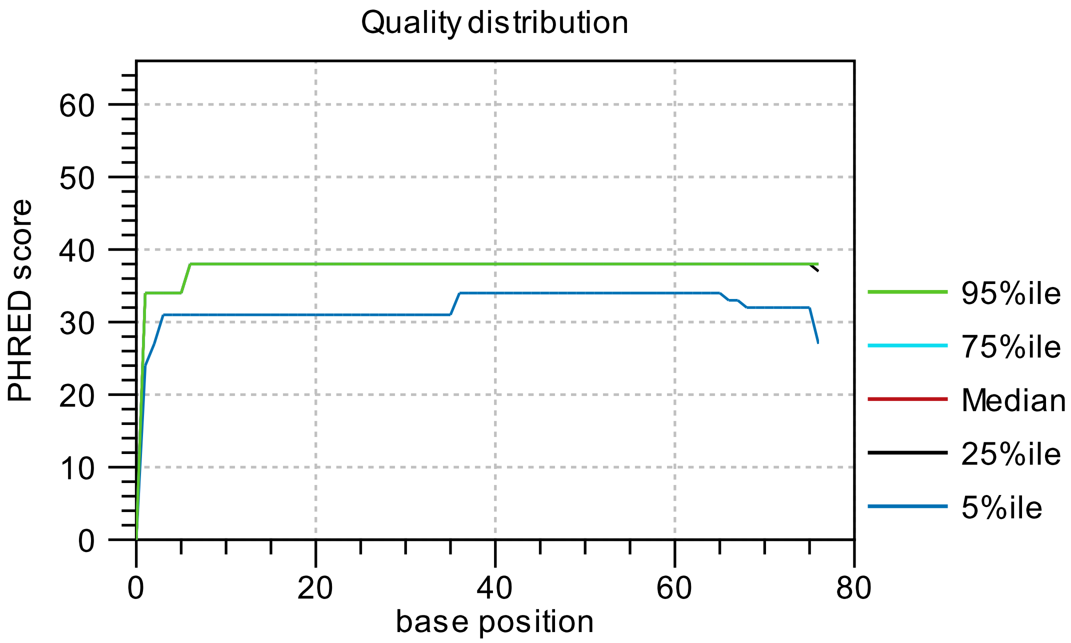

Base-quality distribution along the base positions.
x: base position
y: median & percentiles of quality scores observed at that base position

Sequencing QC Report
Based upon: 18,157,118 sequences in 6 data sets
Generated by: Guerrier
Creation date: Mon Nov 13 16:04:13 CET 2017
Software: CLC Genomics Workbench 9.0.1

Table of contents

1. Summary 3
2. Per-sequence analysis 3
    2.1 Lengths distribution 3
    2.2 GC-content 4
    2.3 Ambiguous base-content 5
    2.4 Quality distribution 6
3. Per-base analysis 6
    3.1 Coverage 7
    3.2 Nucleotide contributions 7
    3.3 GC-content 8
    3.4 Ambiguous base-content 9
    3.5 Quality distribution 9

1. Summary

Creation date:	Mon Nov 13 16:04:13 CET 2017
Generated by:	Guerrier
Software:	CLC Genomics Workbench 9.0.1
Based upon:	6 data sets
H18JA1_S1_L001_R1_001 (paired):	3,748,428 sequences in pairs
H18JA1_S1_L001_R1_001 (paired)-1:	2,504,044 sequences in pairs
H18JA1_S1_L001_R1_001 (paired)-2:	2,929,460 sequences in pairs
H18JA1_S1_L001_R1_001 (paired)-3:	3,199,204 sequences in pairs
H18JA1_S1_L001_R1_001 (paired)-5:	2,794,650 sequences in pairs
H18JA1_S1_L001_R1_001 (paired)-4:	2,981,332 sequences in pairs
Total sequences in data sets	18,157,118 sequences
Total nucleotides in data sets	1,346,002,272 nucleotides

2. Per-sequence analysis

2.1 Lengths distribution

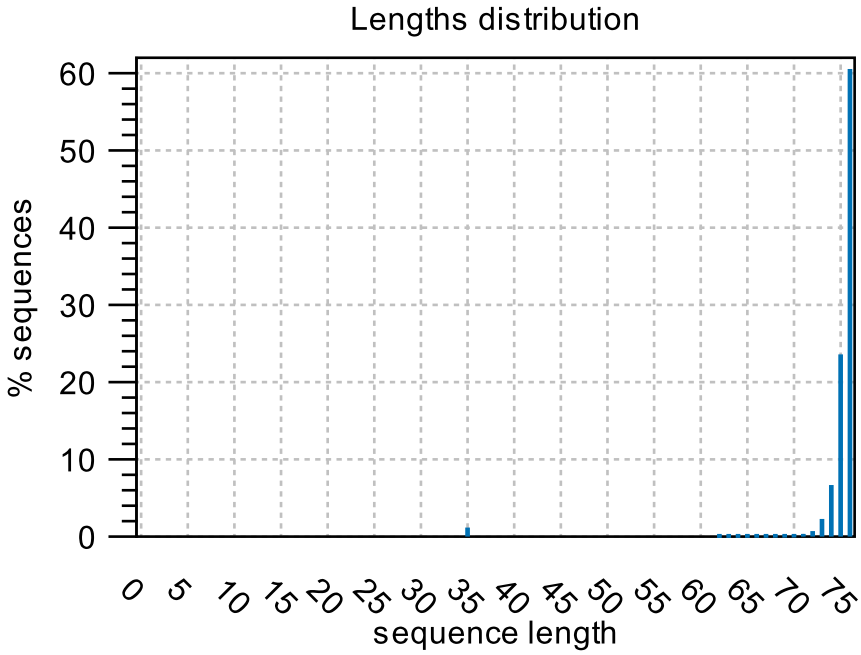

Distribution of sequence lengths. In cases of untrimmed Illumina or SOLiD reads it will just contain a single peak.
x: sequence length in base-pairs

y: number of sequences featuring a particular length normalized to the total number of sequences

2.2 GC-content

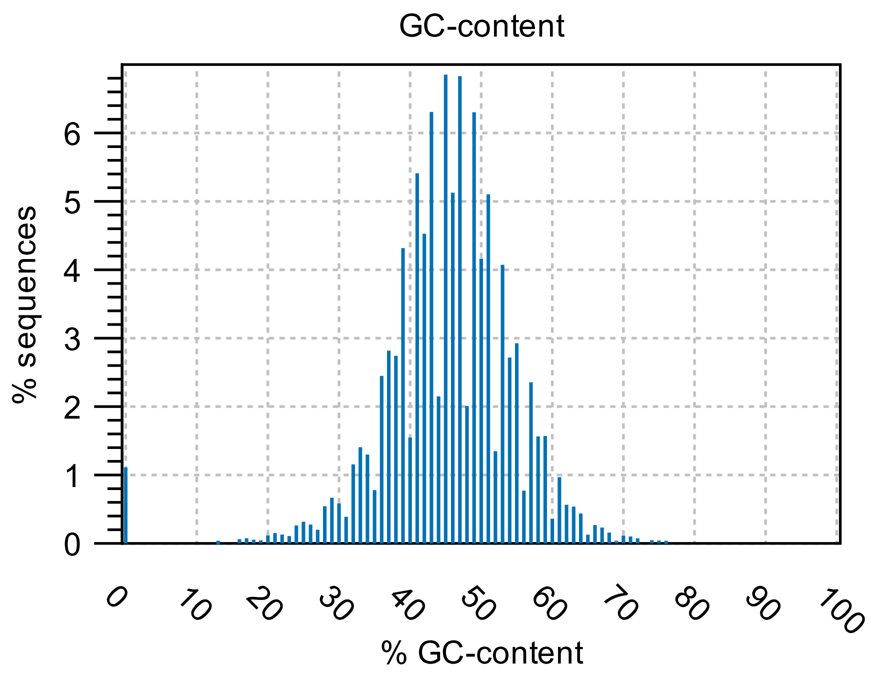

Distribution of GC-contents. The GC-content of a sequence is calculated as the number of GC-bases compared to all bases (including ambiguous bases).
x: relative GC-content of a sequence in percent
y: number of sequences featuring particular GC-percentages normalized to the total number of sequences

2.3 Ambiguous base-content

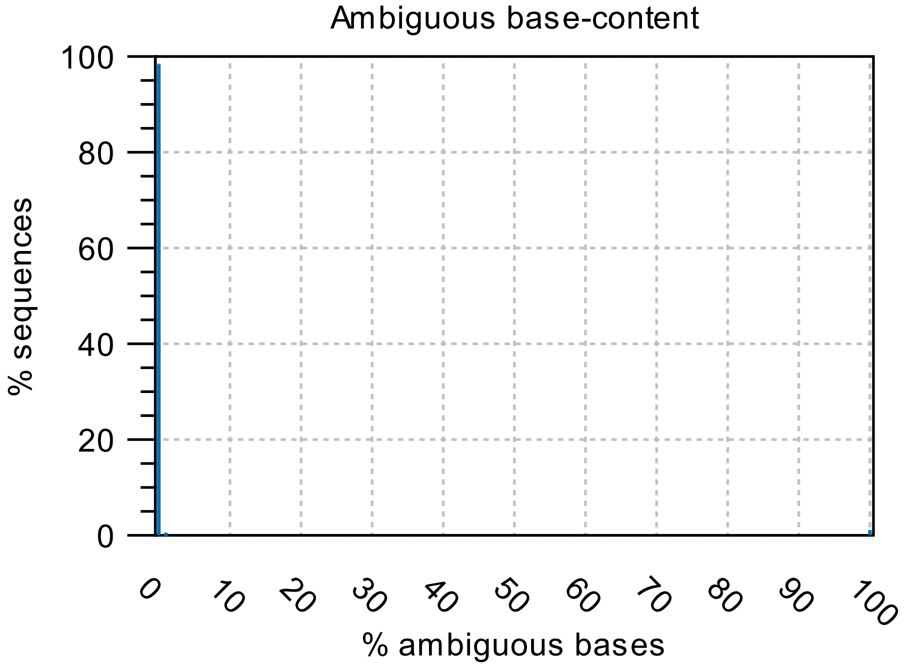

Distribution of N-contents. The N-content of a sequence is calculated as the number of ambiguous bases compared to all bases.
x: relative N-content of a sequence in percent
y: number of sequences featuring particular N-percentages normalized to the total number of sequences

2.4 Quality distribution

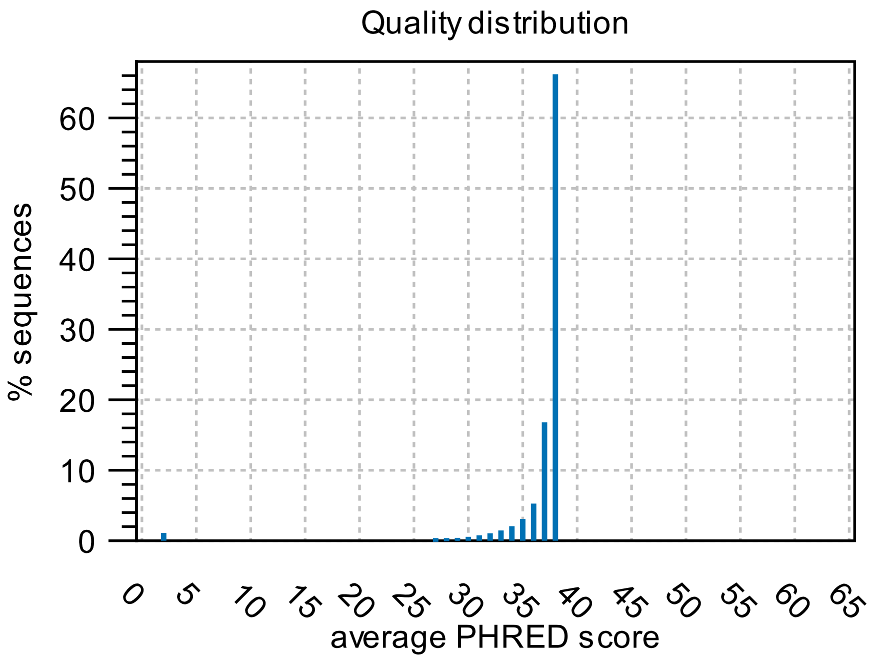

Distribution of average sequence qualitie scores. The quality of a sequence is calculated as the arithmetic mean of its base qualities.
x: PHRED-score
y: number of sequences observed at that qual. score normalized to the total number of sequences

3. Per-base analysis

3.1 Coverage

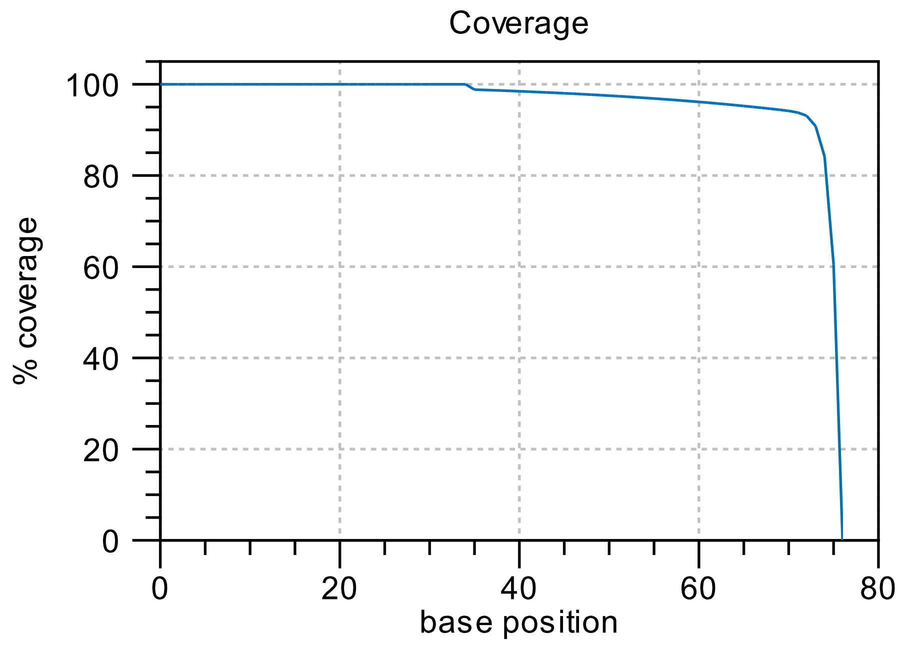

The number of sequences that support (cover) the individual base positions. In cases of untrimmed Illumina or SOLiD reads it will just contain a rectangle.
x: base position
y: number of sequences covering individual base positions normalized to the total number of sequences

3.2 Nucleotide contributions

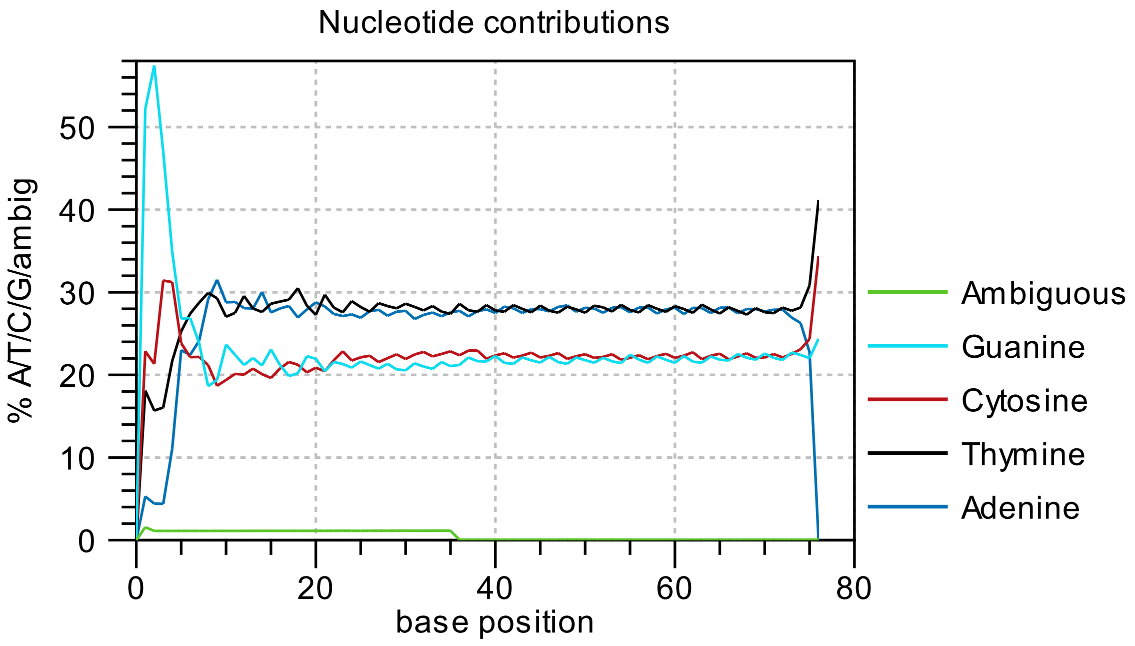

Coverages for the four DNA nucleotides and ambiguous bases.
x: base position
y: number of nucleotides observed per type normalized to the total number of nucleotides observed at that position

3.3 GC-content

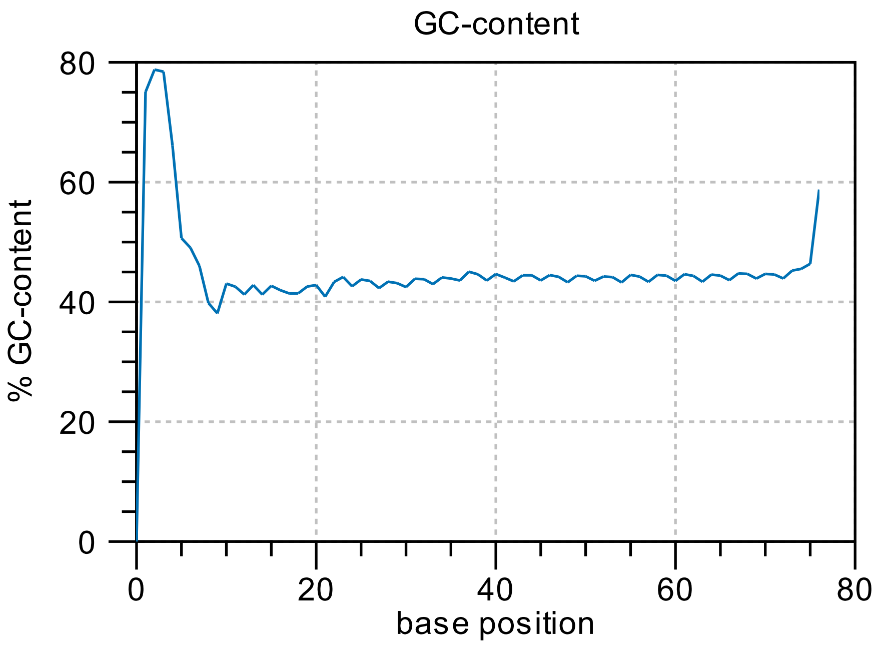

Combined coverage of G- and C-bases.
x: base position
y: number of G- and C-bases observed at current position normalized to the total number of bases observed at that position

3.4 Ambiguous base-content

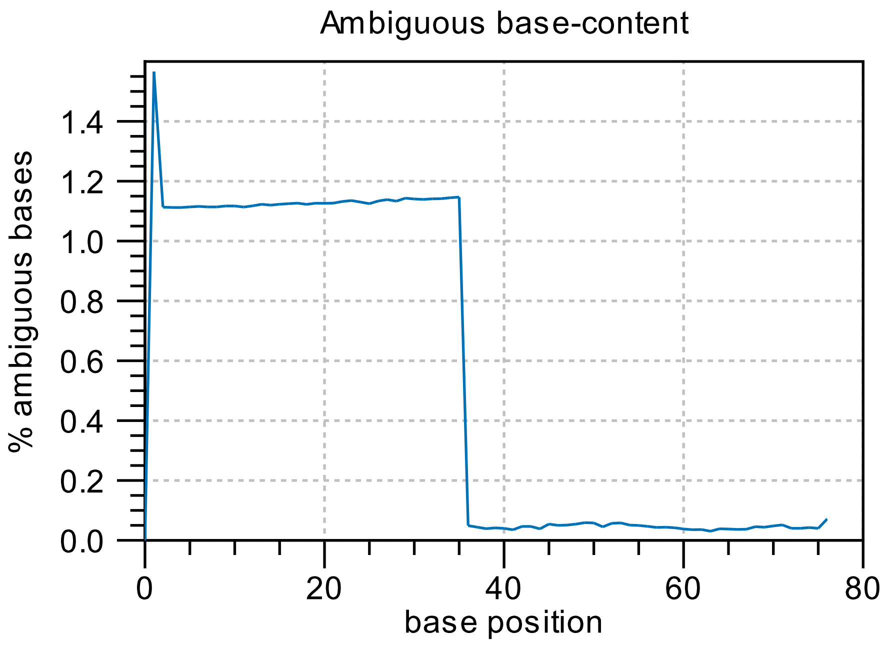

Combined coverage of ambiguous bases.
x: base position
y: number of ambiguous bases observed at current position normalized to the total number of bases observed at that position

3.5 Quality distribution

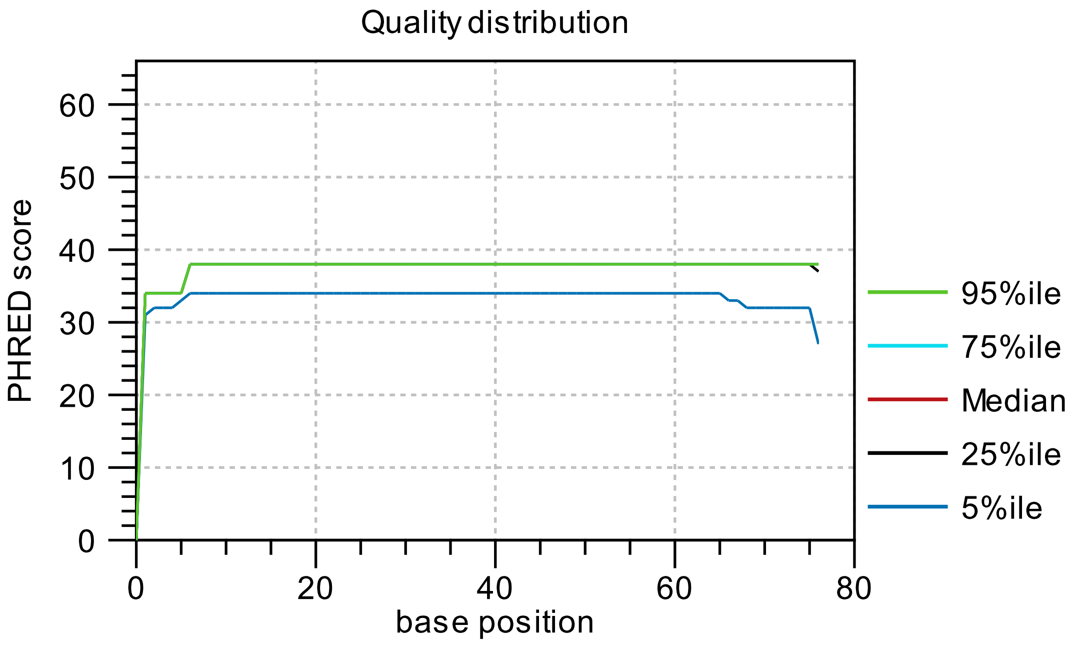

Base-quality distribution along the base positions.
x: base position
y: median & percentiles of quality scores observed at that base position

Sequencing QC Report
Based upon: 25,838,470 sequences in 6 data sets
Generated by: Guerrier
Creation date: Mon Nov 13 15:00:29 CET 2017
Software: CLC Genomics Workbench 9.0.1

Table of contents

1. Summary 3
2. Per-sequence analysis 3
    2.1 Lengths distribution 3
    2.2 GC-content 4
    2.3 Ambiguous base-content 5
    2.4 Quality distribution 6
3. Per-base analysis 6
    3.1 Coverage 7
    3.2 Nucleotide contributions 7
    3.3 GC-content 8
    3.4 Ambiguous base-content 9
    3.5 Quality distribution 9

1. Summary

Creation date:	Mon Nov 13 15:00:29 CET 2017
Generated by:	Guerrier
Software:	CLC Genomics Workbench 9.0.1
Based upon:	6 data sets
H18JA2_S2_L001_R1_001 (paired):	5,337,254 sequences in pairs
H18JA2_S2_L001_R1_001 (paired)-1:	3,556,566 sequences in pairs
H18JA2_S2_L001_R1_001 (paired)-2:	4,147,890 sequences in pairs
H18JA2_S2_L001_R1_001 (paired)-3:	4,278,944 sequences in pairs
H18JA2_S2_L001_R1_001 (paired)-4:	4,561,142 sequences in pairs
H18JA2_S2_L001_R1_001 (paired)-5:	3,956,674 sequences in pairs
Total sequences in data sets	25,838,470 sequences
Total nucleotides in data sets	1,870,926,297 nucleotides

2. Per-sequence analysis

2.1 Lengths distribution

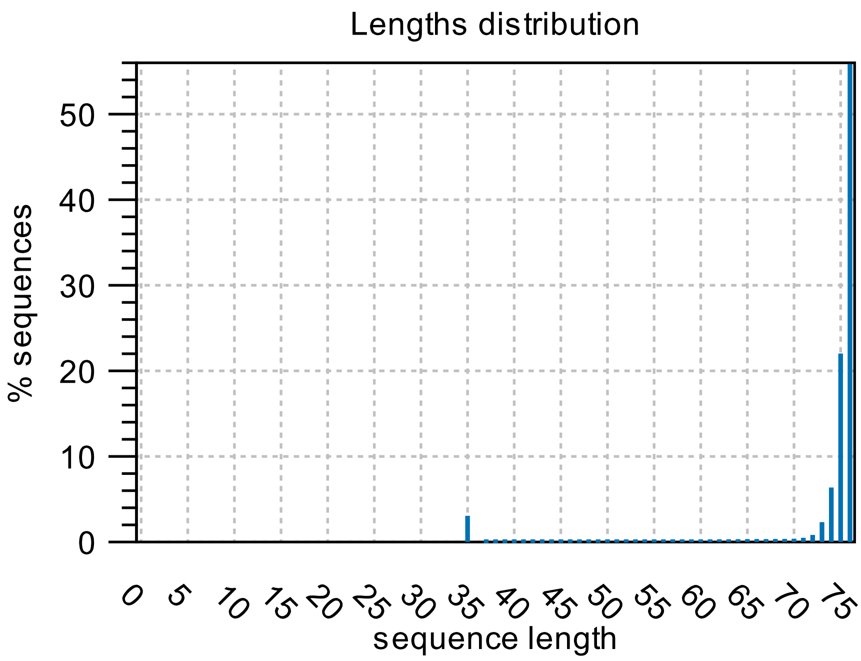

Distribution of sequence lengths. In cases of untrimmed Illumina or SOLiD reads it will just contain a single peak.
x: sequence length in base-pairs

y: number of sequences featuring a particular length normalized to the total number of sequences

2.2 GC-content

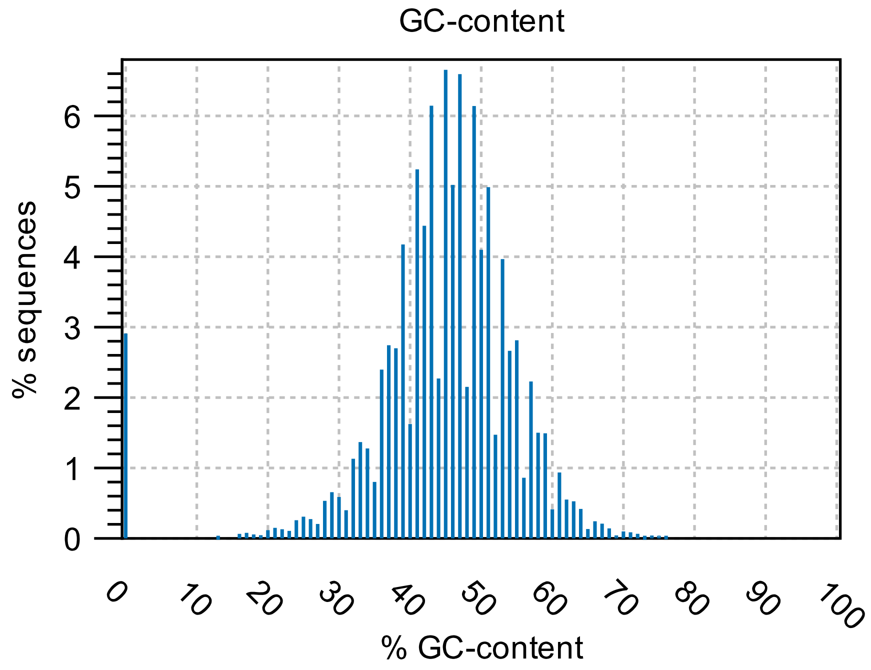

Distribution of GC-contents. The GC-content of a sequence is calculated as the number of GC-bases compared to all bases (including ambiguous bases).
x: relative GC-content of a sequence in percent
y: number of sequences featuring particular GC-percentages normalized to the total number of sequences

2.3 Ambiguous base-content

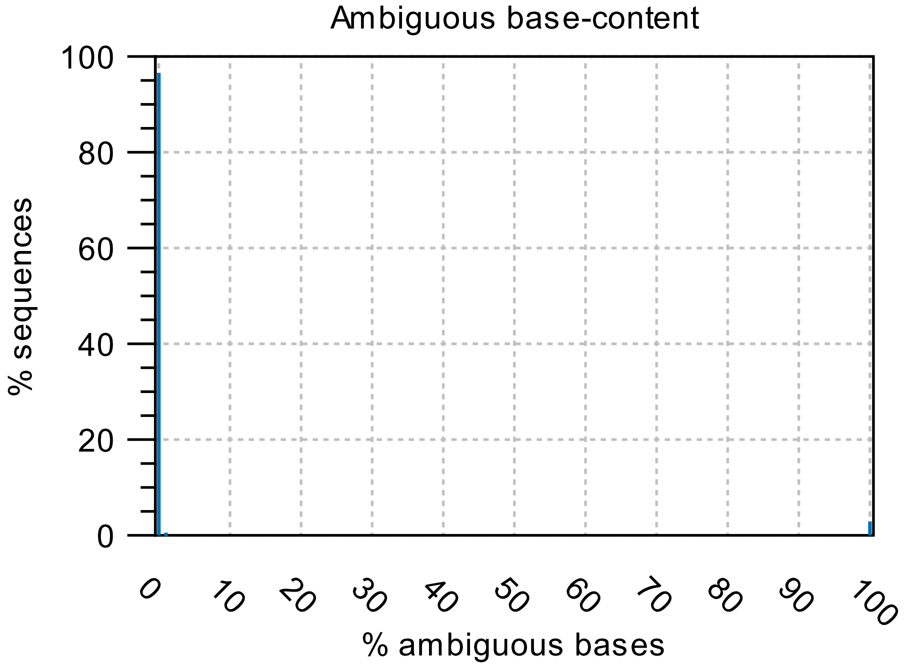

Distribution of N-contents. The N-content of a sequence is calculated as the number of ambiguous bases compared to all bases.
x: relative N-content of a sequence in percent
y: number of sequences featuring particular N-percentages normalized to the total number of sequences

2.4 Quality distribution

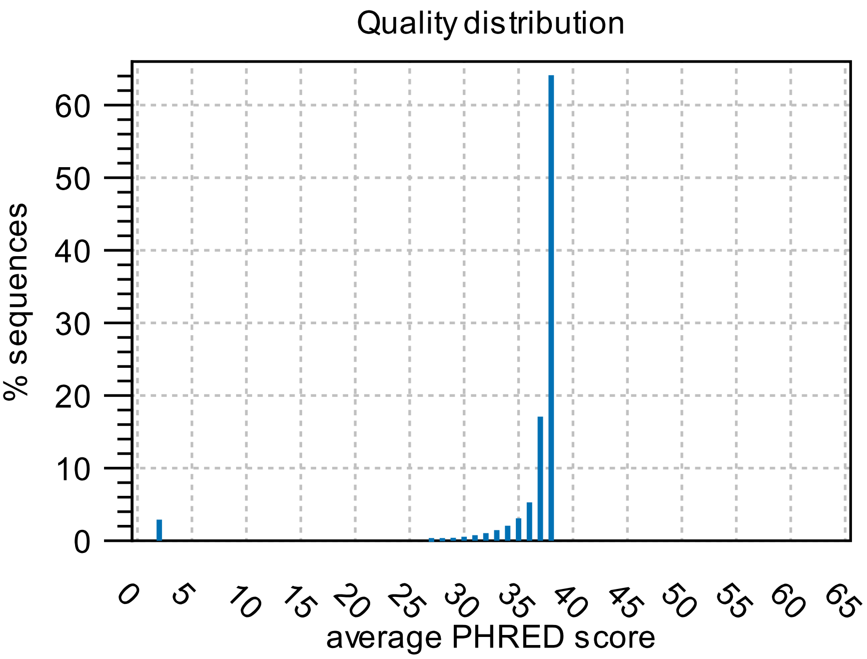

Distribution of average sequence qualitie scores. The quality of a sequence is calculated as the arithmetic mean of its base qualities.
x: PHRED-score
y: number of sequences observed at that qual. score normalized to the total number of sequences

3. Per-base analysis

3.1 Coverage

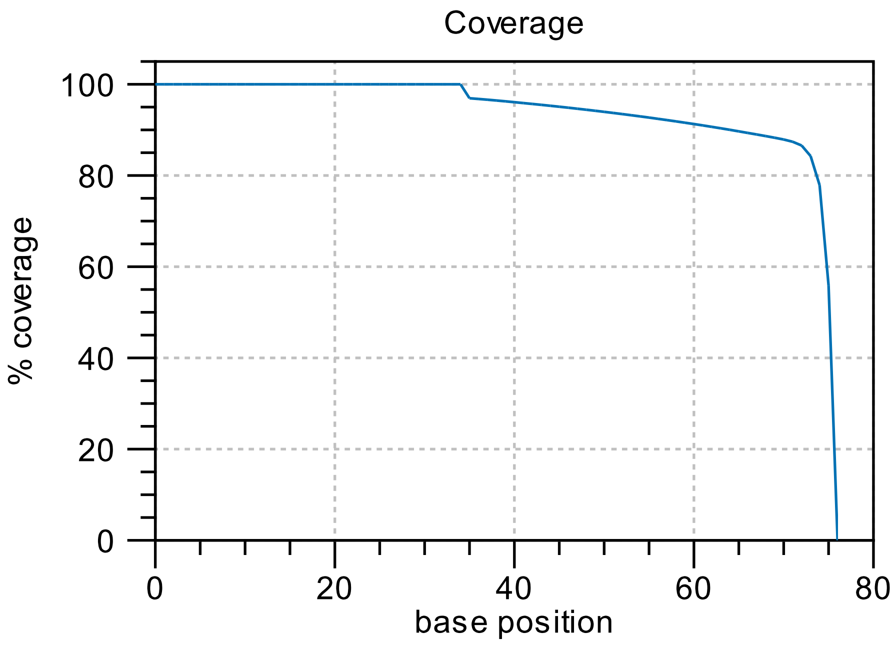

The number of sequences that support (cover) the individual base positions. In cases of untrimmed Illumina or SOLiD reads it will just contain a rectangle.
x: base position
y: number of sequences covering individual base positions normalized to the total number of sequences

3.2 Nucleotide contributions

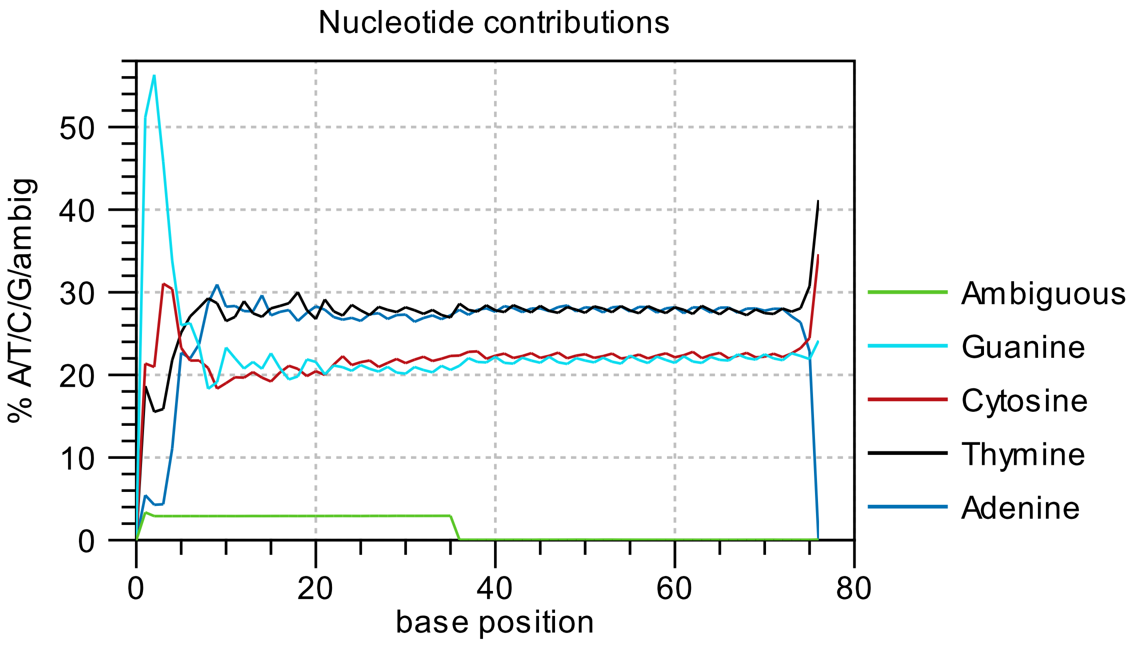

Coverages for the four DNA nucleotides and ambiguous bases.
x: base position
y: number of nucleotides observed per type normalized to the total number of nucleotides observed at that position

3.3 GC-content

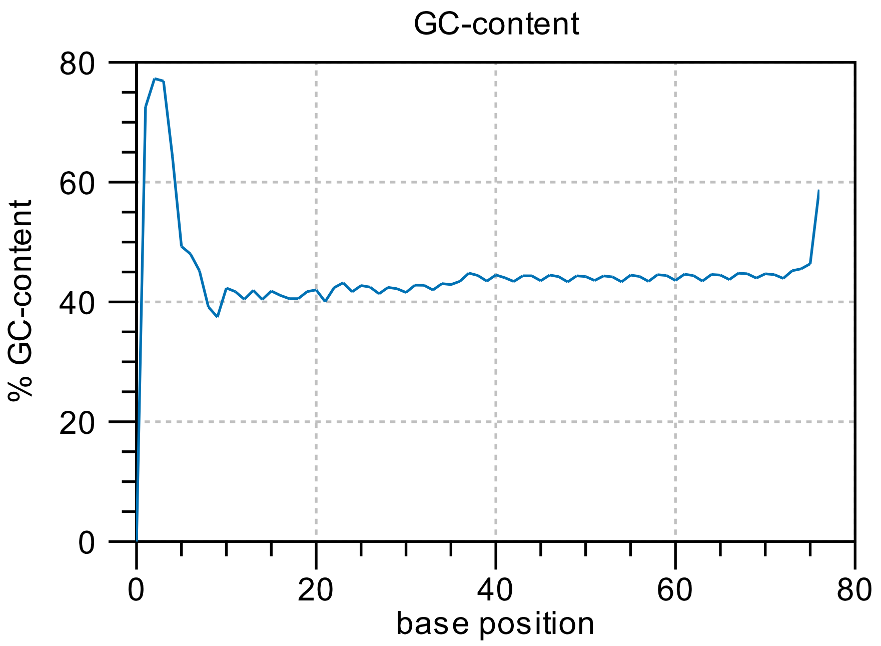

Combined coverage of G- and C-bases.
x: base position
y: number of G- and C-bases observed at current position normalized to the total number of bases observed at that position

3.4 Ambiguous base-content

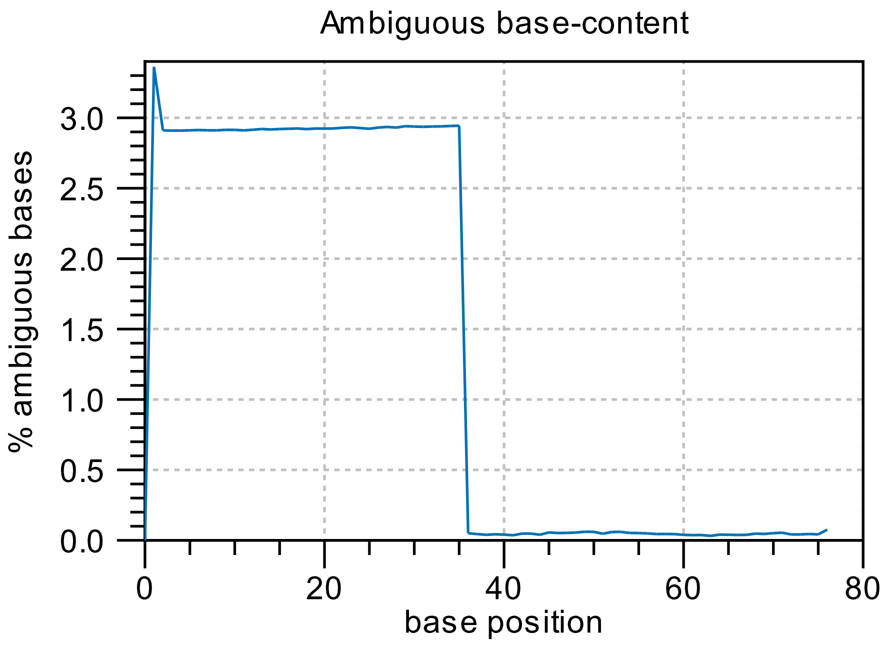

Combined coverage of ambiguous bases.
x: base position
y: number of ambiguous bases observed at current position normalized to the total number of bases observed at that position

3.5 Quality distribution

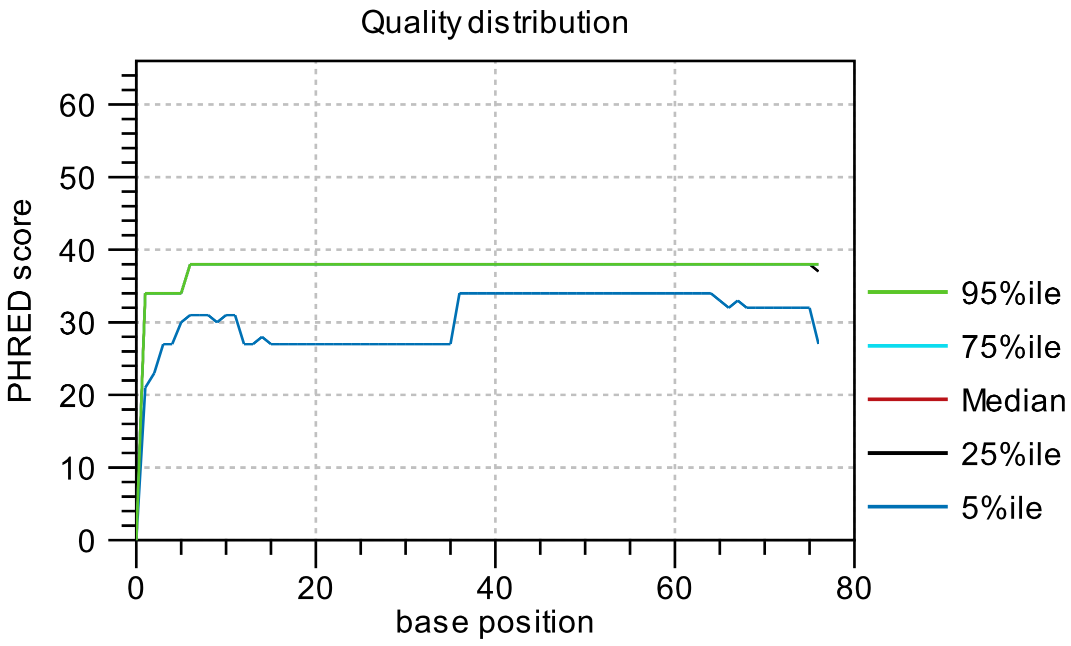

Base-quality distribution along the base positions.
x: base position
y: median & percentiles of quality scores observed at that base position

Sequencing QC Report
Based upon: 27,727,368 sequences in 6 data sets
Generated by: Guerrier
Creation date: Mon Nov 13 15:03:58 CET 2017
Software: CLC Genomics Workbench 9.0.1

Table of contents

1. Summary 3
2. Per-sequence analysis 3
    2.1 Lengths distribution 3
    2.2 GC-content 4
    2.3 Ambiguous base-content 5
    2.4 Quality distribution 6
3. Per-base analysis 6
    3.1 Coverage 7
    3.2 Nucleotide contributions 7
    3.3 GC-content 8
    3.4 Ambiguous base-content 9
    3.5 Quality distribution 9

1. Summary

Creation date:	Mon Nov 13 15:03:58 CET 2017
Generated by:	Guerrier
Software:	CLC Genomics Workbench 9.0.1
Based upon:	6 data sets
H18JA3_S3_L001_R1_001 (paired):	1,963,708 sequences in pairs
H18JA3_S3_L001_R1_001 (paired)-1:	4,503,666 sequences in pairs
H18JA3_S3_L001_R1_001 (paired)-4:	5,264,968 sequences in pairs
H18JA3_S3_L001_R1_001 (paired)-5:	5,215,414 sequences in pairs
H18JA3_S3_L001_R1_001 (paired)-3:	5,516,030 sequences in pairs
H18JA3_S3_L001_R1_001 (paired)-2:	5,263,582 sequences in pairs
Total sequences in data sets	27,727,368 sequences
Total nucleotides in data sets	2,027,696,176 nucleotides

2. Per-sequence analysis

2.1 Lengths distribution

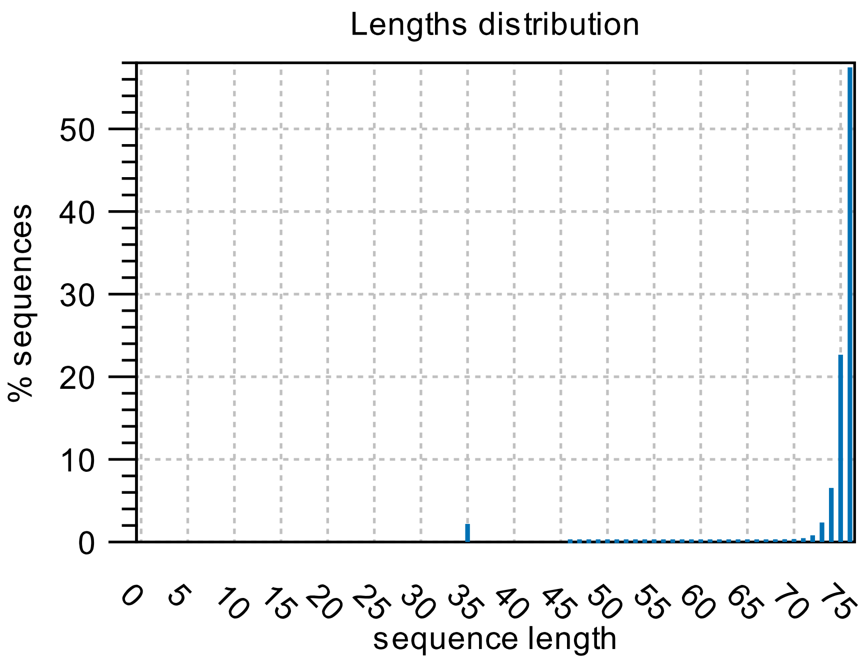

Distribution of sequence lengths. In cases of untrimmed Illumina or SOLiD reads it will just contain a single peak.
x: sequence length in base-pairs

y: number of sequences featuring a particular length normalized to the total number of sequences

2.2 GC-content

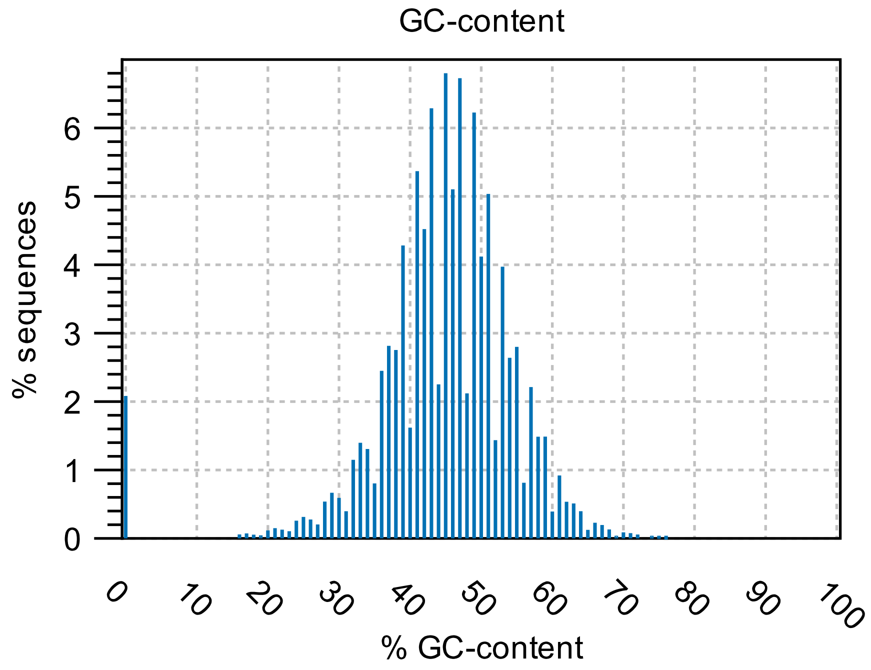

Distribution of GC-contents. The GC-content of a sequence is calculated as the number of GC-bases compared to all bases (including ambiguous bases).
x: relative GC-content of a sequence in percent
y: number of sequences featuring particular GC-percentages normalized to the total number of sequences

2.3 Ambiguous base-content

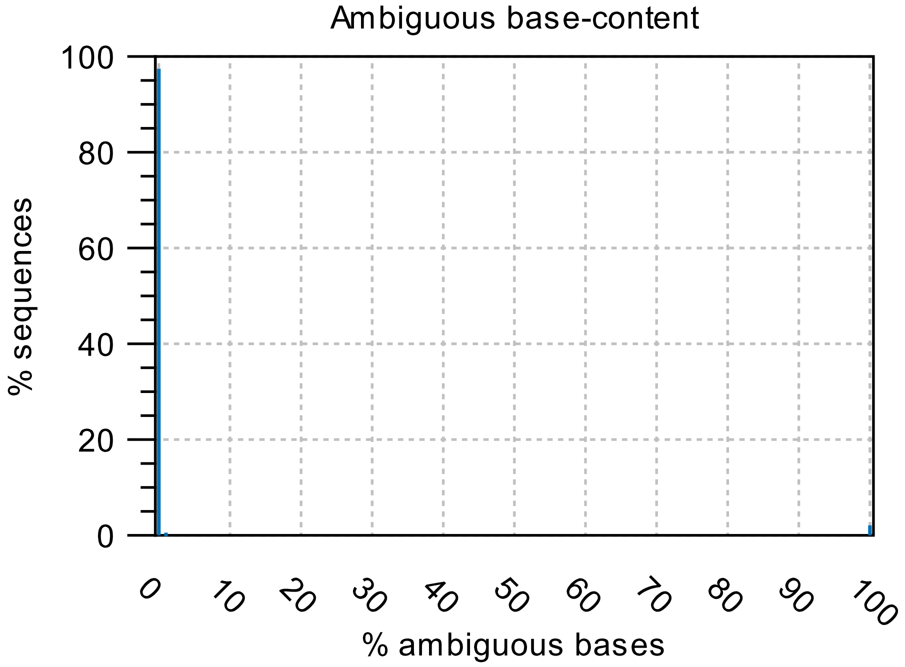

Distribution of N-contents. The N-content of a sequence is calculated as the number of ambiguous bases compared to all bases.
x: relative N-content of a sequence in percent
y: number of sequences featuring particular N-percentages normalized to the total number of sequences

2.4 Quality distribution

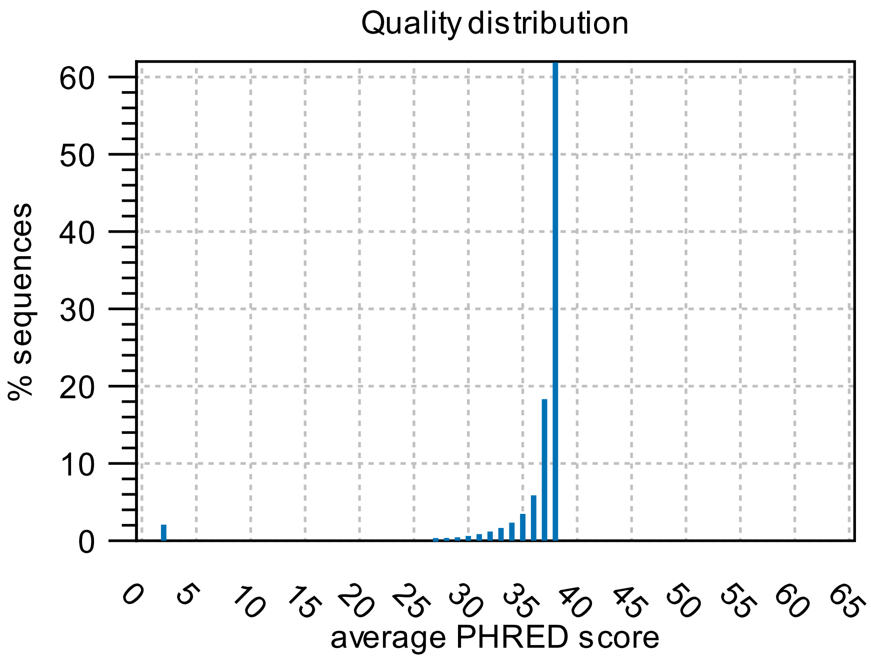

Distribution of average sequence qualitie scores. The quality of a sequence is calculated as the arithmetic mean of its base qualities.
x: PHRED-score
y: number of sequences observed at that qual. score normalized to the total number of sequences

3. Per-base analysis

3.1 Coverage

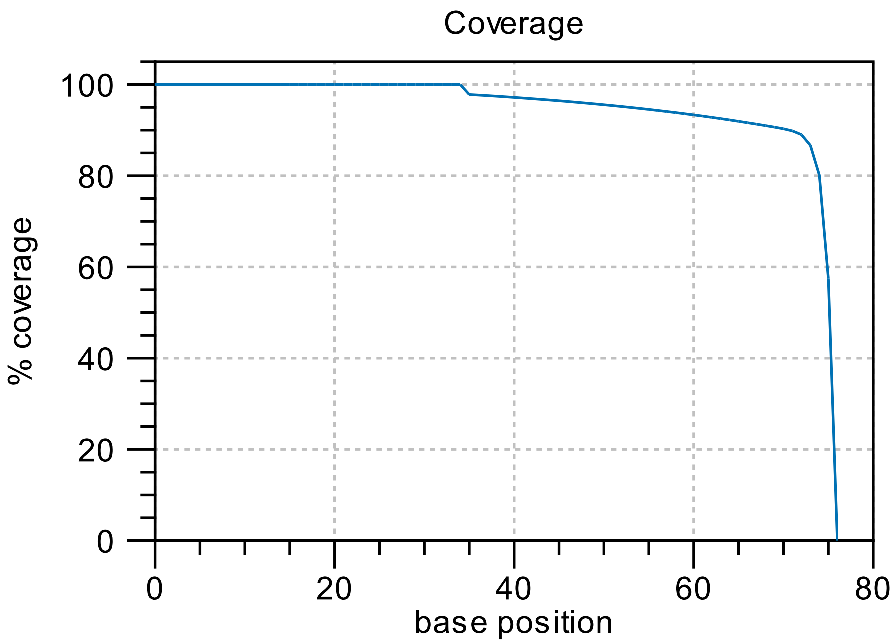

The number of sequences that support (cover) the individual base positions. In cases of untrimmed Illumina or SOLiD reads it will just contain a rectangle.
x: base position
y: number of sequences covering individual base positions normalized to the total number of sequences

3.2 Nucleotide contributions

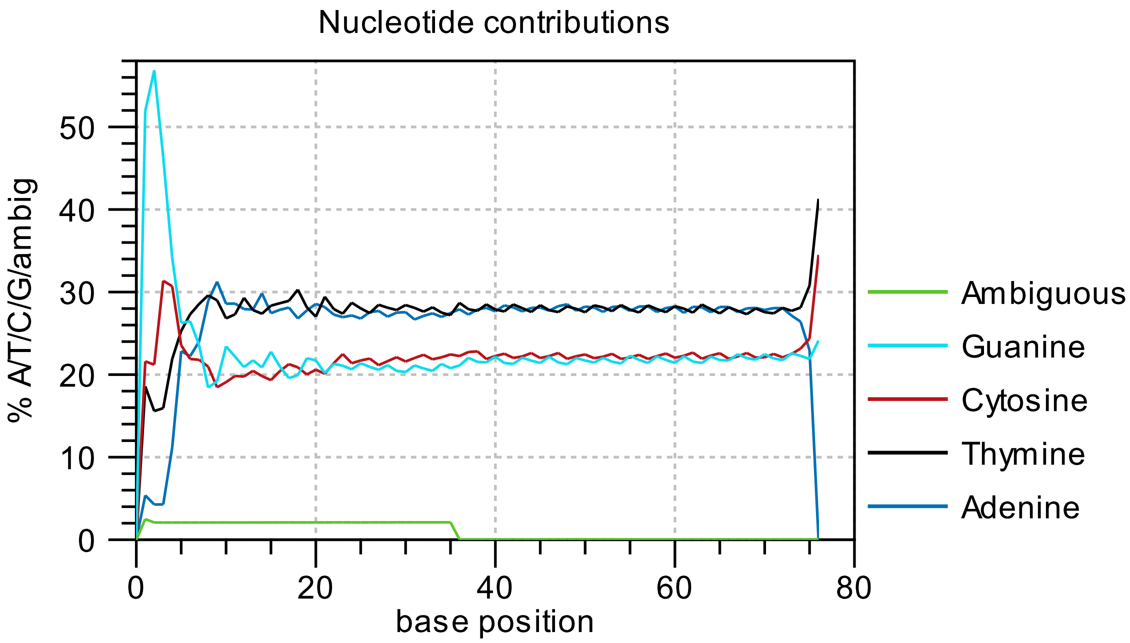

Coverages for the four DNA nucleotides and ambiguous bases.
x: base position
y: number of nucleotides observed per type normalized to the total number of nucleotides observed at that position

3.3 GC-content

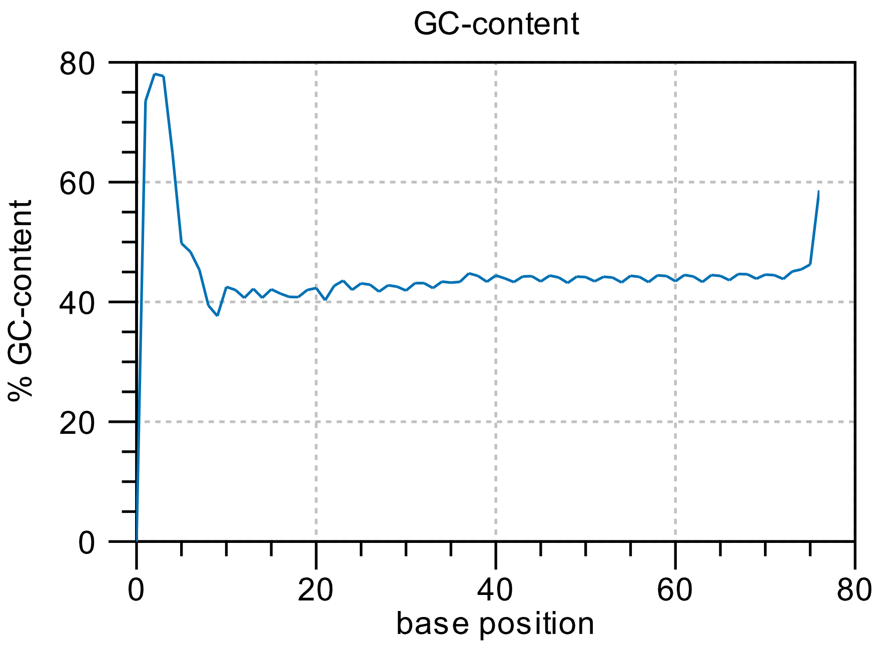

Combined coverage of G- and C-bases.
x: base position
y: number of G- and C-bases observed at current position normalized to the total number of bases observed at that position

3.4 Ambiguous base-content

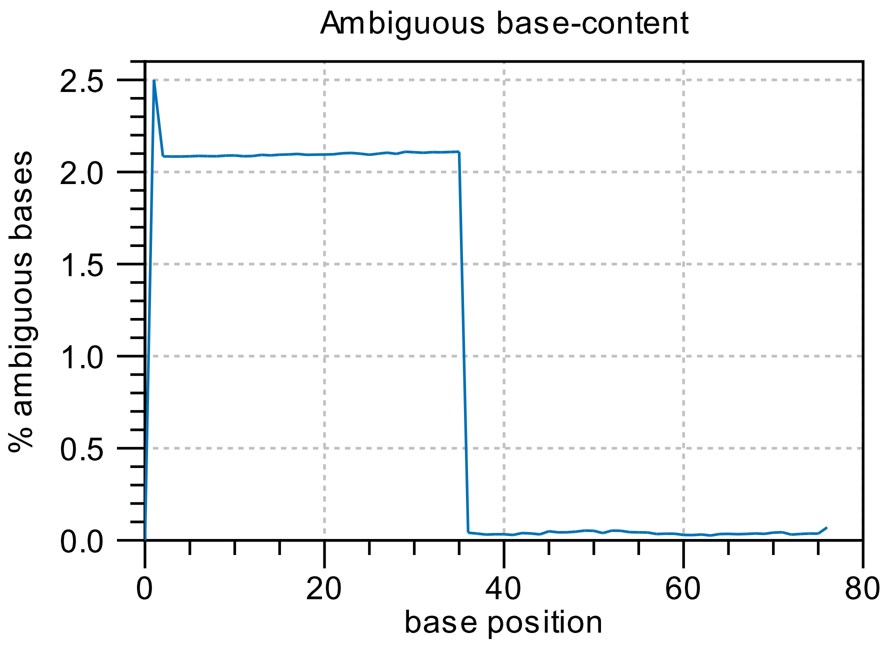

Combined coverage of ambiguous bases.
x: base position
y: number of ambiguous bases observed at current position normalized to the total number of bases observed at that position

3.5 Quality distribution

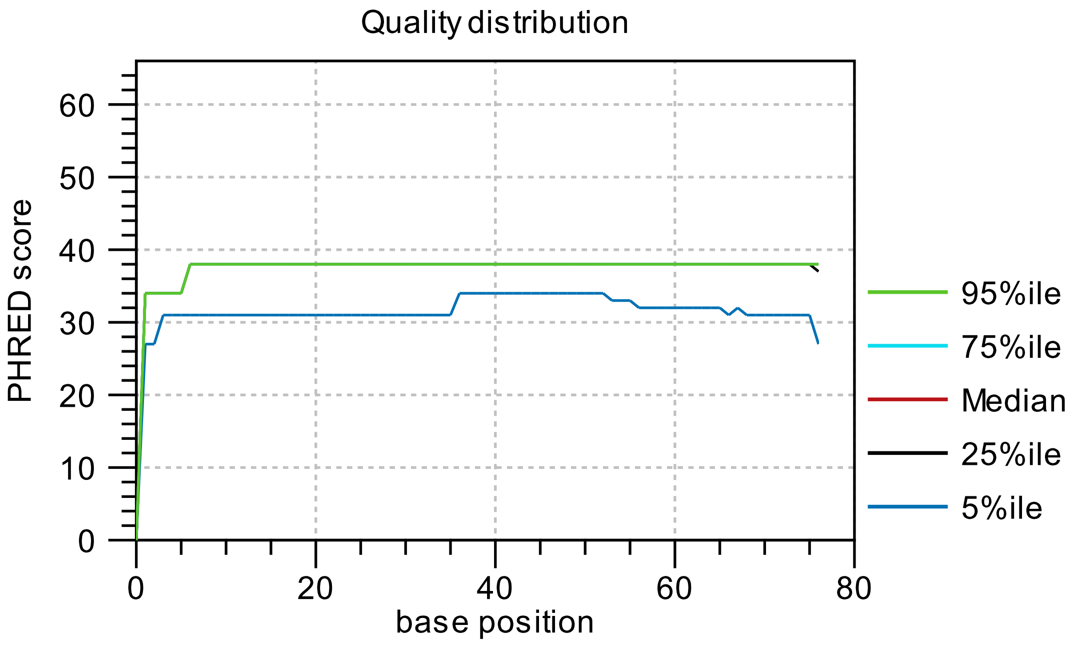

Base-quality distribution along the base positions.
x: base position
y: median & percentiles of quality scores observed at that base position

Sequencing QC Report
Based upon: 44,686,432 sequences in 6 data sets
Generated by: Guerrier
Creation date: Mon Nov 13 15:08:41 CET 2017
Software: CLC Genomics Workbench 9.0.1

Table of contents

1. Summary 3
2. Per-sequence analysis 3
    2.1 Lengths distribution 3
    2.2 GC-content 4
    2.3 Ambiguous base-content 5
    2.4 Quality distribution 6
3. Per-base analysis 6
    3.1 Coverage 7
    3.2 Nucleotide contributions 7
    3.3 GC-content 8
    3.4 Ambiguous base-content 9
    3.5 Quality distribution 9

1. Summary

Creation date:	Mon Nov 13 15:08:41 CET 2017
Generated by:	Guerrier
Software:	CLC Genomics Workbench 9.0.1
Based upon:	6 data sets
H20CTRL1_S10_L001_R1_001 (paired)-1:	6,158,022 sequences in pairs
H20CTRL1_S10_L001_R1_001 (paired)-4:	7,908,178 sequences in pairs
H20CTRL1_S10_L001_R1_001 (paired)-2:	7,186,278 sequences in pairs
H20CTRL1_S10_L001_R1_001 (paired)-3:	7,361,296 sequences in pairs
H20CTRL1_S10_L001_R1_001 (paired):	9,222,594 sequences in pairs
H20C1_S10_L001_R1_001 (paired):	6,850,064 sequences in pairs
Total sequences in data sets	44,686,432 sequences
Total nucleotides in data sets	3,285,660,589 nucleotides

2. Per-sequence analysis

2.1 Lengths distribution

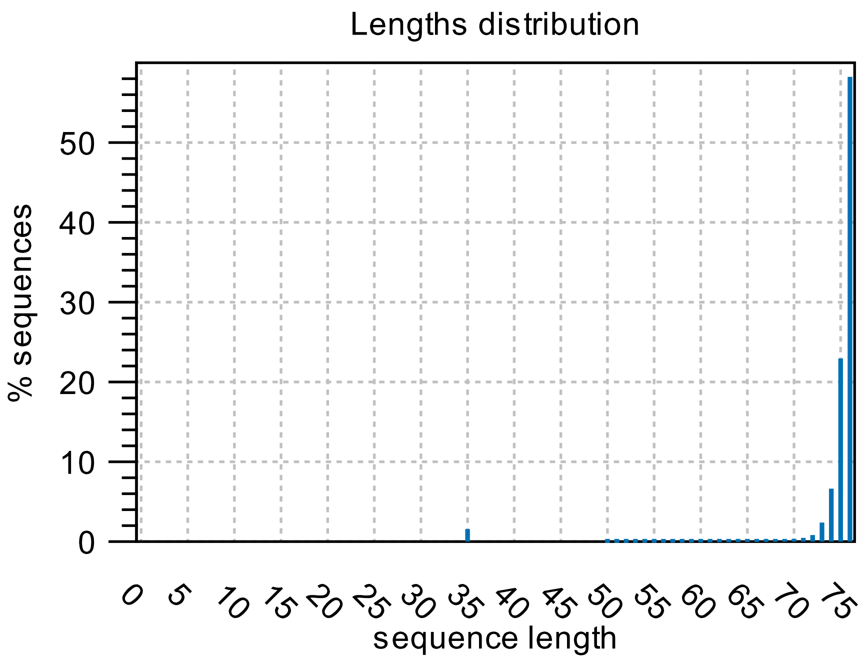

Distribution of sequence lengths. In cases of untrimmed Illumina or SOLiD reads it will just contain a single peak.
x: sequence length in base-pairs

y: number of sequences featuring a particular length normalized to the total number of sequences

2.2 GC-content

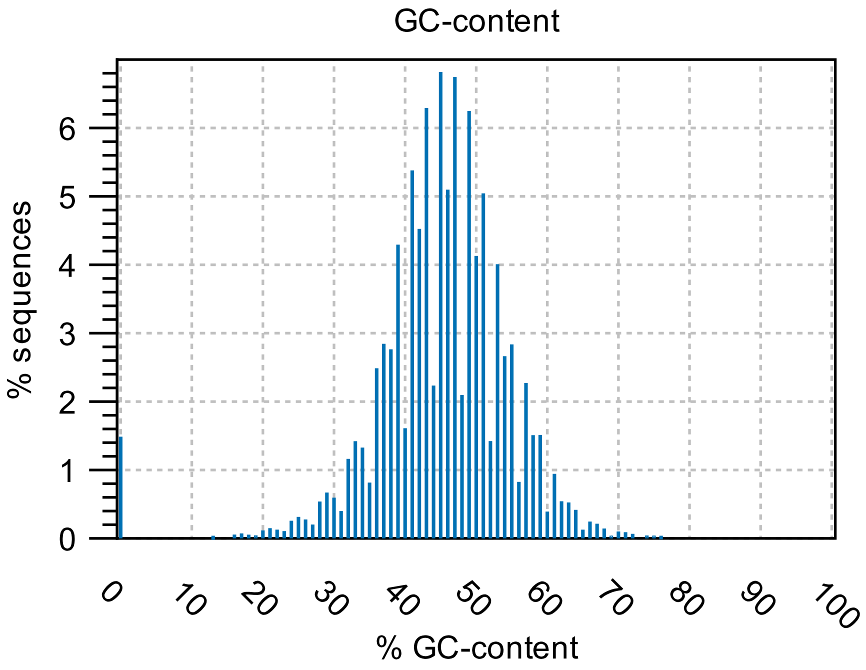

Distribution of GC-contents. The GC-content of a sequence is calculated as the number of GC-bases compared to all bases (including ambiguous bases).
x: relative GC-content of a sequence in percent
y: number of sequences featuring particular GC-percentages normalized to the total number of sequences

2.3 Ambiguous base-content

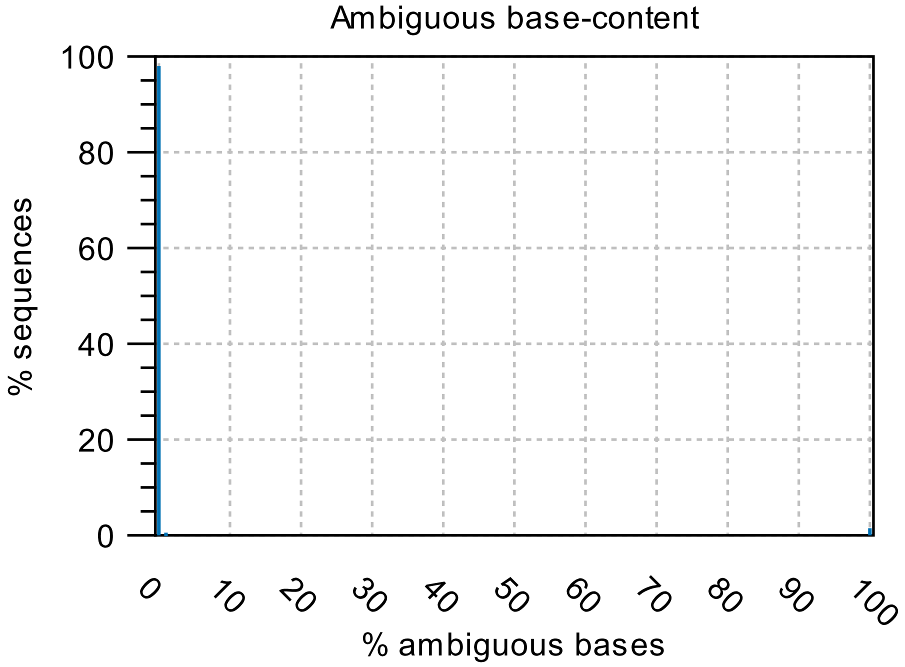

Distribution of N-contents. The N-content of a sequence is calculated as the number of ambiguous bases compared to all bases.
x: relative N-content of a sequence in percent
y: number of sequences featuring particular N-percentages normalized to the total number of sequences

2.4 Quality distribution

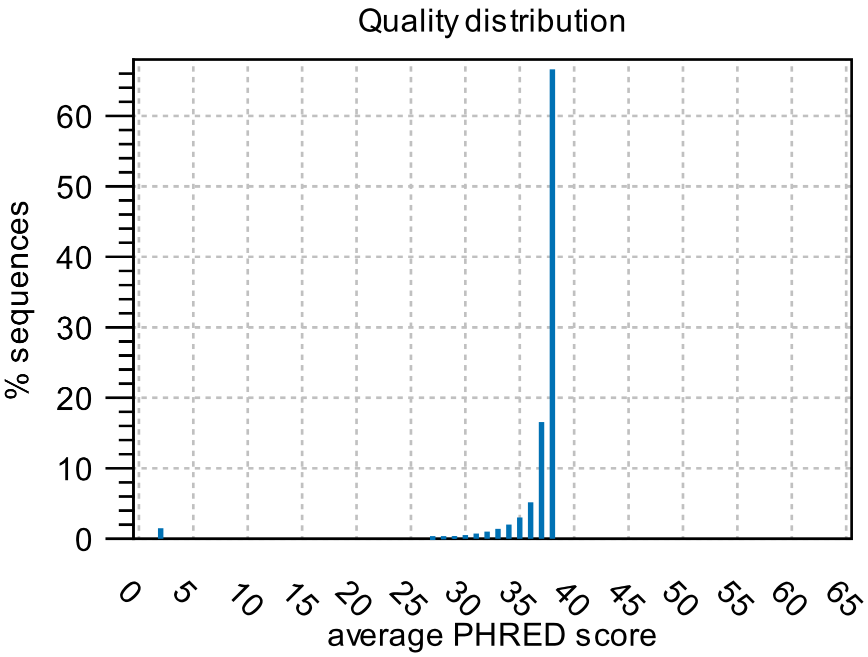

Distribution of average sequence qualitie scores. The quality of a sequence is calculated as the arithmetic mean of its base qualities.
x: PHRED-score
y: number of sequences observed at that qual. score normalized to the total number of sequences

3. Per-base analysis

3.1 Coverage

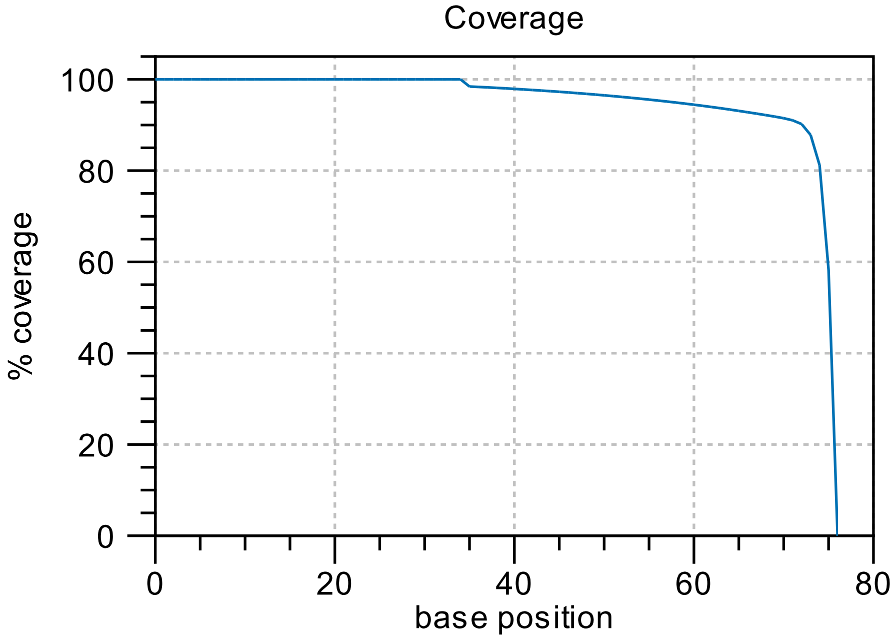

The number of sequences that support (cover) the individual base positions. In cases of untrimmed Illumina or SOLiD reads it will just contain a rectangle.
x: base position
y: number of sequences covering individual base positions normalized to the total number of sequences

3.2 Nucleotide contributions

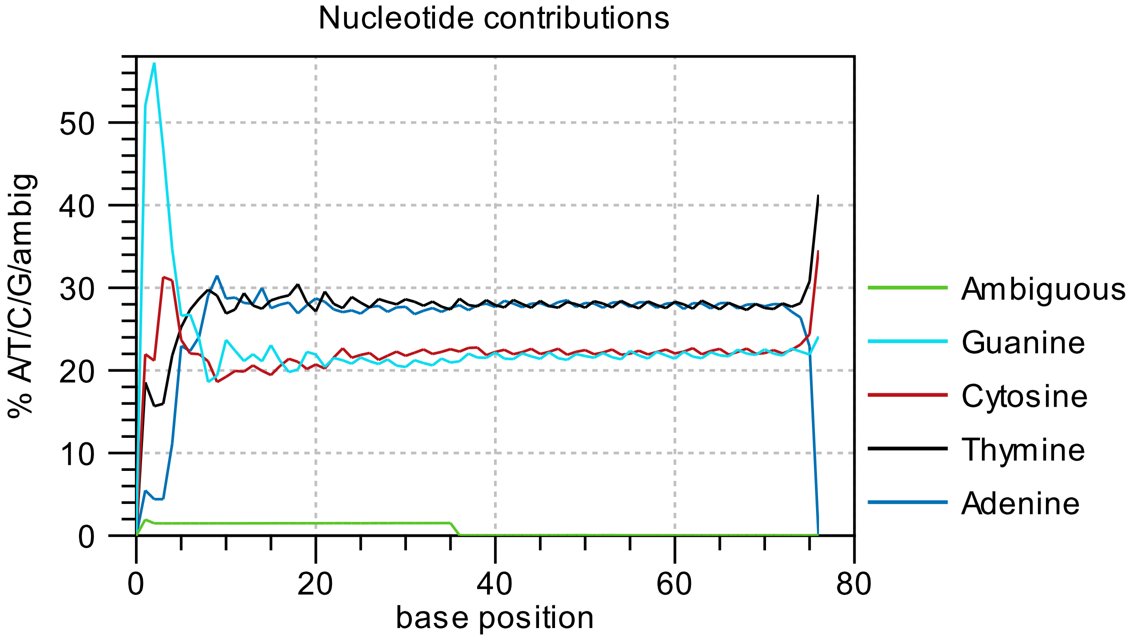

Coverages for the four DNA nucleotides and ambiguous bases.
x: base position
y: number of nucleotides observed per type normalized to the total number of nucleotides observed at that position

3.3 GC-content

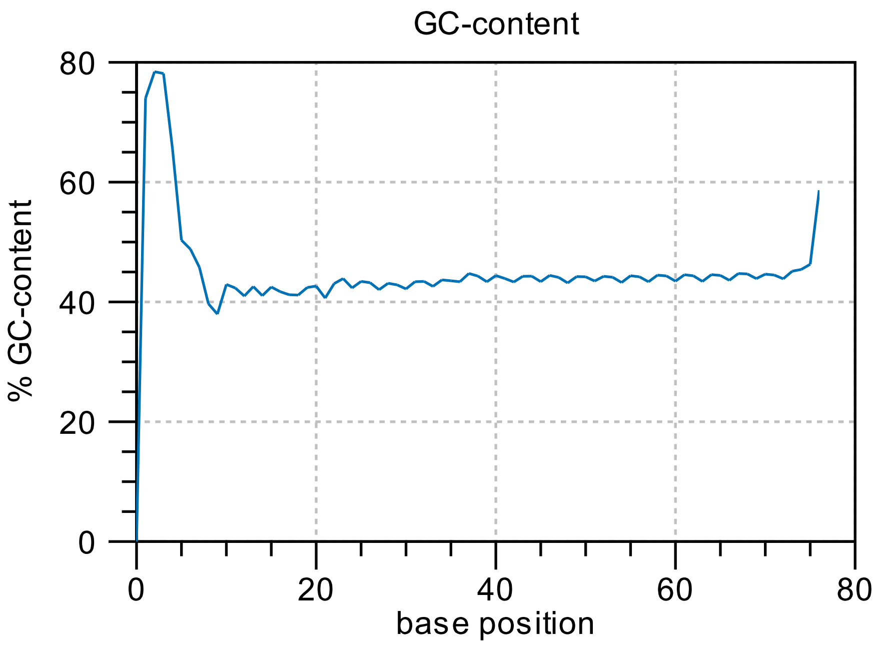

Combined coverage of G- and C-bases.
x: base position
y: number of G- and C-bases observed at current position normalized to the total number of bases observed at that position

3.4 Ambiguous base-content

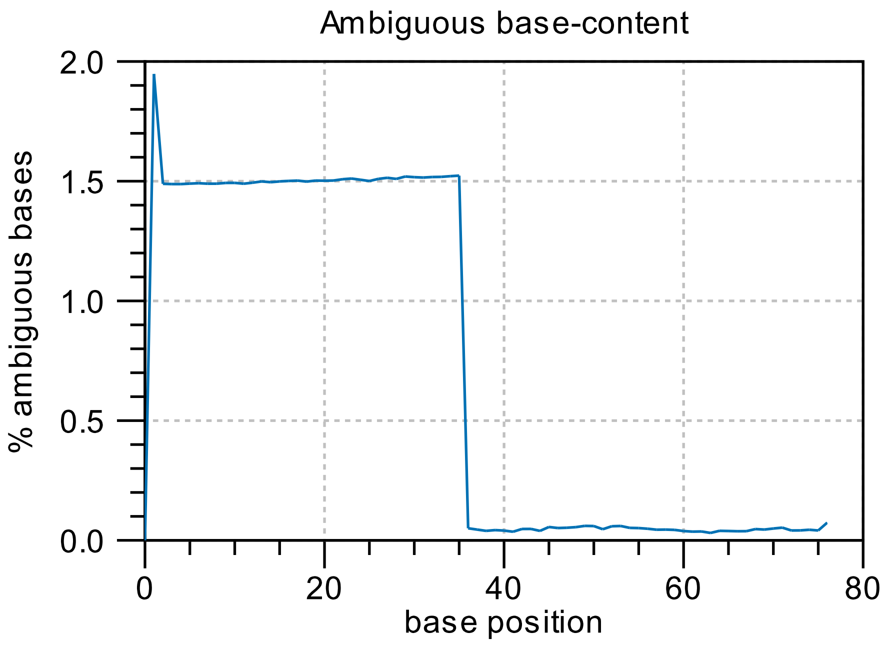

Combined coverage of ambiguous bases.
x: base position
y: number of ambiguous bases observed at current position normalized to the total number of bases observed at that position

3.5 Quality distribution

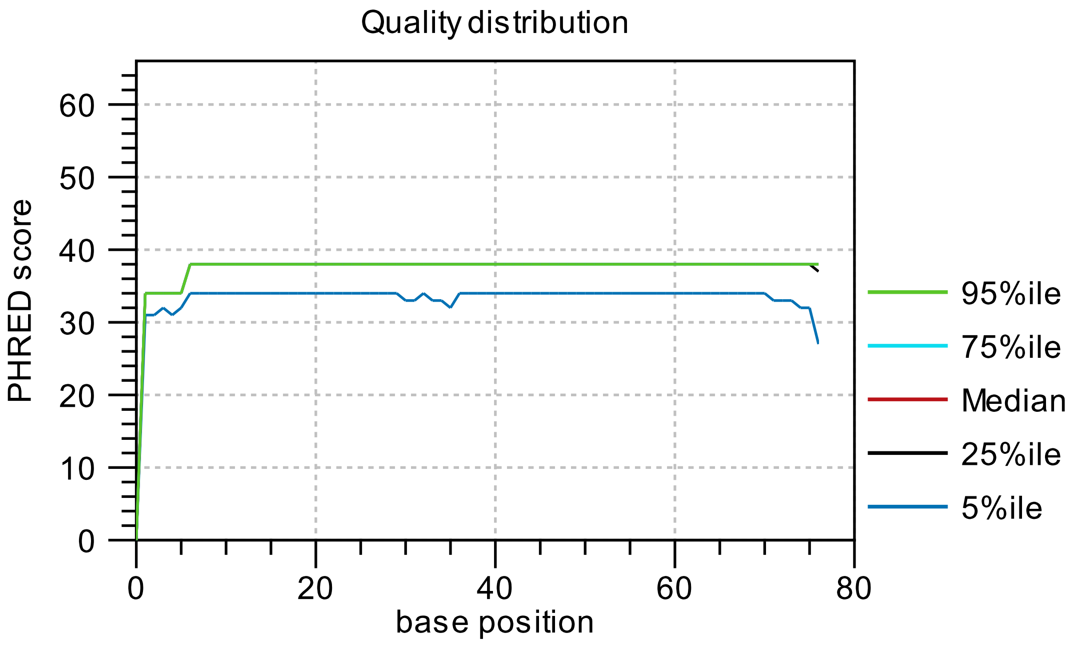

Base-quality distribution along the base positions.
x: base position
y: median & percentiles of quality scores observed at that base position

Sequencing QC Report
Based upon: 24,116,702 sequences in 6 data sets
Generated by: Guerrier
Creation date: Mon Nov 13 15:13:04 CET 2017
Software: CLC Genomics Workbench 9.0.1

Table of contents

1. Summary 3
2. Per-sequence analysis 3
    2.1 Lengths distribution 3
    2.2 GC-content 4
    2.3 Ambiguous base-content 5
    2.4 Quality distribution 6
3. Per-base analysis 6
    3.1 Coverage 7
    3.2 Nucleotide contributions 7
    3.3 GC-content 8
    3.4 Ambiguous base-content 9
    3.5 Quality distribution 9

1. Summary

Creation date:	Mon Nov 13 15:13:04 CET 2017
Generated by:	Guerrier
Software:	CLC Genomics Workbench 9.0.1
Based upon:	6 data sets
H20CTRL2_S11_L001_R1_001 (paired):	5,011,700 sequences in pairs
H20CTRL2_S11_L001_R1_001 (paired)-1:	3,367,740 sequences in pairs
H20C2_S11_L001_R1_001 (paired):	3,692,614 sequences in pairs
H20CTRL2_S11_L001_R1_001 (paired)-3:	3,911,468 sequences in pairs
H20CTRL2_S11_L001_R1_001 (paired)-2:	3,917,418 sequences in pairs
H20CTRL2_S11_L001_R1_001 (paired)-4:	4,215,762 sequences in pairs
Total sequences in data sets	24,116,702 sequences
Total nucleotides in data sets	1,796,464,183 nucleotides

2. Per-sequence analysis

2.1 Lengths distribution

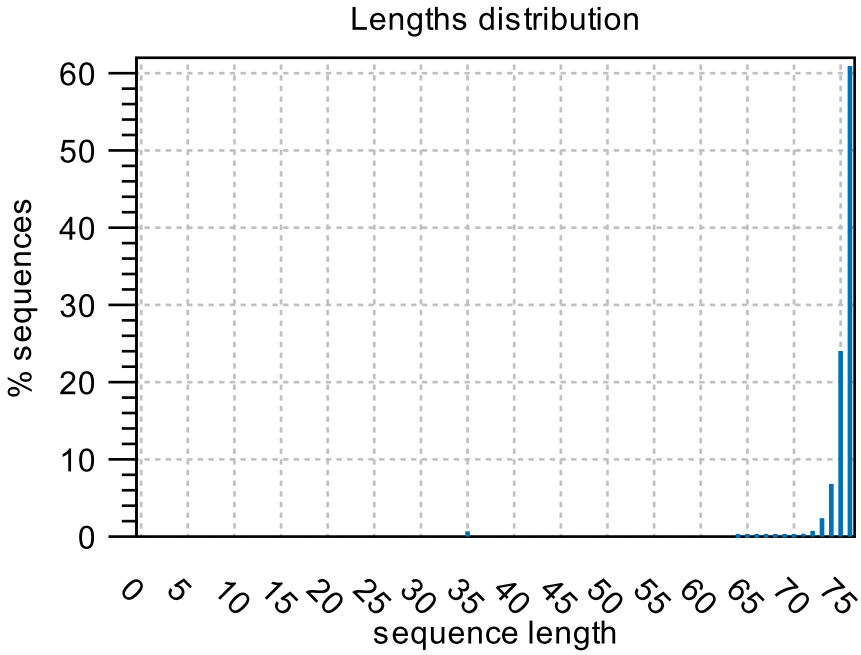

Distribution of sequence lengths. In cases of untrimmed Illumina or SOLiD reads it will just contain a single peak.
x: sequence length in base-pairs

y: number of sequences featuring a particular length normalized to the total number of sequences

2.2 GC-content

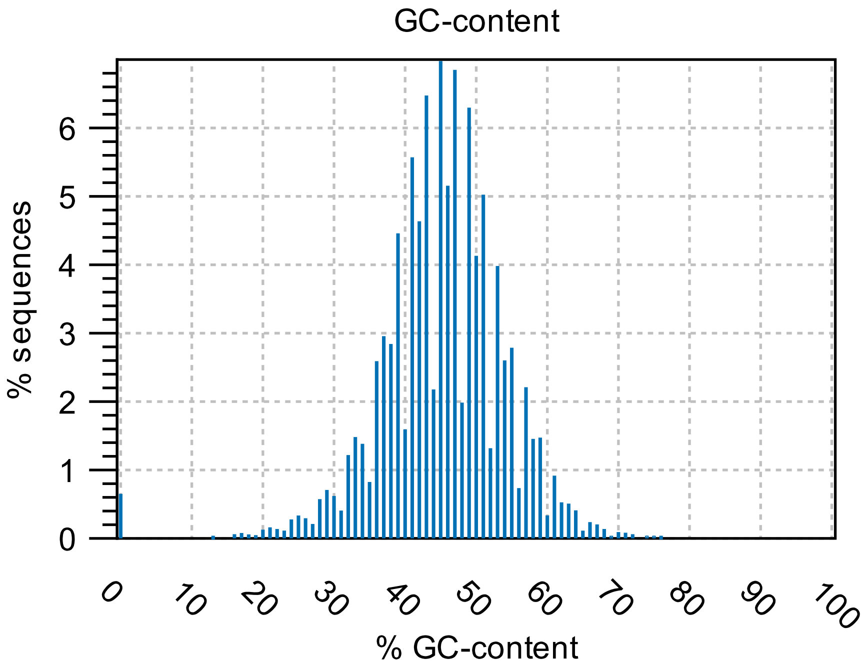

Distribution of GC-contents. The GC-content of a sequence is calculated as the number of GC-bases compared to all bases (including ambiguous bases).
x: relative GC-content of a sequence in percent
y: number of sequences featuring particular GC-percentages normalized to the total number of sequences

2.3 Ambiguous base-content

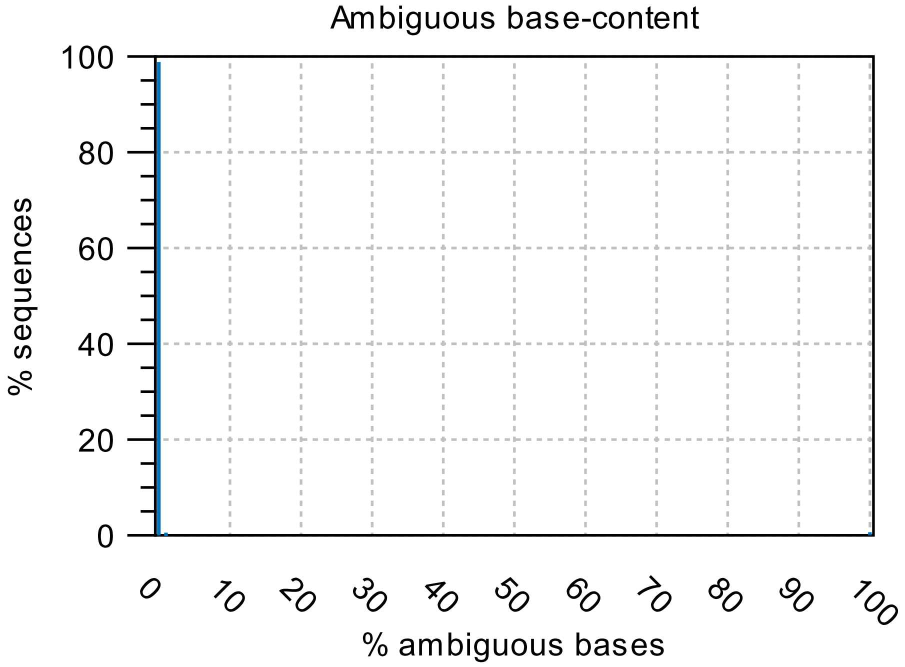

Distribution of N-contents. The N-content of a sequence is calculated as the number of ambiguous bases compared to all bases.
x: relative N-content of a sequence in percent
y: number of sequences featuring particular N-percentages normalized to the total number of sequences

2.4 Quality distribution

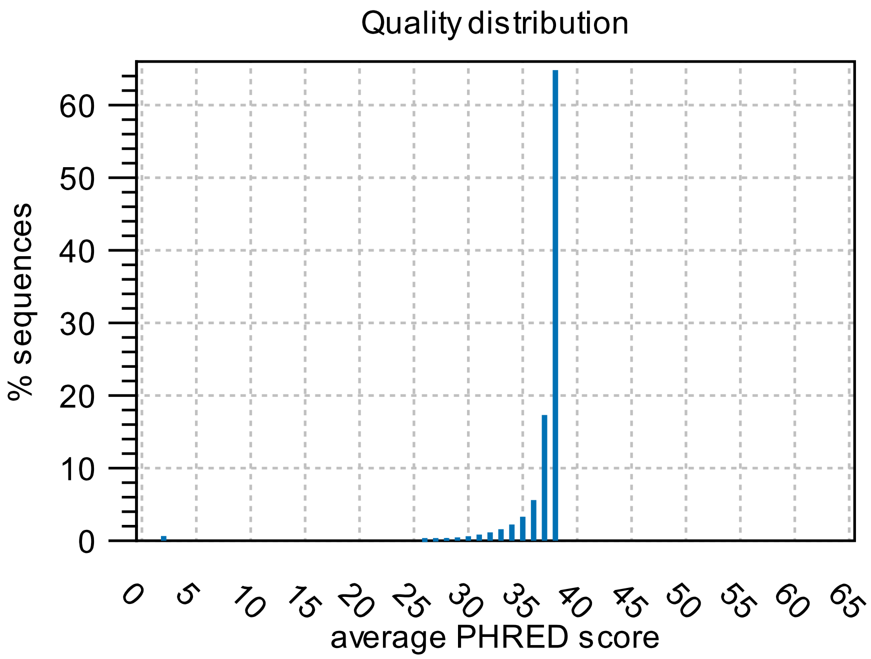

Distribution of average sequence qualitie scores. The quality of a sequence is calculated as the arithmetic mean of its base qualities.
x: PHRED-score
y: number of sequences observed at that qual. score normalized to the total number of sequences

3. Per-base analysis

3.1 Coverage

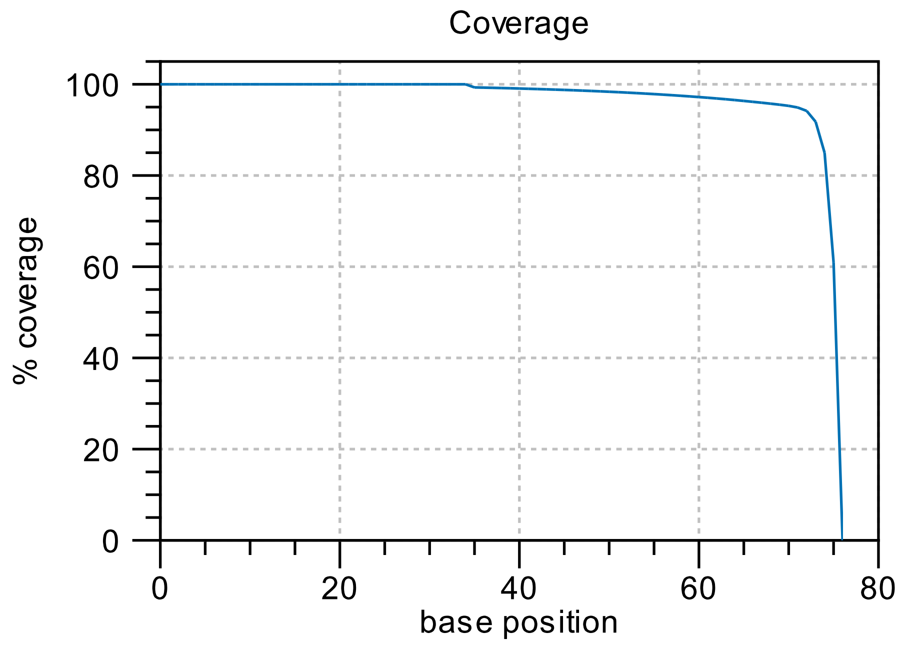

The number of sequences that support (cover) the individual base positions. In cases of untrimmed Illumina or SOLiD reads it will just contain a rectangle.
x: base position
y: number of sequences covering individual base positions normalized to the total number of sequences

3.2 Nucleotide contributions

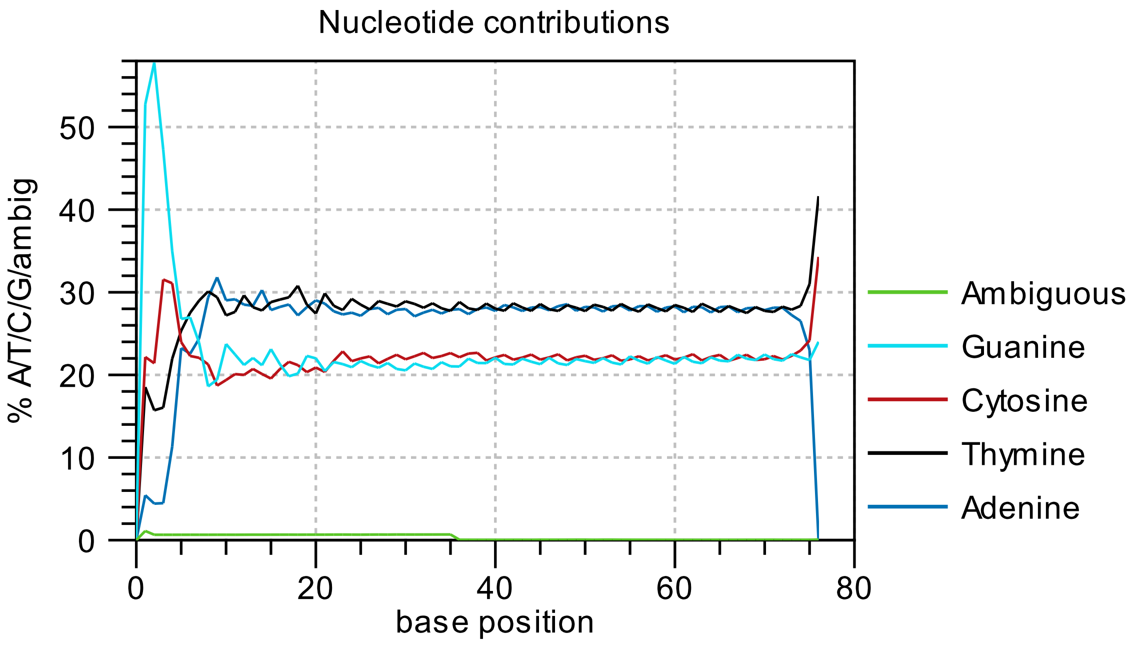

Coverages for the four DNA nucleotides and ambiguous bases.
x: base position
y: number of nucleotides observed per type normalized to the total number of nucleotides observed at that position

3.3 GC-content

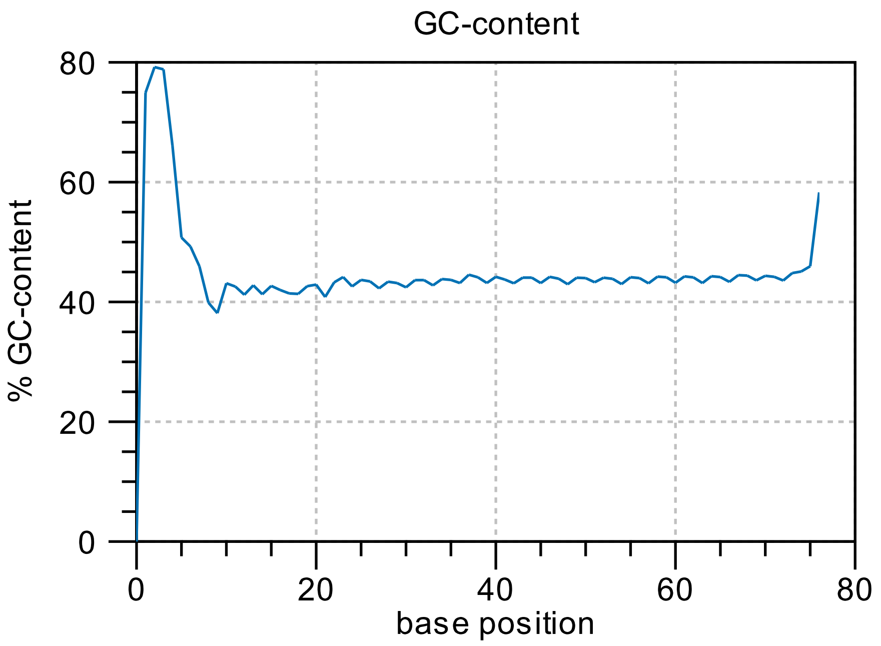

Combined coverage of G- and C-bases.
x: base position
y: number of G- and C-bases observed at current position normalized to the total number of bases observed at that position

3.4 Ambiguous base-content

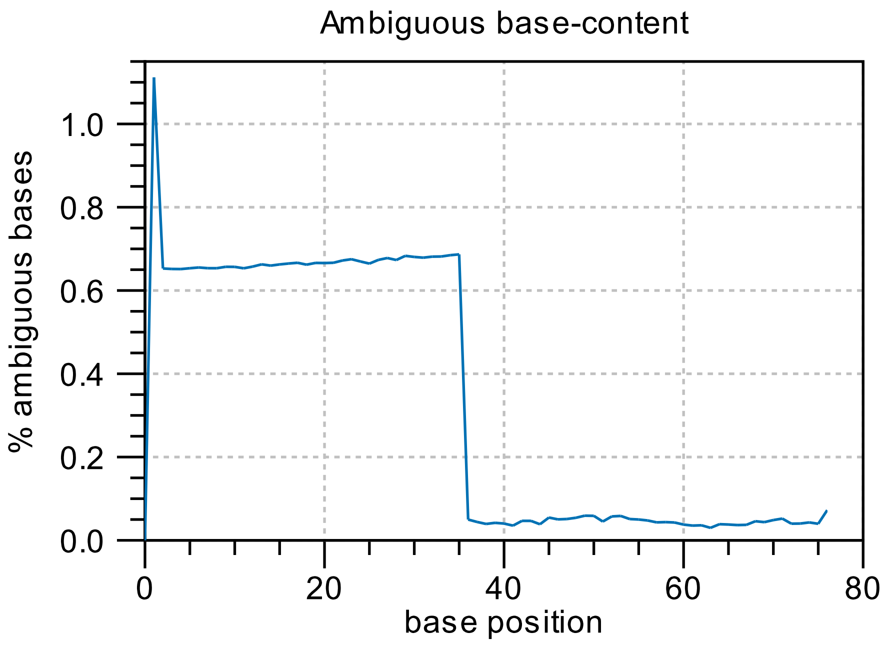

Combined coverage of ambiguous bases.
x: base position
y: number of ambiguous bases observed at current position normalized to the total number of bases observed at that position

3.5 Quality distribution

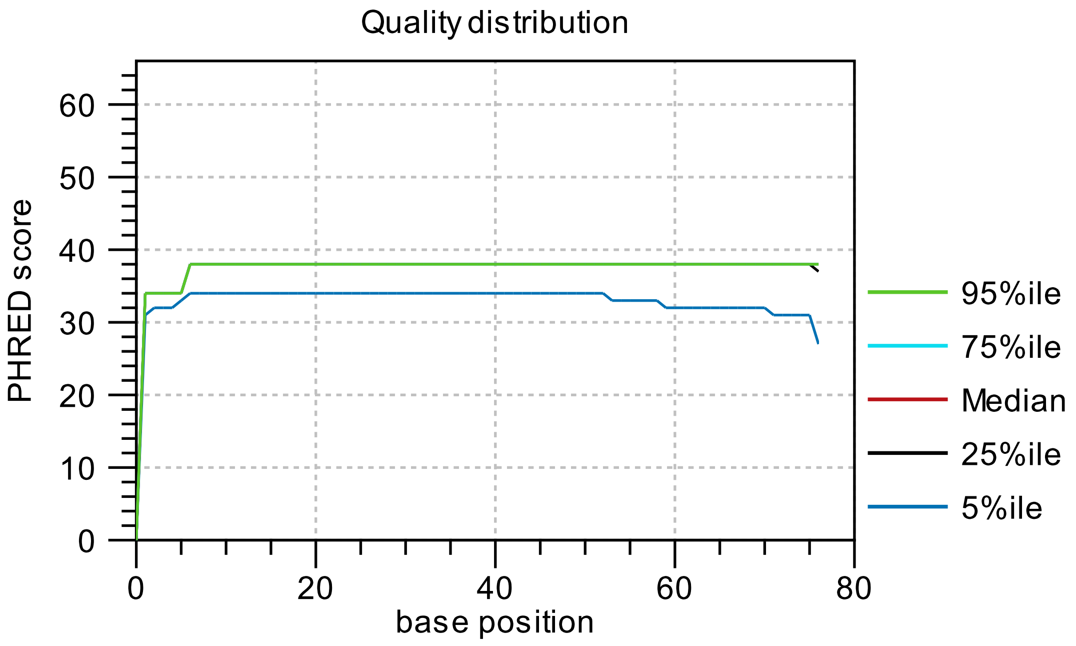

Base-quality distribution along the base positions.
x: base position
y: median & percentiles of quality scores observed at that base position

Sequencing QC Report
Based upon: 19,517,040 sequences in 6 data sets
Generated by: Guerrier
Creation date: Mon Nov 13 15:15:31 CET 2017
Software: CLC Genomics Workbench 9.0.1

Table of contents

1. Summary 3
2. Per-sequence analysis 3
    2.1 Lengths distribution 3
    2.2 GC-content 4
    2.3 Ambiguous base-content 5
    2.4 Quality distribution 6
3. Per-base analysis 6
    3.1 Coverage 7
    3.2 Nucleotide contributions 7
    3.3 GC-content 8
    3.4 Ambiguous base-content 9
    3.5 Quality distribution 9

1. Summary

Creation date:	Mon Nov 13 15:15:31 CET 2017
Generated by:	Guerrier
Software:	CLC Genomics Workbench 9.0.1
Based upon:	6 data sets
H20CTRL3_S12_L001_R1_001 (paired):	2,730,822 sequences in pairs
H20CTRL3_S12_L001_R1_001 (paired)-2:	4,068,282 sequences in pairs
H20CTRL3_S12_L001_R1_001 (paired)-3:	3,175,826 sequences in pairs
H20CTRL3_S12_L001_R1_001 (paired)-1:	3,181,438 sequences in pairs
H20C3_S12_L001_R1_001 (paired):	2,928,388 sequences in pairs
H20CTRL3_S12_L001_R1_001 (paired)-4:	3,432,284 sequences in pairs
Total sequences in data sets	19,517,040 sequences
Total nucleotides in data sets	1,435,215,337 nucleotides

2. Per-sequence analysis

2.1 Lengths distribution

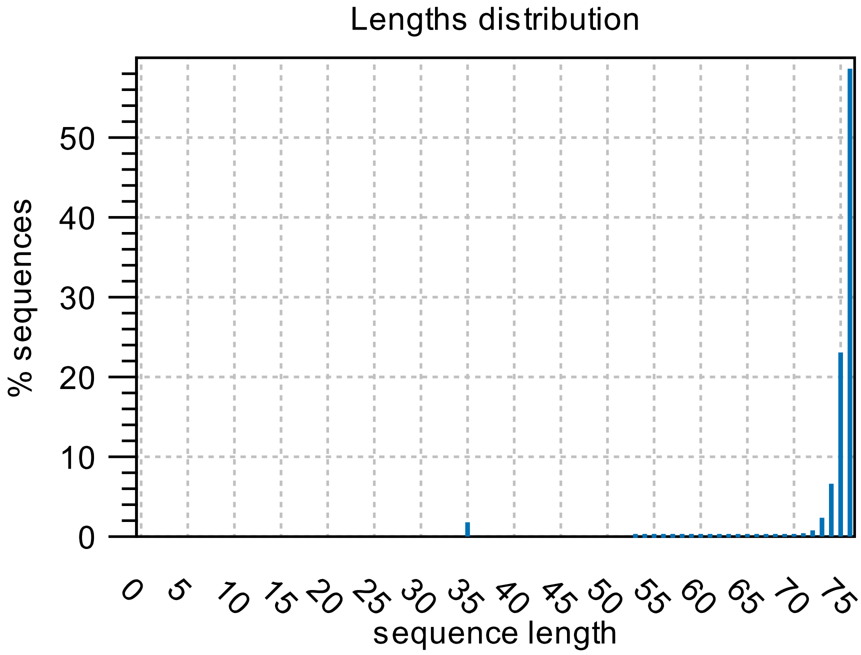

Distribution of sequence lengths. In cases of untrimmed Illumina or SOLiD reads it will just contain a single peak.
x: sequence length in base-pairs

y: number of sequences featuring a particular length normalized to the total number of sequences

2.2 GC-content

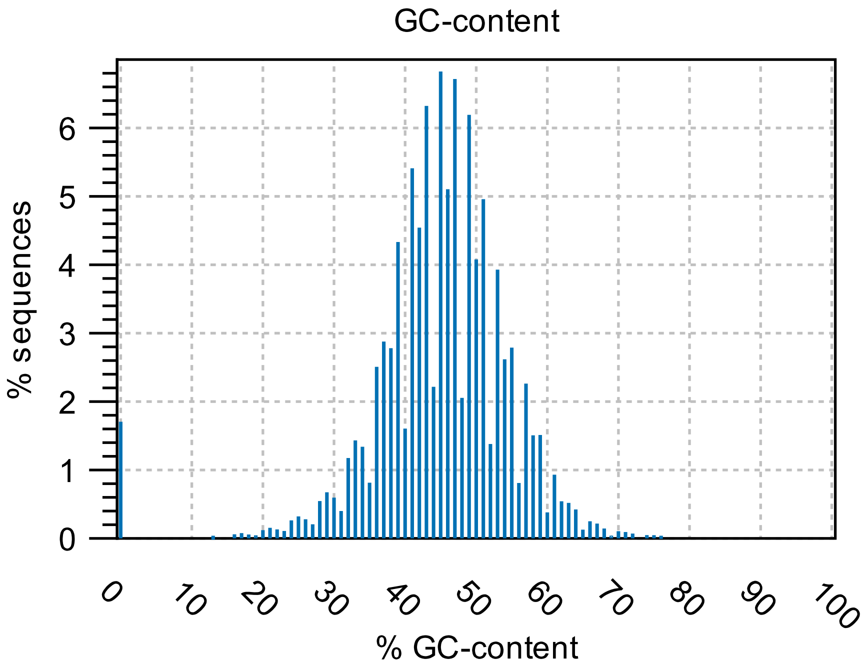

Distribution of GC-contents. The GC-content of a sequence is calculated as the number of GC-bases compared to all bases (including ambiguous bases).
x: relative GC-content of a sequence in percent
y: number of sequences featuring particular GC-percentages normalized to the total number of sequences

2.3 Ambiguous base-content

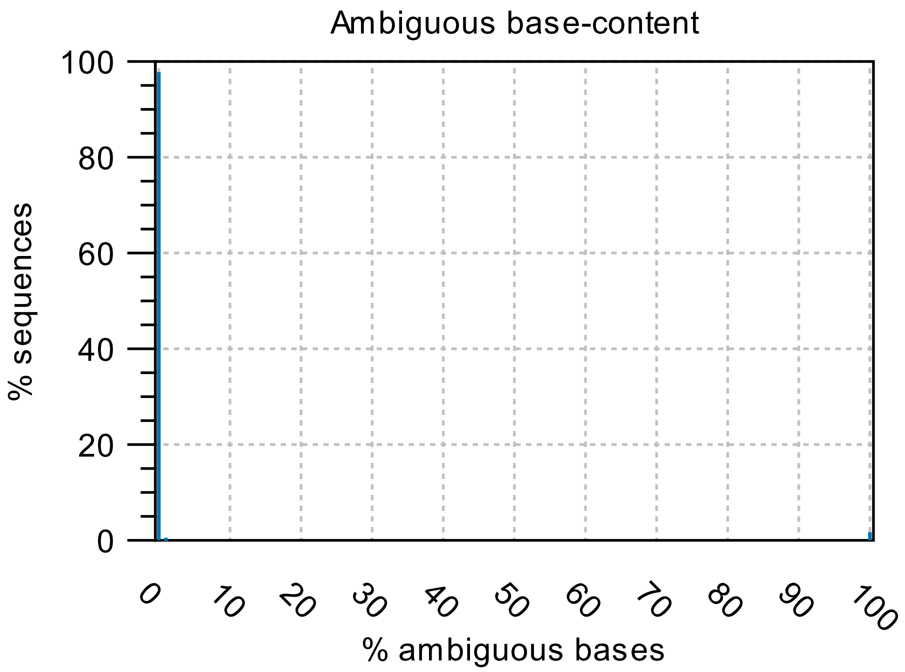

Distribution of N-contents. The N-content of a sequence is calculated as the number of ambiguous bases compared to all bases.
x: relative N-content of a sequence in percent
y: number of sequences featuring particular N-percentages normalized to the total number of sequences

2.4 Quality distribution

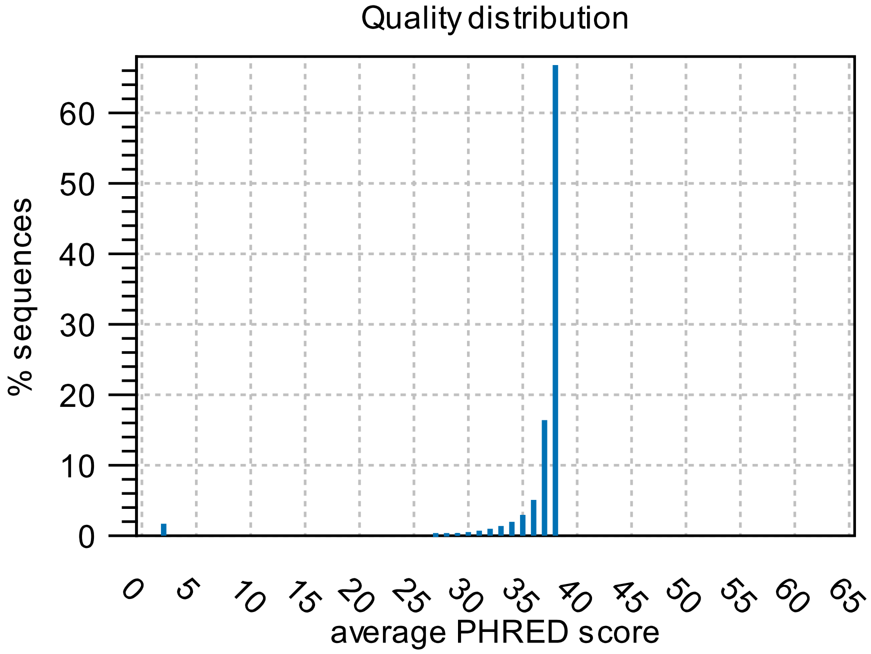

Distribution of average sequence qualitie scores. The quality of a sequence is calculated as the arithmetic mean of its base qualities.
x: PHRED-score
y: number of sequences observed at that qual. score normalized to the total number of sequences

3. Per-base analysis

3.1 Coverage

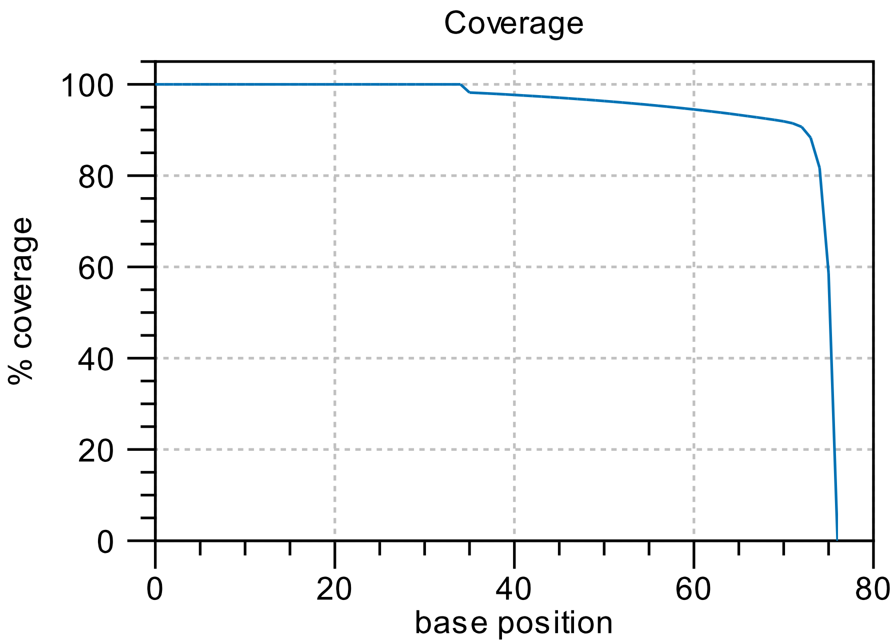

The number of sequences that support (cover) the individual base positions. In cases of untrimmed Illumina or SOLiD reads it will just contain a rectangle.
x: base position
y: number of sequences covering individual base positions normalized to the total number of sequences

3.2 Nucleotide contributions

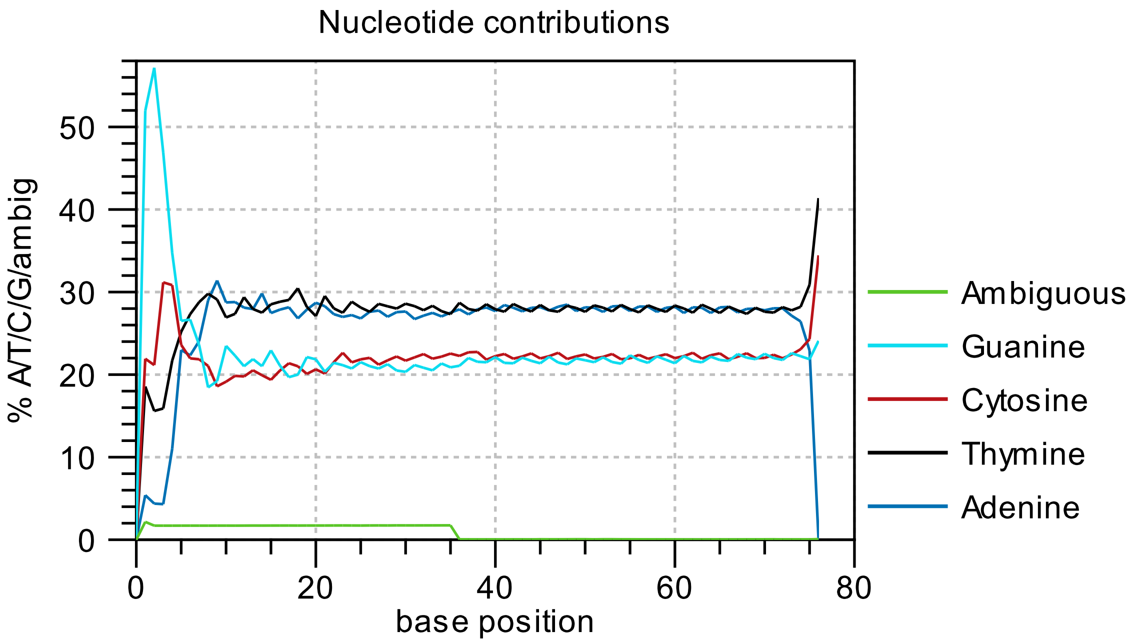

Coverages for the four DNA nucleotides and ambiguous bases.
x: base position
y: number of nucleotides observed per type normalized to the total number of nucleotides observed at that position

3.3 GC-content

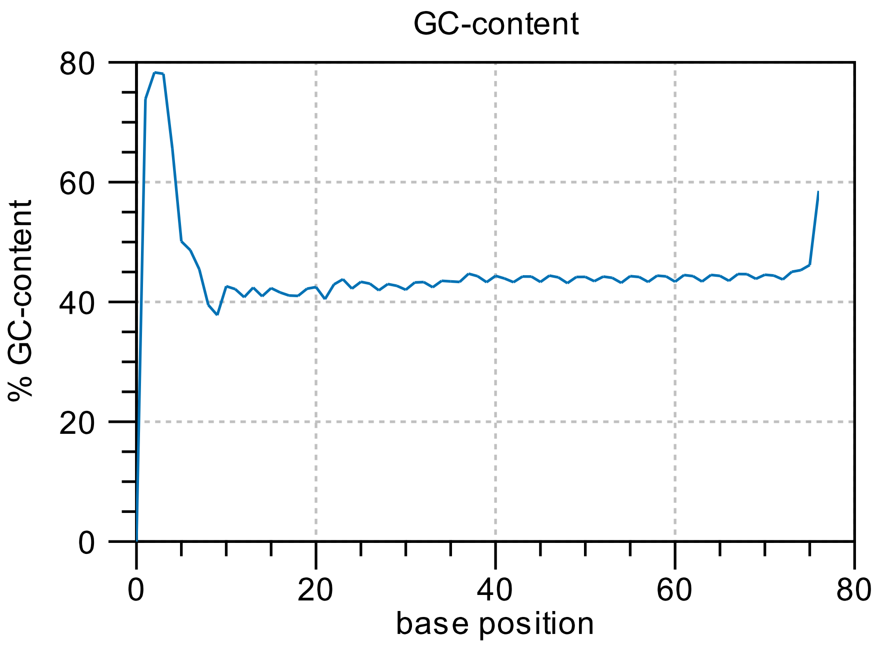

Combined coverage of G- and C-bases.
x: base position
y: number of G- and C-bases observed at current position normalized to the total number of bases observed at that position

3.4 Ambiguous base-content

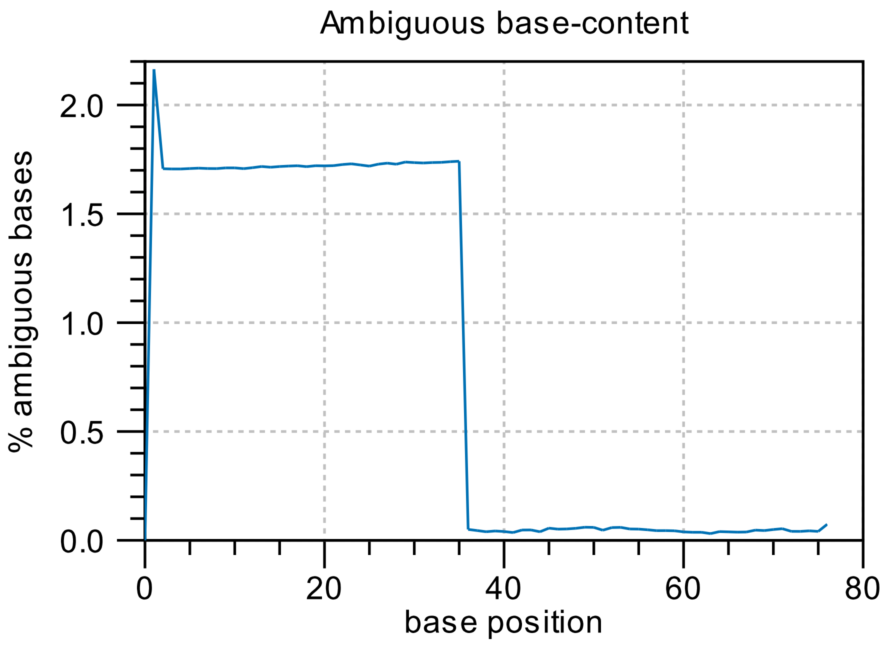

Combined coverage of ambiguous bases.
x: base position
y: number of ambiguous bases observed at current position normalized to the total number of bases observed at that position

3.5 Quality distribution

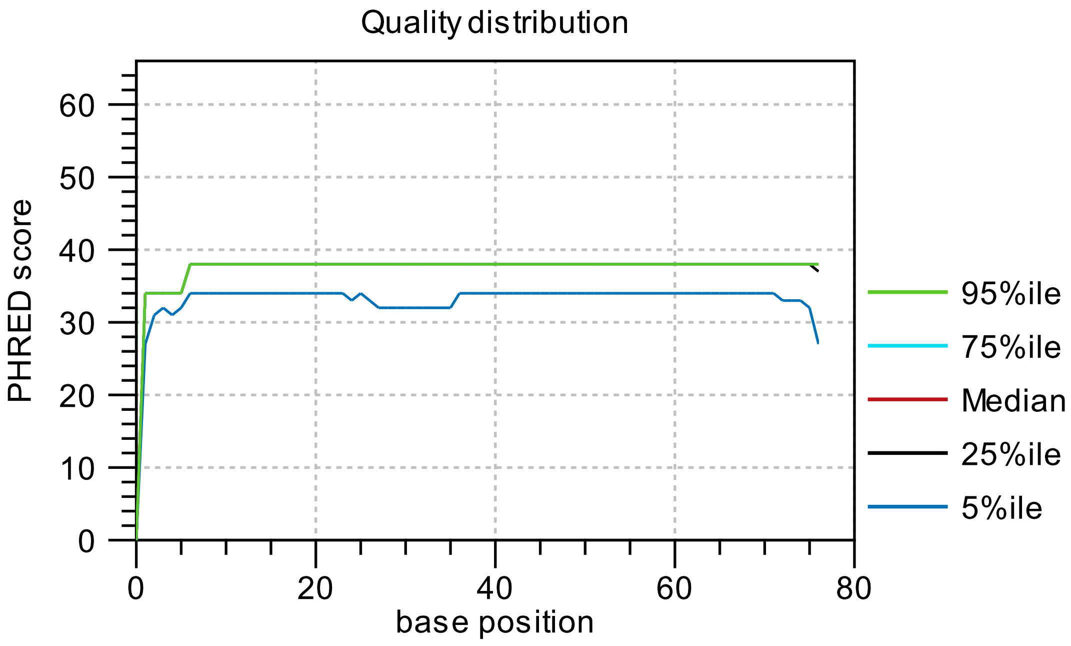

Base-quality distribution along the base positions.
x: base position
y: median & percentiles of quality scores observed at that base position

Sequencing QC Report
Based upon: 31,124,850 sequences in 6 data sets
Generated by: Guerrier
Creation date: Mon Nov 13 15:18:53 CET 2017
Software: CLC Genomics Workbench 9.0.1

Table of contents

1. Summary 3
2. Per-sequence analysis 3
    2.1 Lengths distribution 3
    2.2 GC-content 4
    2.3 Ambiguous base-content 5
    2.4 Quality distribution 6
3. Per-base analysis 6
    3.1 Coverage 7
    3.2 Nucleotide contributions 7
    3.3 GC-content 8
    3.4 Ambiguous base-content 9
    3.5 Quality distribution 9

1. Summary

Creation date:	Mon Nov 13 15:18:53 CET 2017
Generated by:	Guerrier
Software:	CLC Genomics Workbench 9.0.1
Based upon:	6 data sets
H20JA1_S7_L001_R1_001 (paired):	1,689,996 sequences in pairs
H20JA1_S7_L001_R1_001 (paired)-3:	6,572,320 sequences in pairs
H20JA1_S7_L001_R1_001 (paired)-1:	5,080,386 sequences in pairs
H20JA1_S7_L001_R1_001 (paired)-5:	5,678,292 sequences in pairs
H20JA1_S7_L001_R1_001 (paired)-2:	5,988,480 sequences in pairs
H20JA1_S7_L001_R1_001 (paired)-4:	6,115,376 sequences in pairs
Total sequences in data sets	31,124,850 sequences
Total nucleotides in data sets	2,279,616,073 nucleotides

2. Per-sequence analysis

2.1 Lengths distribution

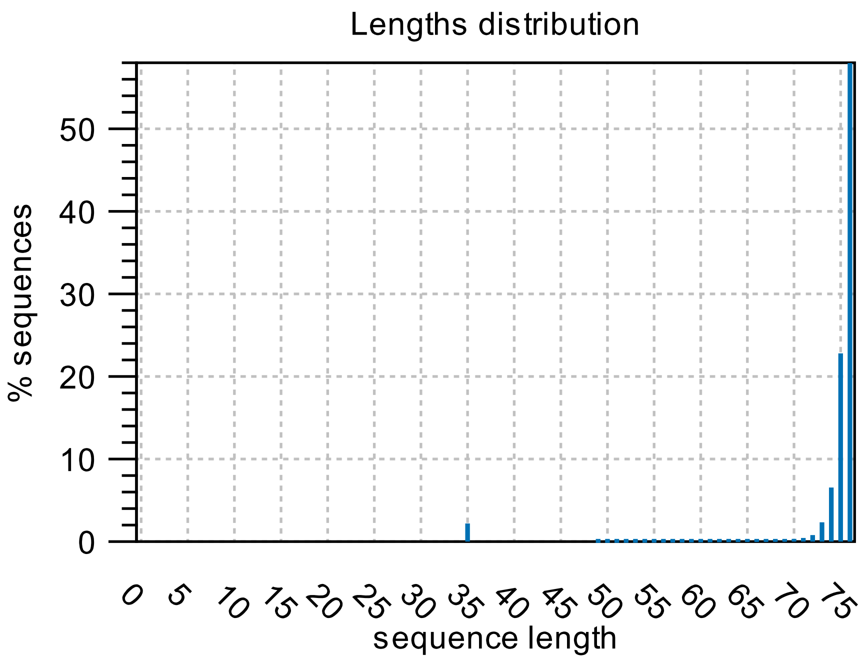

Distribution of sequence lengths. In cases of untrimmed Illumina or SOLiD reads it will just contain a single peak.
x: sequence length in base-pairs

y: number of sequences featuring a particular length normalized to the total number of sequences

2.2 GC-content

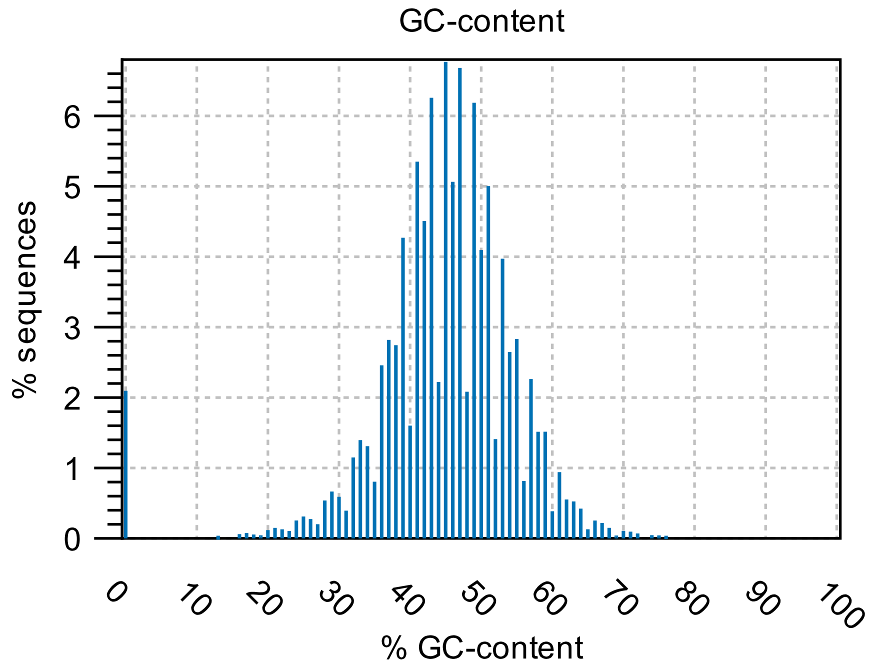

Distribution of GC-contents. The GC-content of a sequence is calculated as the number of GC-bases compared to all bases (including ambiguous bases).
x: relative GC-content of a sequence in percent
y: number of sequences featuring particular GC-percentages normalized to the total number of sequences

2.3 Ambiguous base-content

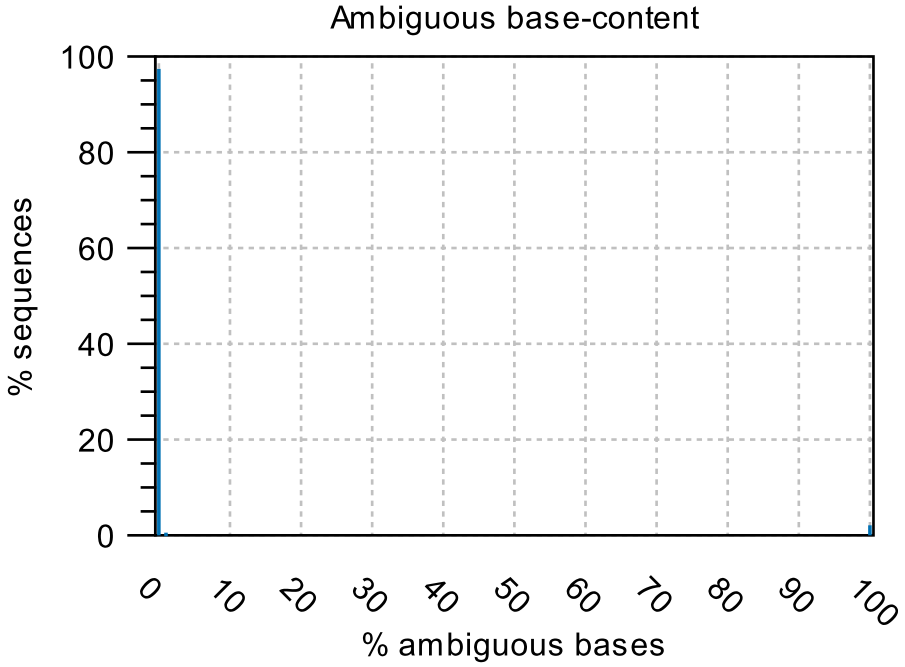

Distribution of N-contents. The N-content of a sequence is calculated as the number of ambiguous bases compared to all bases.
x: relative N-content of a sequence in percent
y: number of sequences featuring particular N-percentages normalized to the total number of sequences

2.4 Quality distribution

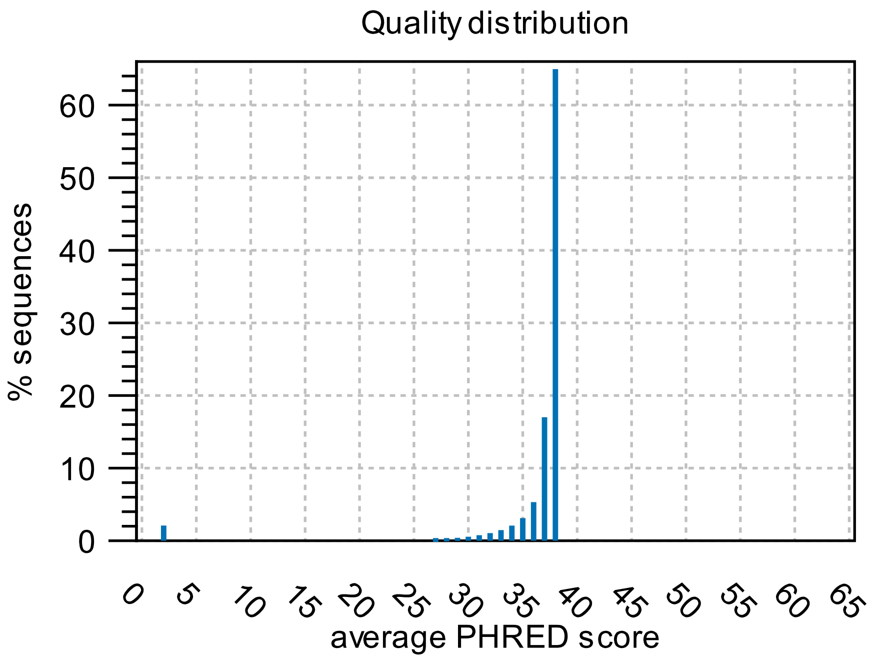

Distribution of average sequence qualitie scores. The quality of a sequence is calculated as the arithmetic mean of its base qualities.
x: PHRED-score
y: number of sequences observed at that qual. score normalized to the total number of sequences

3. Per-base analysis

3.1 Coverage

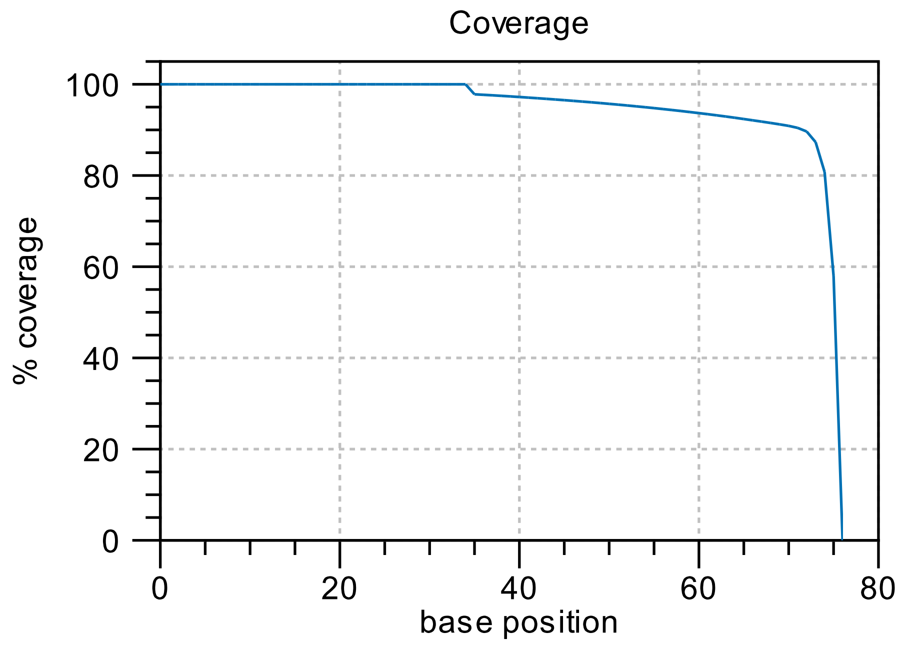

The number of sequences that support (cover) the individual base positions. In cases of untrimmed Illumina or SOLiD reads it will just contain a rectangle.
x: base position
y: number of sequences covering individual base positions normalized to the total number of sequences

3.2 Nucleotide contributions

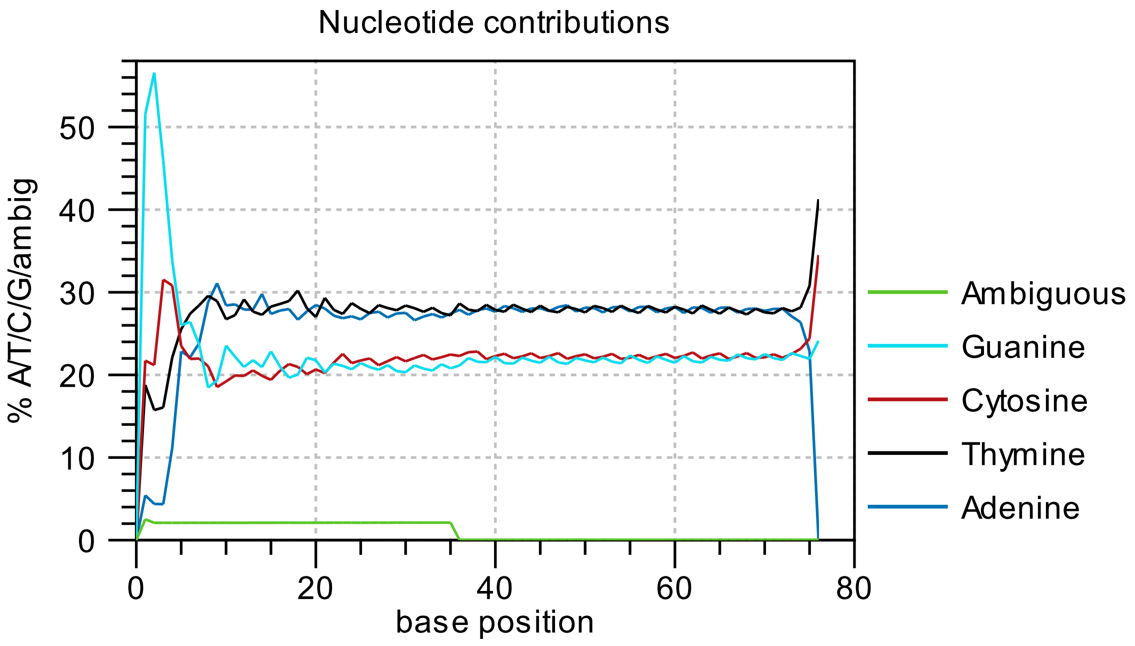

Coverages for the four DNA nucleotides and ambiguous bases.
x: base position
y: number of nucleotides observed per type normalized to the total number of nucleotides observed at that position

3.3 GC-content

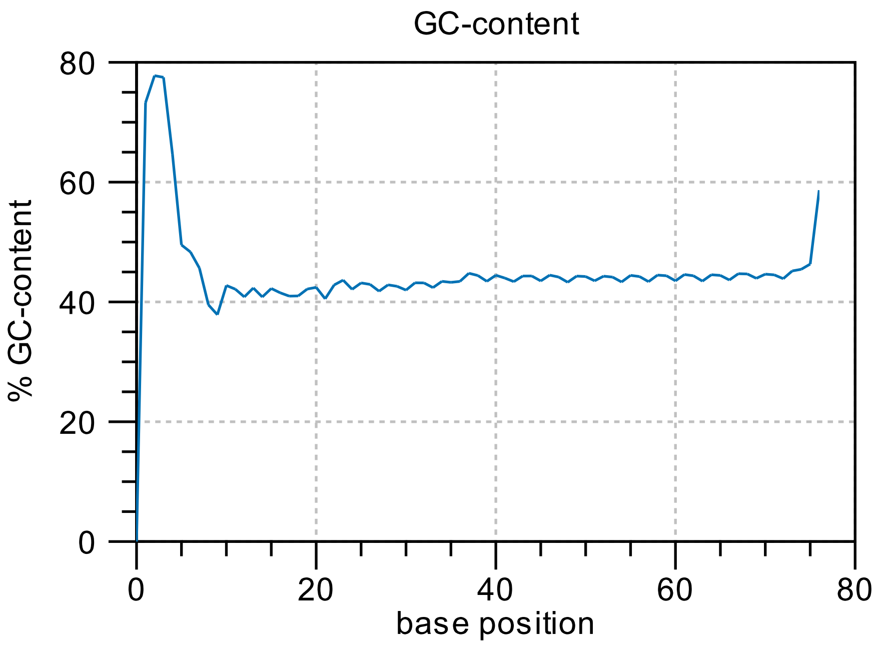

Combined coverage of G- and C-bases.
x: base position
y: number of G- and C-bases observed at current position normalized to the total number of bases observed at that position

3.4 Ambiguous base-content

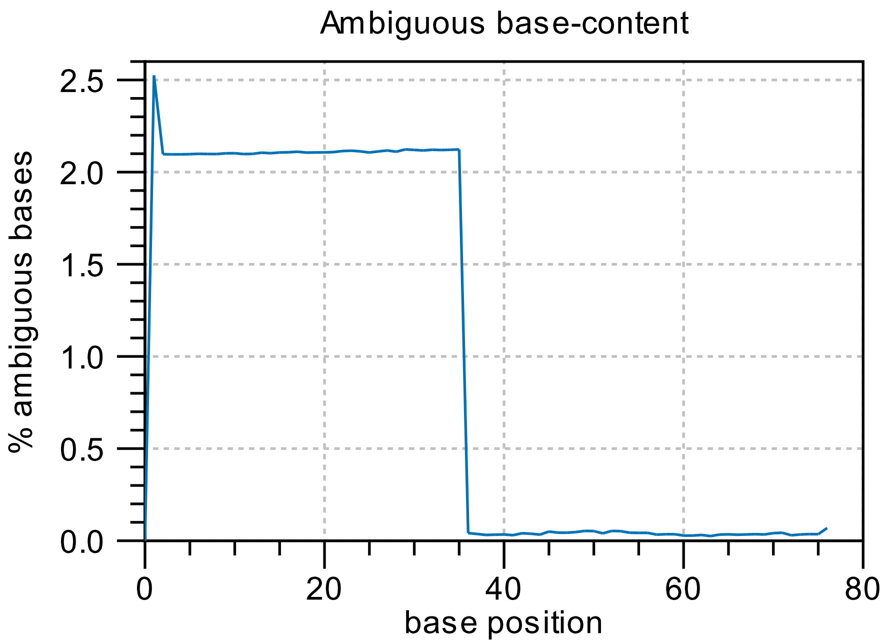

Combined coverage of ambiguous bases.
x: base position
y: number of ambiguous bases observed at current position normalized to the total number of bases observed at that position

3.5 Quality distribution

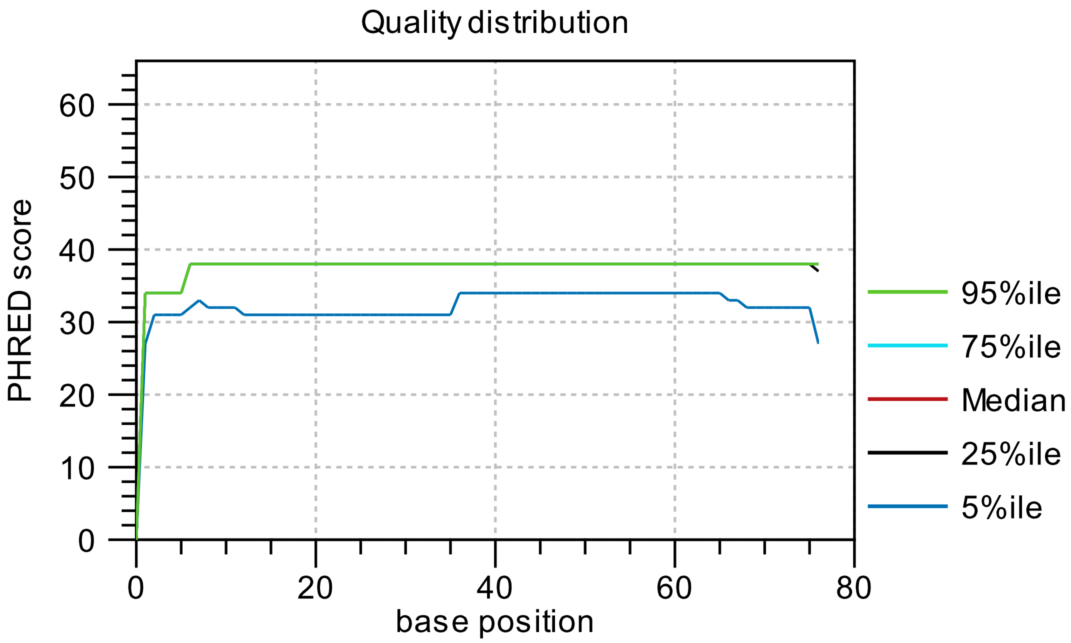

Base-quality distribution along the base positions.
x: base position
y: median & percentiles of quality scores observed at that base position

Sequencing QC Report
Based upon: 19,884,562 sequences in 6 data sets
Generated by: Guerrier
Creation date: Mon Nov 13 15:21:21 CET 2017
Software: CLC Genomics Workbench 9.0.1

Table of contents

1. Summary 3
2. Per-sequence analysis 3
    2.1 Lengths distribution 3
    2.2 GC-content 4
    2.3 Ambiguous base-content 5
    2.4 Quality distribution 6
3. Per-base analysis 6
    3.1 Coverage 7
    3.2 Nucleotide contributions 7
    3.3 GC-content 8
    3.4 Ambiguous base-content 9
    3.5 Quality distribution 9

1. Summary

Creation date:	Mon Nov 13 15:21:21 CET 2017
Generated by:	Guerrier
Software:	CLC Genomics Workbench 9.0.1
Based upon:	6 data sets
H20JA2_S8_L001_R1_001 (paired):	2,101,306 sequences in pairs
H20JA2_S8_L001_R1_001 (paired)-1:	3,158,630 sequences in pairs
H20JA2_S8_L001_R1_001 (paired)-4:	3,640,424 sequences in pairs
H20JA2_S8_L001_R1_001 (paired)-5:	3,393,434 sequences in pairs
H20JA2_S8_L001_R1_001 (paired)-2:	3,672,410 sequences in pairs
H20JA2_S8_L001_R1_001 (paired)-3:	3,918,358 sequences in pairs
Total sequences in data sets	19,884,562 sequences
Total nucleotides in data sets	1,460,151,545 nucleotides

2. Per-sequence analysis

2.1 Lengths distribution

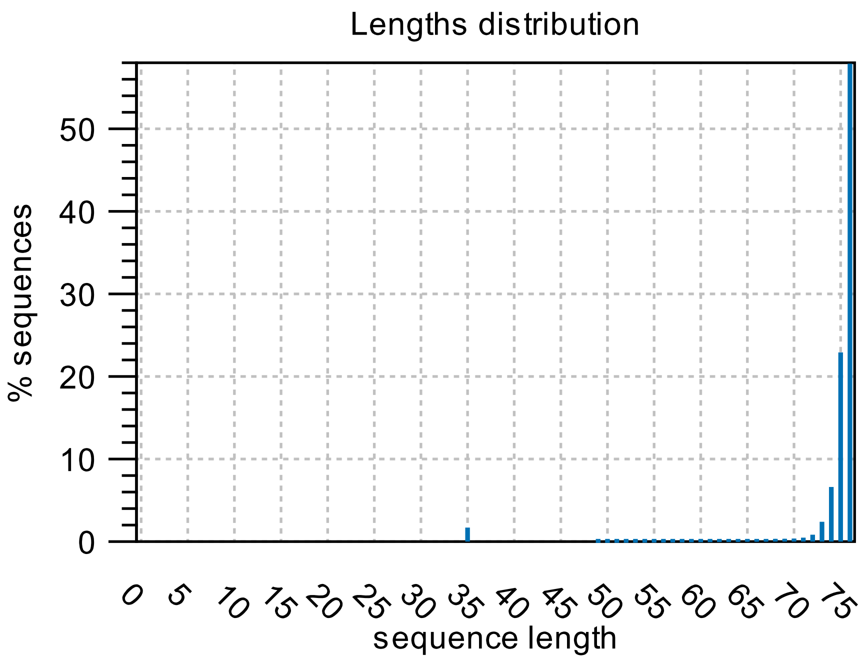

Distribution of sequence lengths. In cases of untrimmed Illumina or SOLiD reads it will just contain a single peak.
x: sequence length in base-pairs

y: number of sequences featuring a particular length normalized to the total number of sequences

2.2 GC-content

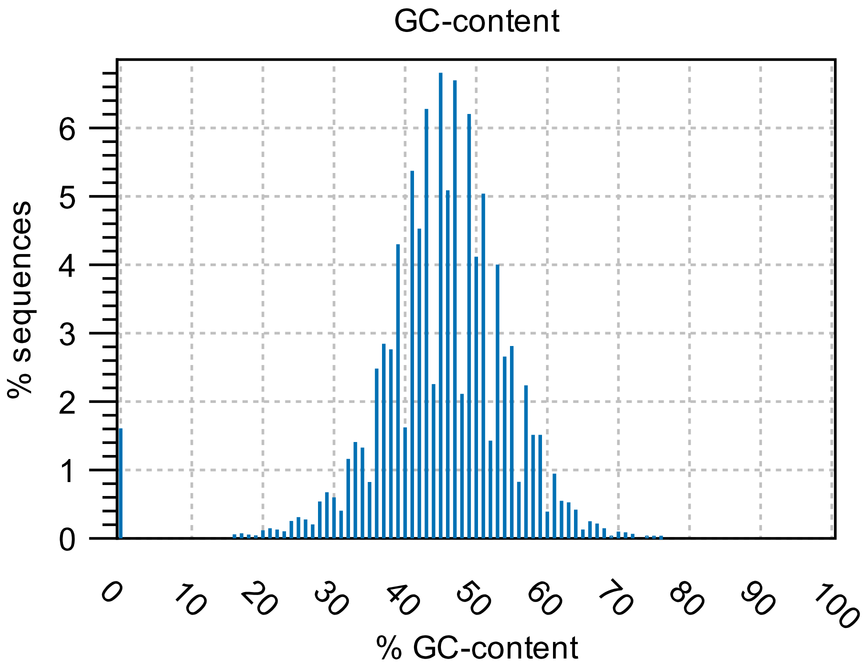

Distribution of GC-contents. The GC-content of a sequence is calculated as the number of GC-bases compared to all bases (including ambiguous bases).
x: relative GC-content of a sequence in percent
y: number of sequences featuring particular GC-percentages normalized to the total number of sequences

2.3 Ambiguous base-content

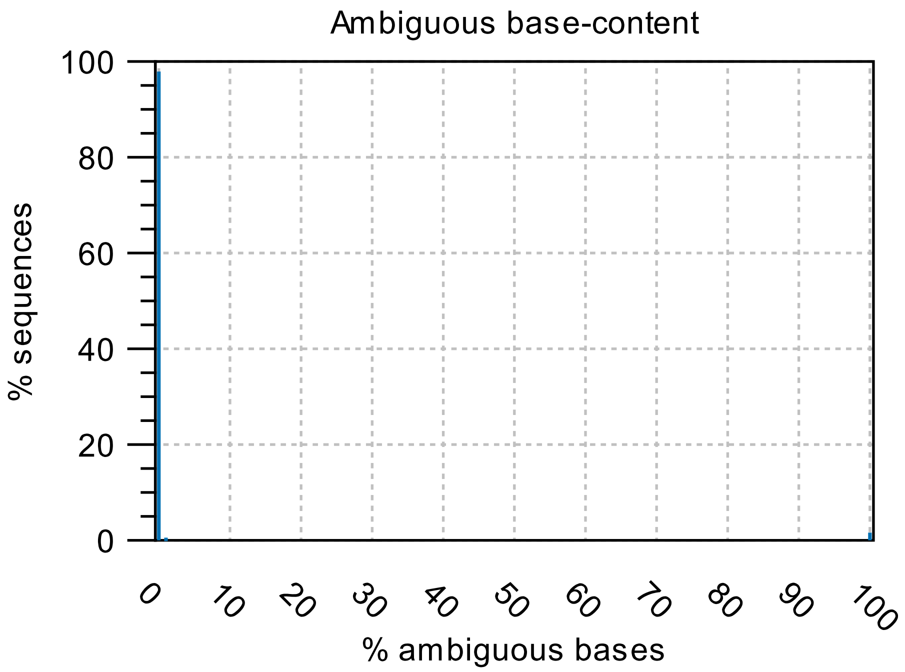

Distribution of N-contents. The N-content of a sequence is calculated as the number of ambiguous bases compared to all bases.
x: relative N-content of a sequence in percent
y: number of sequences featuring particular N-percentages normalized to the total number of sequences

2.4 Quality distribution

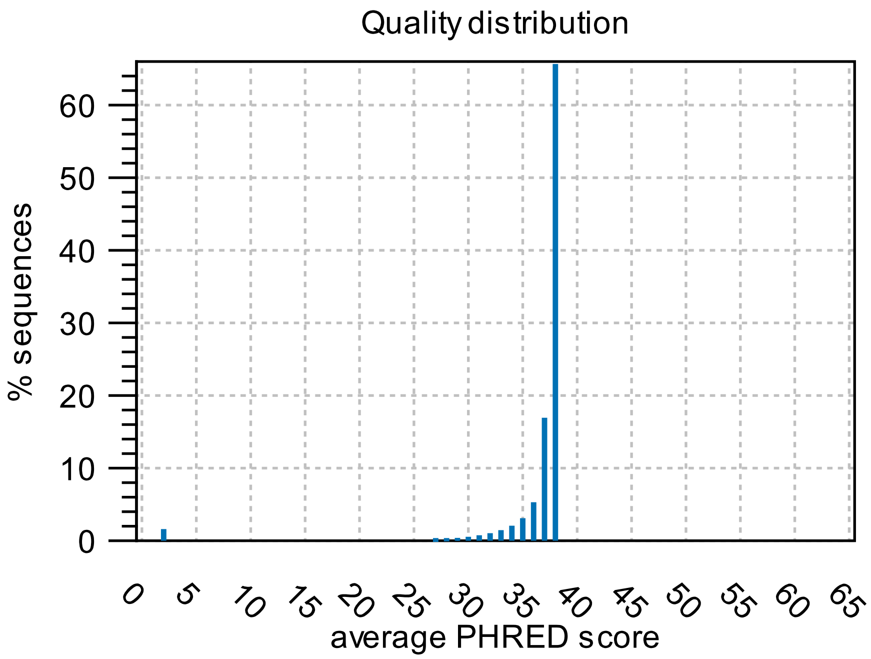

Distribution of average sequence qualitie scores. The quality of a sequence is calculated as the arithmetic mean of its base qualities.
x: PHRED-score
y: number of sequences observed at that qual. score normalized to the total number of sequences

3. Per-base analysis

3.1 Coverage

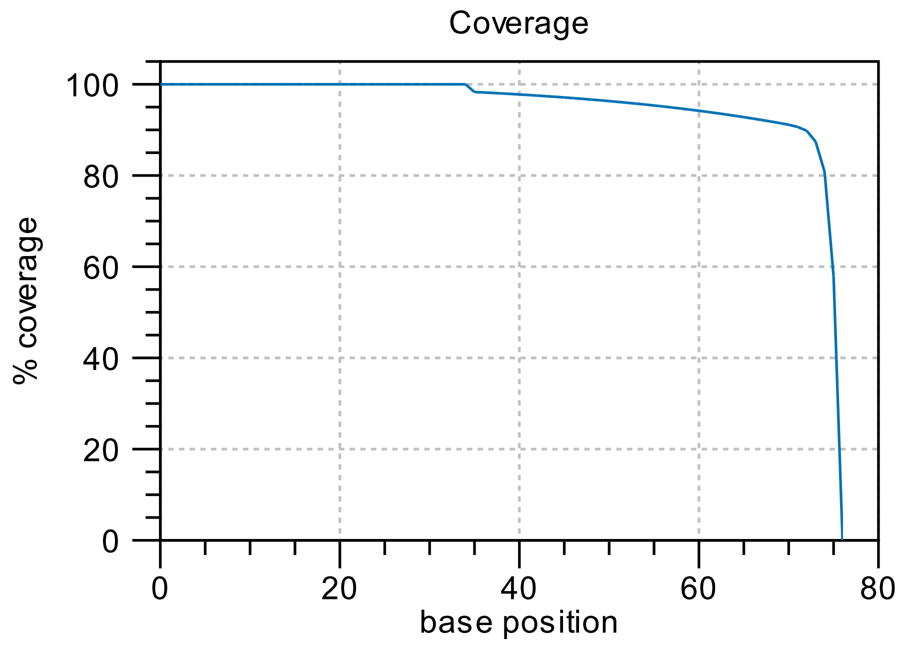

The number of sequences that support (cover) the individual base positions. In cases of untrimmed Illumina or SOLiD reads it will just contain a rectangle.
x: base position
y: number of sequences covering individual base positions normalized to the total number of sequences

3.2 Nucleotide contributions

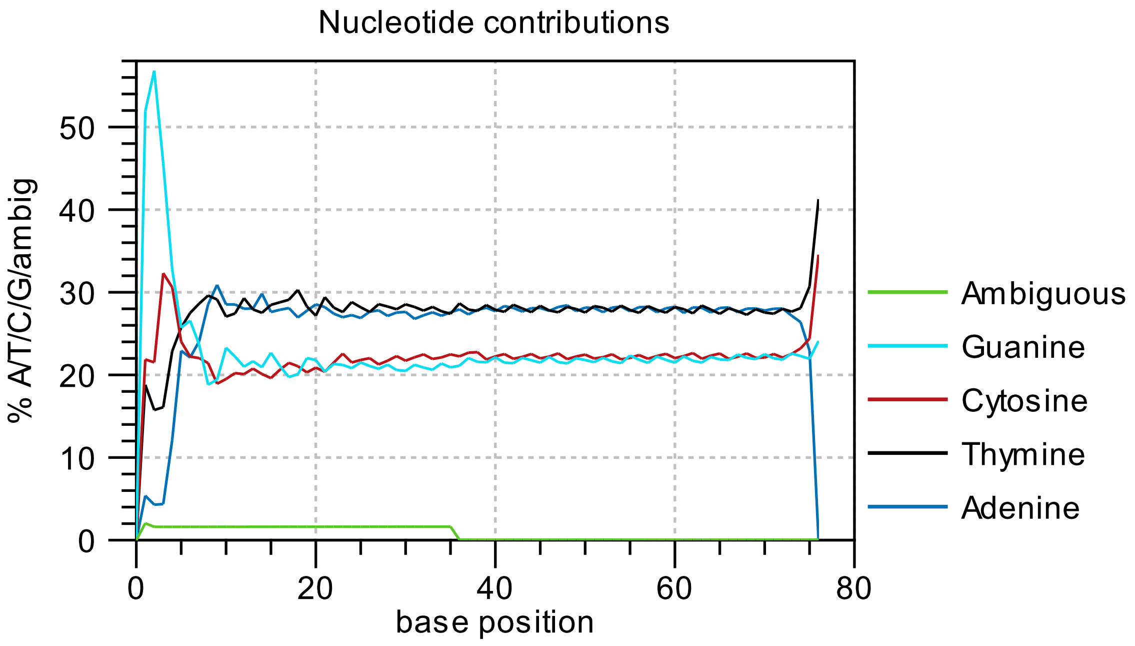

Coverages for the four DNA nucleotides and ambiguous bases.
x: base position
y: number of nucleotides observed per type normalized to the total number of nucleotides observed at that position

3.3 GC-content

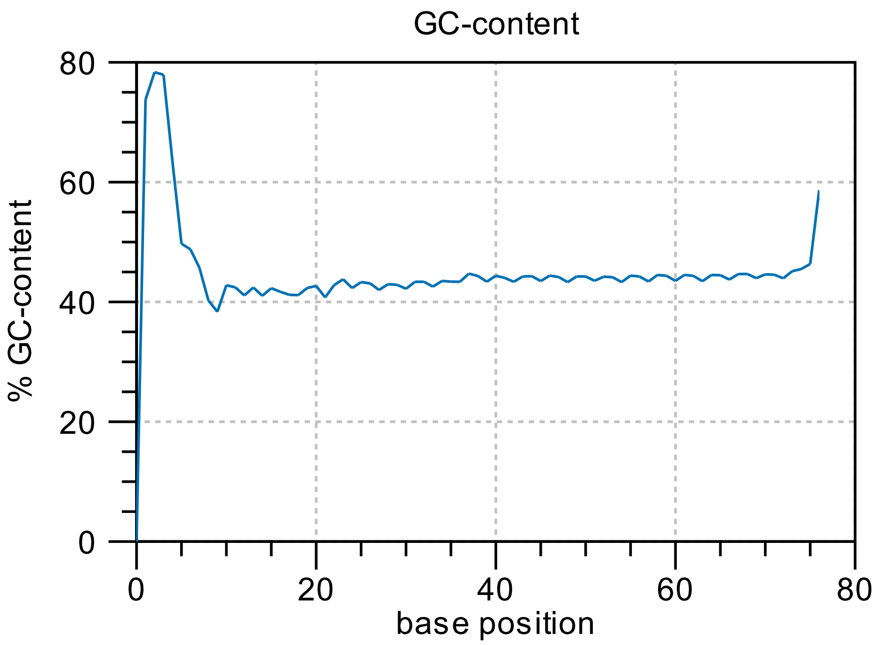

Combined coverage of G- and C-bases.
x: base position
y: number of G- and C-bases observed at current position normalized to the total number of bases observed at that position

3.4 Ambiguous base-content

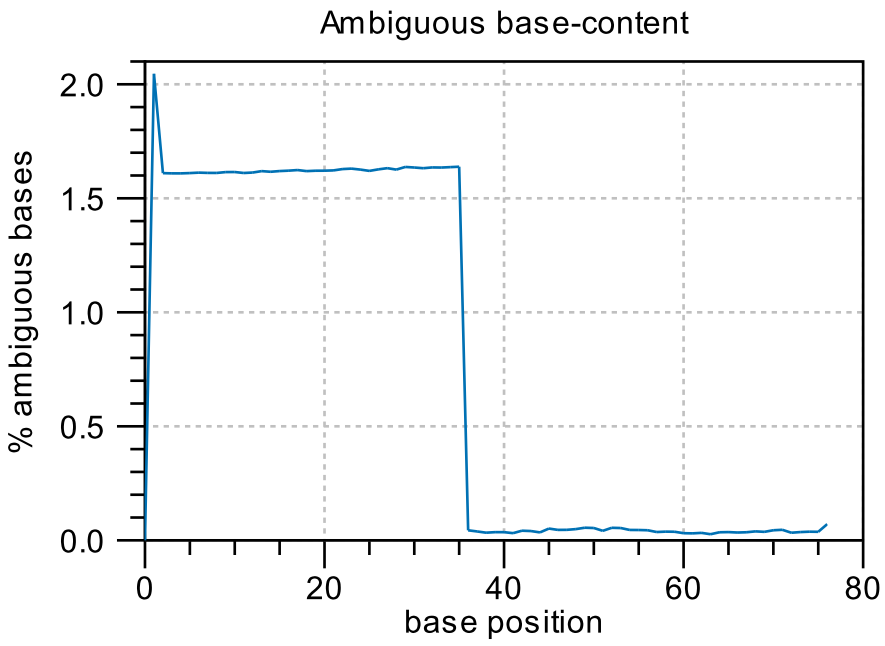

Combined coverage of ambiguous bases.
x: base position
y: number of ambiguous bases observed at current position normalized to the total number of bases observed at that position

3.5 Quality distribution

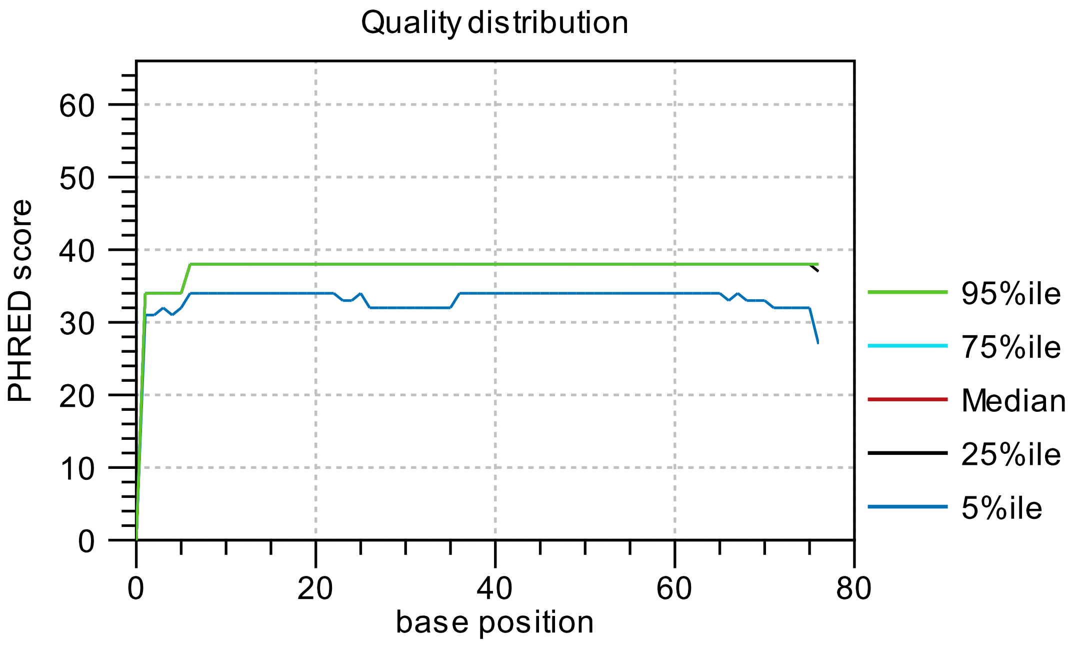

Base-quality distribution along the base positions.
x: base position
y: median & percentiles of quality scores observed at that base position

Sequencing QC Report
Based upon: 23,267,070 sequences in 6 data sets
Generated by: Guerrier
Creation date: Mon Nov 13 15:24:12 CET 2017
Software: CLC Genomics Workbench 9.0.1

Table of contents

1. Summary 3
2. Per-sequence analysis 3
    2.1 Lengths distribution 3
    2.2 GC-content 4
    2.3 Ambiguous base-content 5
    2.4 Quality distribution 6
3. Per-base analysis 6
    3.1 Coverage 7
    3.2 Nucleotide contributions 7
    3.3 GC-content 8
    3.4 Ambiguous base-content 9
    3.5 Quality distribution 9

1. Summary

Creation date:	Mon Nov 13 15:24:12 CET 2017
Generated by:	Guerrier
Software:	CLC Genomics Workbench 9.0.1
Based upon:	6 data sets
H20JA3_S9_L001_R1_001 (paired):	4,815,380 sequences in pairs
H20JA3_S9_L001_R1_001 (paired)-5:	3,553,118 sequences in pairs
H20JA3_S9_L001_R1_001 (paired)-4:	3,823,684 sequences in pairs
H20JA3_S9_L001_R1_001 (paired)-1:	3,229,548 sequences in pairs
H20JA3_S9_L001_R1_001 (paired)-2:	3,767,072 sequences in pairs
H20JA3_S9_L001_R1_001 (paired)-3:	4,078,268 sequences in pairs
Total sequences in data sets	23,267,070 sequences
Total nucleotides in data sets	1,714,689,277 nucleotides

2. Per-sequence analysis

2.1 Lengths distribution

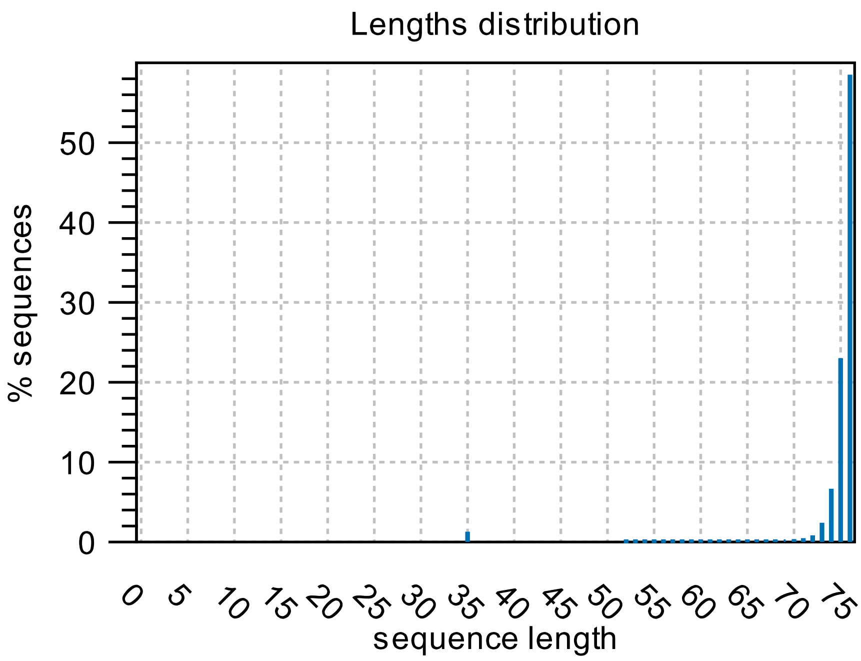

Distribution of sequence lengths. In cases of untrimmed Illumina or SOLiD reads it will just contain a single peak.
x: sequence length in base-pairs

y: number of sequences featuring a particular length normalized to the total number of sequences

2.2 GC-content

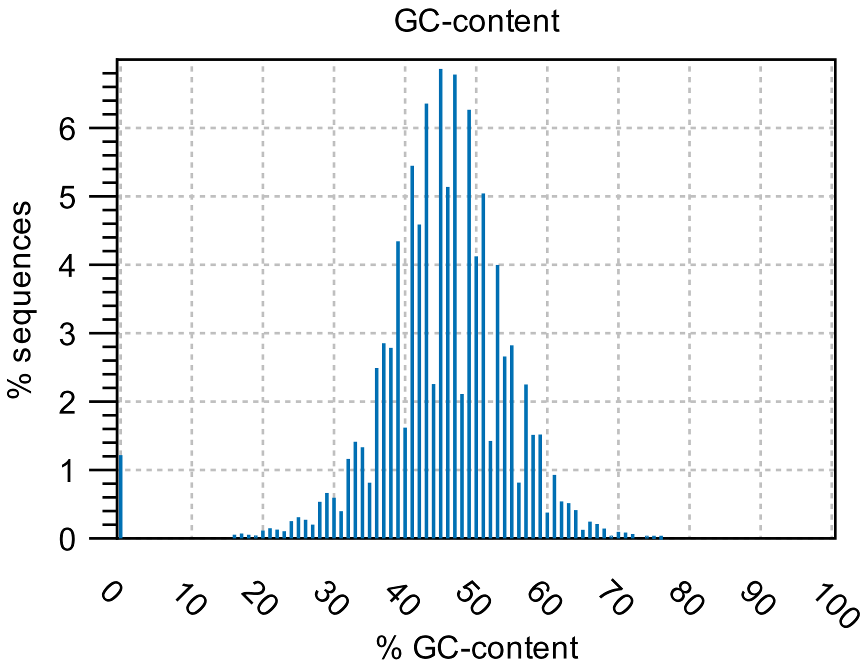

Distribution of GC-contents. The GC-content of a sequence is calculated as the number of GC-bases compared to all bases (including ambiguous bases).

x: relative GC-content of a sequence in percent

y: number of sequences featuring particular GC-percentages normalized to the total number of sequences

2.3 Ambiguous base-content

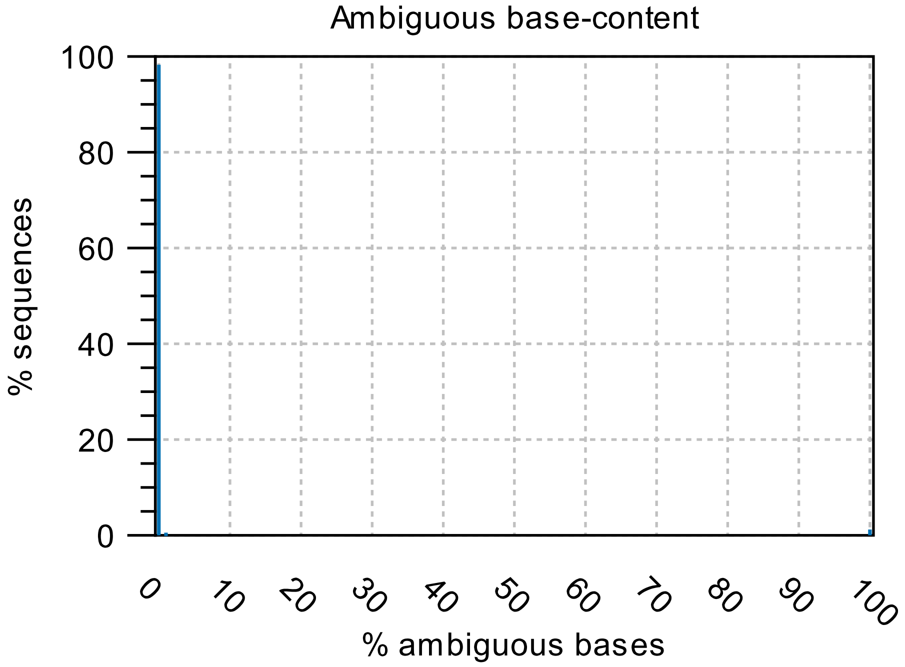

Distribution of N-contents. The N-content of a sequence is calculated as the number of ambiguous bases compared to all bases.
x: relative N-content of a sequence in percent
y: number of sequences featuring particular N-percentages normalized to the total number of sequences

2.4 Quality distribution

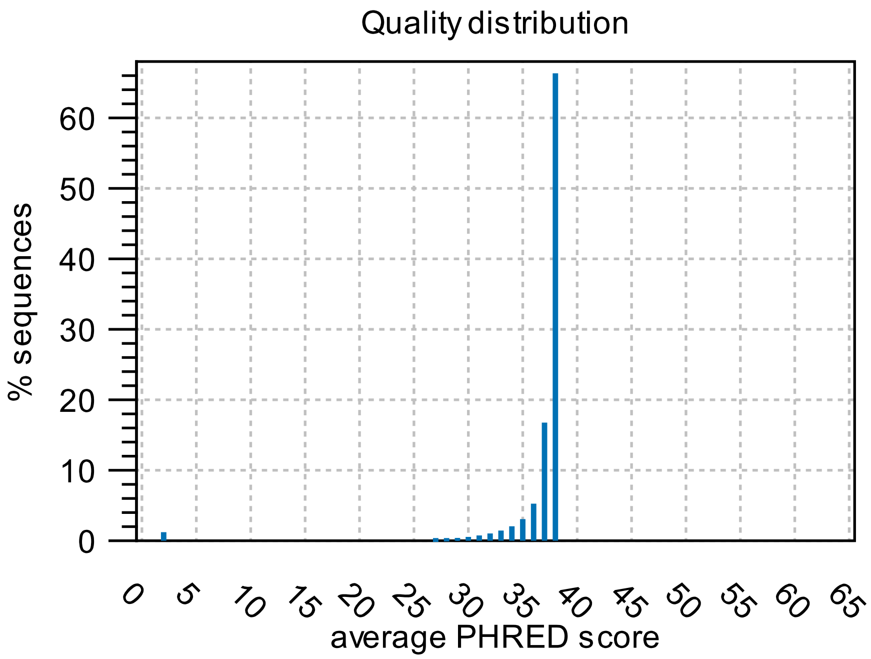

Distribution of average sequence qualitie scores. The quality of a sequence is calculated as the arithmetic mean of its base qualities.
x: PHRED-score
y: number of sequences observed at that qual. score normalized to the total number of sequences

3. Per-base analysis

3.1 Coverage

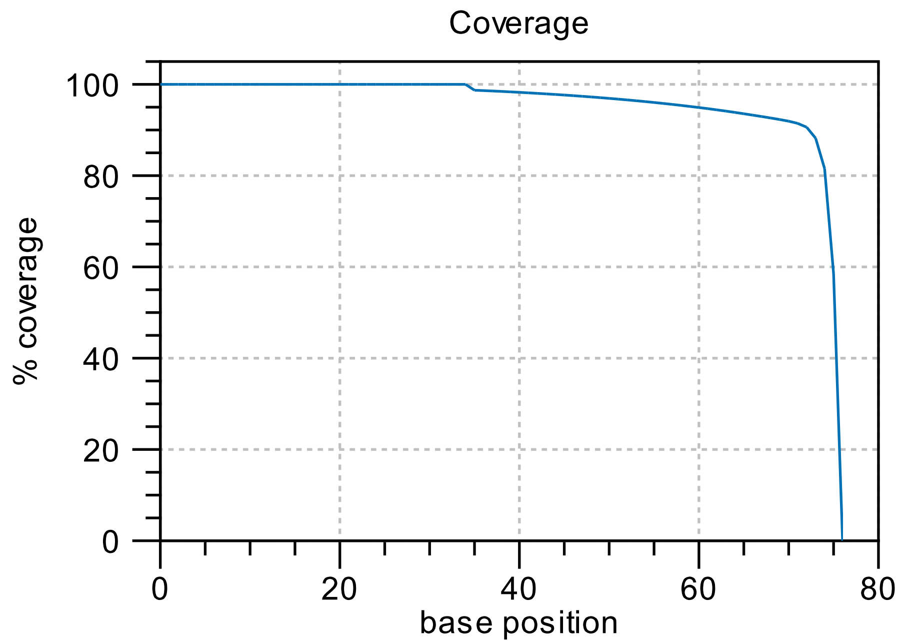

The number of sequences that support (cover) the individual base positions. In cases of untrimmed Illumina or SOLiD reads it will just contain a rectangle.
x: base position
y: number of sequences covering individual base positions normalized to the total number of sequences

3.2 Nucleotide contributions

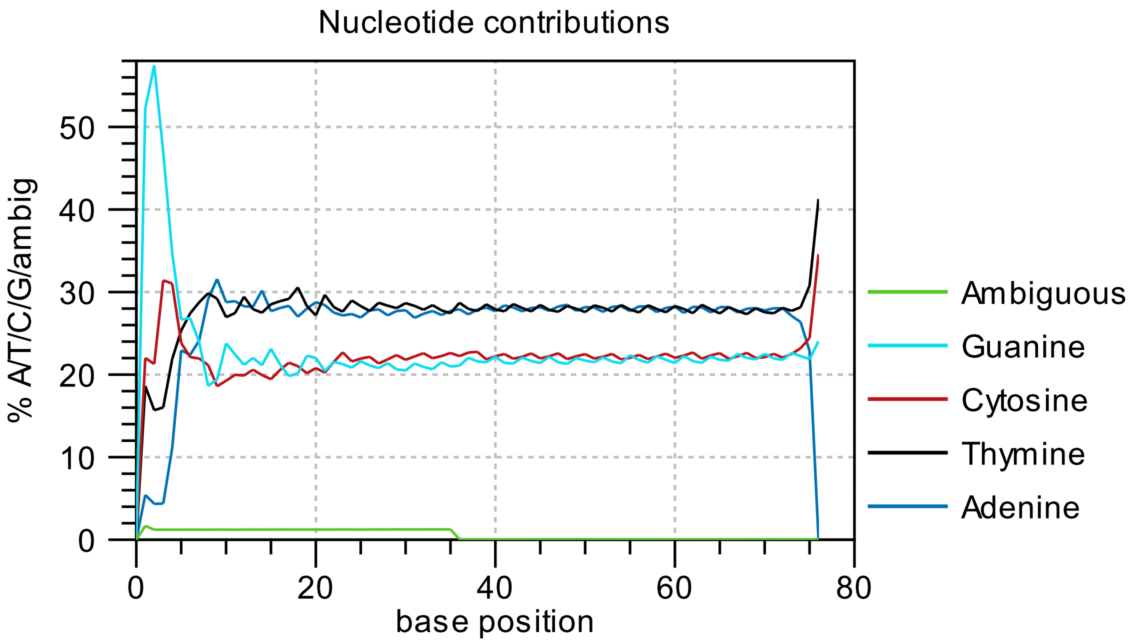

Coverages for the four DNA nucleotides and ambiguous bases.
x: base position
y: number of nucleotides observed per type normalized to the total number of nucleotides observed at that position

3.3 GC-content

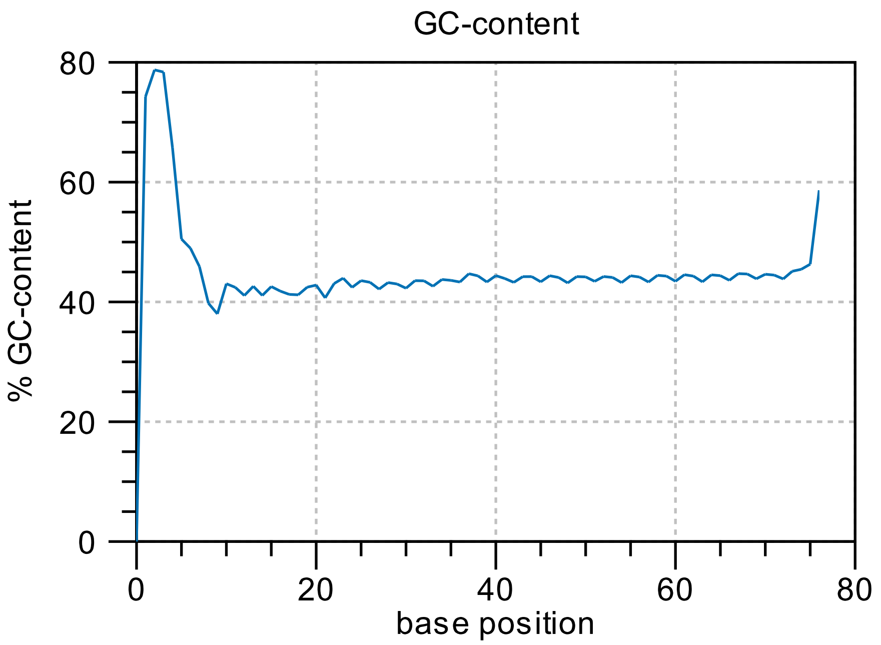

Combined coverage of G- and C-bases.
x: base position
y: number of G- and C-bases observed at current position normalized to the total number of bases observed at that position

3.4 Ambiguous base-content

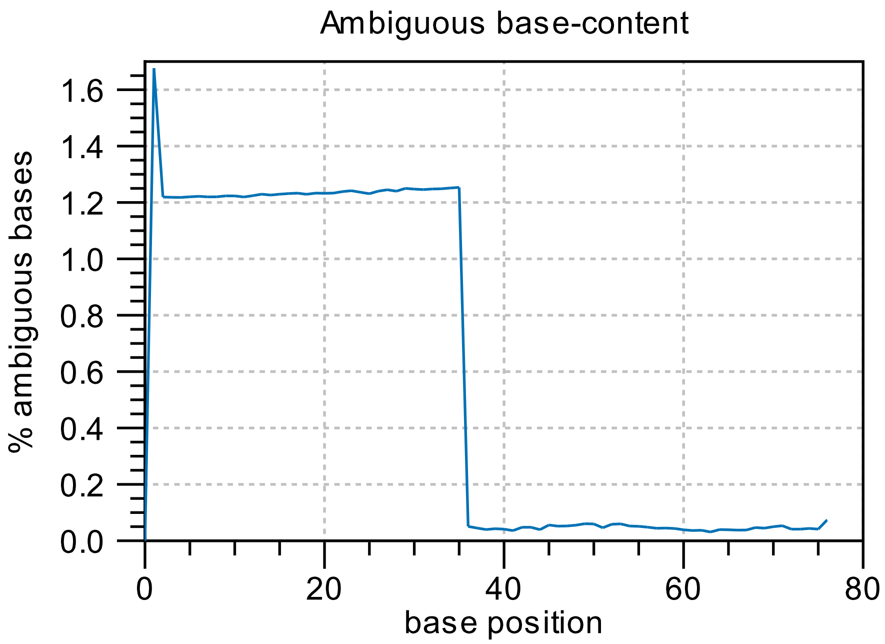

Combined coverage of ambiguous bases.
x: base position
y: number of ambiguous bases observed at current position normalized to the total number of bases observed at that position

3.5 Quality distribution

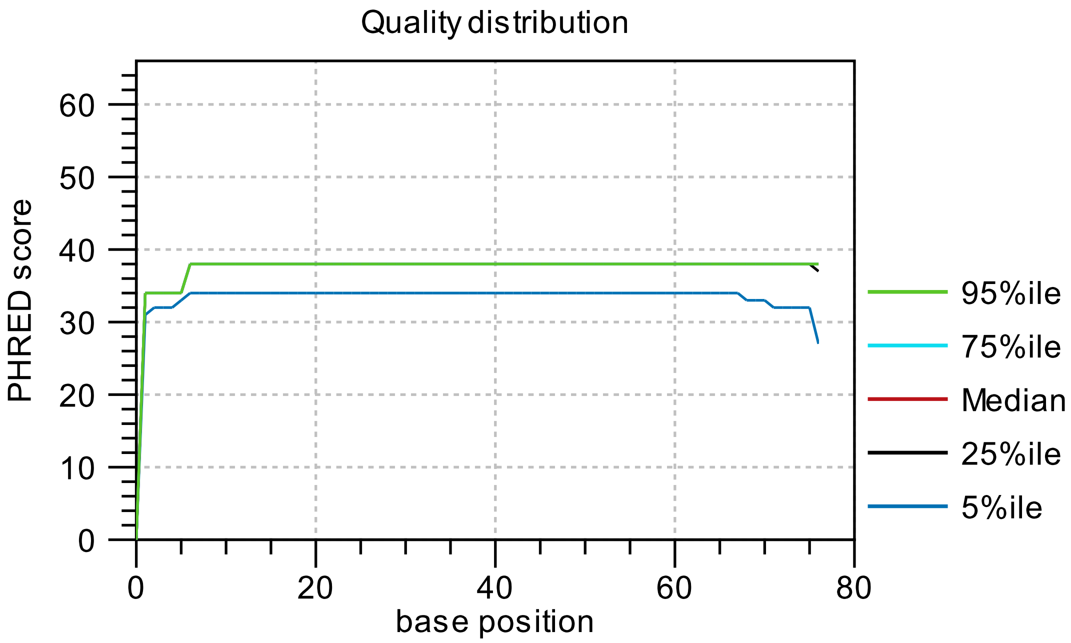

Base-quality distribution along the base positions.
x: base position
y: median & percentiles of quality scores observed at that base position
